# Trait divergence and trade‐offs among Brassicaceae species differing in elevational distribution

**DOI:** 10.1111/evo.14554

**Published:** 2022-07-20

**Authors:** Alessio Maccagni, Yvonne Willi

**Affiliations:** ^1^ Department of Environmental Sciences University of Basel Basel CH‐4056 Switzerland; ^2^ Botanical Garden of Canton Ticino Brissago CH‐6614 Switzerland

**Keywords:** Heat and frost stress, macroevolution, Ornstein‐Uhlenbeck, phylogenetic signal, range limits, thermal niche

## Abstract

Species have restricted geographic distributions and the causes are still largely unknown. Temperature has long been associated with distribution limits, suggesting that there are ubiquitous constraints to the evolution of the climate niche. Here, we investigated the traits involved in such constraints by macroevolutionary comparisons involving 100 Brassicaceae species differing in elevational distribution. Plants were grown under three temperature treatments (regular frost, mild, regular heat) and phenotyped for phenological, morphological, and thermal resistance traits. Trait values were analyzed by assessing the effect of temperature and elevational distribution, by comparing models of evolutionary trajectories, and by correlative approaches to identify trade‐offs. Analyses pointed to size, leaf morphology, and growth under heat as among the most discriminating traits between low‐ and high‐elevation species, with high‐elevation species growing faster under the occurrence of regular heat bouts, at the cost of reduced size. Mixed models and evolutionary models supported adaptive divergence for these traits, and correlation analysis indicated their involvement in moderate trade‐offs. Finally, we found asymmetry in trait evolution, with evolvability across traits being 50% less constrained under regular frost. Overall, results suggest that trade‐offs between traits under adaptive divergence contribute to the disparate distribution of species along the elevational gradient.

Species have restricted geographic distributions, but the causes behind this phenomenon are still unresolved (MacArthur 1972; Gaston 2003; Connallon and Sgrò 2018; Willi and Van Buskirk 2019). From an ecological point of view, range limits reflect dispersal limitation or limits of the ecological niche, with the niche being defined as the abiotic and biotic conditions that allow a species to persist (i.e., the realized niche sensu Hutchinson 1957; Leibold 1995). From an evolutionary point of view, range limits reflect limits to the evolution of the ecological niche. But why is it that species fail to adapt to environmental conditions beyond their current range? MacArthur (1972) suggested that a possible reason is exclusive divergent adaptation across habitats. He envisioned that specialization to one environment imposes high demographic costs under colonization of a new environment, or in other words, a trade‐off. Trade‐offs are a key concept in evolution, likely affecting all aspects of ecological specialization (Rosenzweig 1995) and applying to species distribution limits, but they have been rarely studied explicitly in this context (Willi and Van Buskirk 2022).

Among the many ecological factors that may affect the persistence of organisms, climate is known to be critical in controlling large‐scale distribution (MacArthur 1972). Many past studies noticed coincidences between geographic or elevational range limits and isotherms (Salisbury 1926; Iversen 1944; Dahl 1951; Root 1988). More recently, the field of species distribution modeling confirmed the good agreement between range limits and climate variables (e.g., Normand et al. 2009; Lee‐Yaw et al. 2016). Further studies looked into phenotypic patterns associated with the most limiting aspects of climate at range limits, particularly at the cold end of distribution. Loehle (1998) suggested that the northern range limit of North American tree species was determined by cold tolerance. Phenotypic data supported that species from higher latitudes were usually more tolerant to the cold than those from lower latitudes (Addo‐Bediako et al. 2000; Hawkins et al. 2014; Wen et al. 2018; Sunday et al. 2019). Similarly, abiotic stress appeared to be linked with the upper elevational range limit for some mountainous plant species, suggesting a predominant role of negative temperatures (Vetaas 2002; Macek et al. 2009; Körner et al. 2016). Also, the warm end of distribution may be strongly affected by climate, even though the prevailing hypothesis has emphasized the importance of negative species interactions (MacArthur 1972; Gaston 2003; Louthan et al. 2015). A recent literature review revealed that warm range limits were equally often affected by biotic interactions and abiotic conditions, whereas cold range limits were mainly affected by temperature (Paquette and Hargreaves 2021). However, because of the general dismissal of climate as a factor determining warm‐end limits, few studies focused on how organisms cope with heat in the context of species distribution limits (e.g., Sunday et al. 2012; Kellermann et al. 2012), particularly in plants (e.g., Kappen 1981; Wos and Willi 2015).

What are the sources of constraints in the evolution of the climate niche? According to simple evolutionary principles, genetic variation and selection are needed for a response to selection and adaptation (Falconer and Mackay 1996). Genetic constraints may involve low genetic variation of traits under selection. However, microevolutionary studies have shown that there is commonly ample genetic variation in single traits, and natural selection acting on populations is often strong (Mousseau and Roff 1987; Houle 1992; Kingsolver and Diamond 2011). These findings suggest generally rapid and ubiquitous adaptation through highly evolvable traits. Another type of genetic constraint is trade‐offs in fitness‐relevant traits, often seen as an obstacle to adaptive evolution by limiting the rate of adaptation (Futuyama and Moreno 1988; Bennett and Lenski 2007; Walker 2007). Negative genetic correlations among traits with regard to their fitness consequences appear mainly due to two nonexclusive causes. The first is that both the environment and the genetics of traits exert a limitation on trait values through differential allocation of limited amounts of resources (Bell 1984; van Noordwijk and de Jong 1986). The second cause is purely genetic; pleiotropic antagonism occurs when an allele increases the fitness via a first trait but reduces it via a second (Rose 1983). If we translate this into a thermobiology context, it is reasonable to assert that thermal extremes impose selection on some traits, resulting in a better thermal performance under one type of extreme, paid at the price of a reduction in performance in a contrasting environment or a contrasting aspect of the biology of the species. In ectothermic animals, relatively common trade‐offs involve thermal resistance on the one hand, and growth, starvation resistance, longevity, or reproduction on the other hand (Luckinbill 1998; Norry and Loeschcke 2002; Hoffmann et al. 2005; Stoks and De Block 2011; Casanueva et al. 2012), or cold and heat tolerance (Norry et al. 2007). Temperature can also mediate trade‐offs between traits, for example, between life span and reproduction (Mockett and Sohal 2006), or longevity and body size (Norry and Loeschcke 2002), or it can reverse the sign of a correlation (reviewed in Sgrò and Hoffmann 2004). In plants, trade‐offs were discovered between cold tolerance and frost resistance (e.g., in *Raphanus raphanistrum* [Agrawal et al. 2004]), and between speed of development and frost tolerance (Koehler et al. 2012; Molina‐Montenegro et al. 2012; Bucher et al. 2019).

Although microevolutionary studies can shed light on trade‐offs, those involving traits related to the climate niche have not revealed any cohesive patterns (e.g., Williams et al. 2012; Kelly et al. 2013). However, in the last decades, the field of comparative phylogenetics has developed macroevolutionary models that allow the study of adaptive evolution of more than one trait while accounting for the shared history among species (summarized in Garamszegi 2014). Based on comparative models, the phylogenetic signal of traits can be estimated and interpreted in the context of niche conservatism (Cooper et al. 2010). Furthermore, the contribution of different evolutionary processes and constraints to respond to selection can be inferred (Butler and King 2004). Three evolutionary processes are typically modeled. A first is *genetic drift*, by which inherited characters slowly change in random direction and accumulate differences over time. The process is typically modeled by Brownian motion (BM). A second process is *stabilizing selection*, a likely result of dependencies among characters under opposing selection (Wagner and Schwenk 2000). It is modeled by Ornstein‐Uhlenbeck (OU) diffusion, which constrains BM toward an optimal trait value. An extension allows for variation in the direction of OU diffusion across lineages, depicting the third process of *divergent selection* (OUM; Beaulieu et al. 2012). This approach has been used in evolutionary studies linking traits with the climate niche, particularly on plants, and they highlighted a link between life‐form or growth strategy and adaptation (or exposure) to a cold environment (Boucher et al. 2012; Kostikova et al. 2013; Tonnabel et al. 2018). Examples emphasize the great potential the approach has in detecting traits of adaptation to climate, and revealing potential trade‐offs in such adaptation or signatures of general evolutionary constraint.

Here, we studied trait divergence associated with the predominant elevational distribution of plant species and analyzed trait data for patterns of trade‐offs in a macroevolutionary framework. The study of elevational gradients is promising in the context for at least two reasons. On the one hand, elevation provides a steep climatic gradient in most mountainous regions, where over short geographic distances a reduction of the mean temperature of about 0.5 K per 100 m of elevation is found rather consistently (Körner 2003). On the other hand, species often occupy narrow elevational ranges (Körner 2003), making elevational gradients unique systems for studying adaptation to thermal stress and constraints in such evolution. Our study involved 100 Brassicaceae species occurring in the central Alps of Europe, with median elevational occurrence varying from 400 to 2800 m a.s.l. Seeds of the species were raised in climate chambers under three different temperature regimes (regular frost, mild, regular heat), and over a dozen traits representing growth, leaf morphology and coping with thermal extremes were measured. Four main hypotheses were tested: (i) species differ in trait expression depending on their elevational distribution; (ii) traits differ in the signature of past evolutionary processes having acted on them; (iii) phylogenetic conservatism in traits depends on the growth (thermal) environment; and (iv) there are trade‐offs among traits associated with adaptation to elevation.

## Material and Methods

### PLANT SPECIES

One hundred taxa (i.e., species and subspecies) belonging to the Brassicaceae family and naturally occurring in the Swiss Alps (and Jura) from the colline to the alpine life zone were selected. Apart from a good representation of the elevational gradient, other criteria were level of ploidy (diploid taxa preferred) and good representation of the phylogeny (list in Supporting Information SI1). In the general area, around 180 species of Brassicaceae occur, of which 28 are strictly high‐elevation species. On a global scale, Brassicaceae is an angiosperm family composed of 3700 species (including important agricultural cultivars) subdivided into three main lineages (Al‐Shehbaz et al. 2006).

For this study, seeds were collected from March to September during the years 2015–2017 at two different sites for each species within Switzerland. The sites were around the most common elevation for each species, at least 50 km apart from each other, and preferentially from different biogeographic regions (Jura, Plateau, northern Prealps, western and eastern central Alps, and southern Prealps). For plants with very restricted distributions, only one population was sampled, but the number of individuals was doubled. At each site, seeds were collected from 10 to 30 different mother plants over an area of usually 50 m^2^ and spaced out from each other by 5 m. For endangered species on the Red List 2002 for Switzerland (Moser et al. 2002), authorization for sampling was obtained from the respective Cantonal authority. Sampled seeds of each mother plant were stored in separate paper bags under cold (4°C), dark, and dry (added silica gel) conditions until sowing.

### RAISING OF PLANTS UNDER THREE GROWTH TREATMENTS AND TRAIT ASSESSMENT

#### Design

The experimental design involved the raising of 100 taxa, each represented by two populations and three maternal lines per population (or one population with six maternal lines), that is, six maternal lines per species. The experiment was split into six blocks, with a different maternal line per species in a block. Within block, plants of a maternal line were exposed to three temperature treatments (regular frost, mild, regular heat). The final design resulted in 1800 individuals (100 taxa × 6 maternal lines each in a different block × 3 treatments = 1800 individuals). Maternal lines of a population were selected randomly, and seeds of a maternal line were selected haphazardly. A first round of sowing (S1) was done without the use of gibberellic acid (GA_3_), resulting in some species (20) not germinating and some heterogeneity in the timing of germination. In a second round of sowing (S2), seeds were treated with gibberellic acid (GA_3_), resulting in the germination of 14 additional species (but five were now lacking that germinated in S1) and a more similar timing of germination.

#### Plant rearing

Seeds were germinated in climate chambers under controlled conditions, with similar procedures in S1 and S2 (S2 described in detail below). Two seeds were placed in a 1.5‐mL Eppendorf tube filled with 500 μL of GA_3_ solution (500 ppm, Merck KGeA, Dornstadt, Germany), with three tubes per maternal line. Seeds were incubated for 1 week in dark and cold (4°C constant in Climecabs; Kälte 3000, Landquart, Switzerland) and then sown in multipot trays (0.06 L, 54 pots per tray with Ø 4.4 cm each, BK Qualipot; gvz‐rossat.ch, Otelfingen, Switzerland). Each pot had been filled with a mixture of soil (bark compost, peat and perlite, Aussaat‐ und Pikiererde; Oekohum, Herrenhof, Switzerland) and sand (0–4 mm) in a ratio of 2:1. Multipot trays were covered with a garden fleece (Windhager, Hünenberg, Switzerland) and set up in blocks within growth chambers (MobyLux GroBanks; CLF Plant Climatics, Wertingen, Germany). Growth chambers were located inside a PlantMaster (CLF Plant Climatics) with managed humidity and temperature. Trays were kept at 18°C during daytime (8 h) and 15°C during nighttime (16 h), at 75% relative humidity (RH), and a light intensity of 150 μmol m^–2^ s^–1^ (fluorescent white lamps and red‐LED). Twice a week, blocks were moved to a different chamber, with re‐randomized positioning of trays. After 3 weeks, excess seedlings were used to fill pots with no germination with the following priority: use of the same maternal line within block, or the same population, or the same species. In week 4, germinated plants were moved back to climate chambers and entire trays were subjected to one of three temperature treatments.

#### Treatment

The three temperature treatments were as follows: “Frost” (F), “Mild/control” (M), and “Heat” (H). Conditions of the treatments were the following: Frost: 20°C (daytime), then –2°C for 1 h (–4.8 K h^–1^; nighttime) and back to 20°C (+7.3 K h^–1^; night); Mild: 20°C constant; and Heat: 20°C (beginning of day), then 40°C for 1 h (+5 K h^–1^; day), back to 20°C (–8.3 K h^–1^; day), 20°C (night). All treatments were conducted at cycles of 12:12 h light:dark and a light intensity of about 300 μmol m^–2^ s^–1^ (LED white lamp) and 75% RH. Plants were acclimated 2 days before the beginning of treatment by exposing them to milder extremes, 2°C for the frost treatment, and 35°C for the heat treatment. We selected extreme temperatures based on records in the field during the growing season (Larcher and Wagner 1976; Sutinen et al. 2001; Körner 2003), whereas for the mild treatment we used a common standard temperature. Trays were randomized daily within each block, whereas blocks were moved to different climate chambers twice a week. Plants were kept under these conditions until the 9th week after sowing, when trait assessment was performed. Mean species numbers across blocks that were assessed for a particular trait within the treatments ranged from 82.1 ± 3.6 (Heat) to 85.5 ± 3.5 (Mild) in S2 (*N* = 1406 plants), and from 52.1 ± 24.0 (Heat) to 74.6 ± 1.1 (Mild) in S1 (*N* = 862 plants).

#### Traits

Two traits were assessed before treatment started: seed size (SSIZ, in mm^2^) and days to germination (TGER). Five traits characterized the trajectory of plant growth mainly based on leaf length: the initial growth rate (IGR, in mm day^–1^), parameters of a three‐parameter logistic model including the maximal growth rate (MGR, scale^–1^), the time to half the asymptotic size (and fastest growth; XMID, in days) and asymptotic size (ASYM, in mm), and finally the number of leaves on day 35 of treatment (NLEA). Because smaller values of XMID meant that a plant achieved mid‐size faster, values were multiplied by –1 ([–]XMID) to represent progression of growth. Five leaf functional traits were assessed: leaf area (LA, in mm^2^), specific leaf area (SLA, area over dry weight in mm^2^ mg^–1^), leaf dry matter content (LDMC, ratio of dry weight over fresh weight in mg g^–1^), leaf thickness (LTh, in mm), and leaf dissection index (LDI, no unit). Resistance of leaves to thermal extremes was assessed under –10 or –11°C ([–]T2) and –5 or –6°C ([–]T1), and +45 or +47°C ([+]T1) and +50 or +51°C ([+]T2). Resistance to T1 and T2 was tested on nonacclimated plants (i.e., plants of the mild growth treatment) and acclimated plants (i.e., plants pre‐exposed to frost for assessing frost resistance, and plants pre‐exposed to heat for assessing heat resistance), with some exceptions in the two rounds of sowing. Tolerance to repeated frost or heat during the growth phase was calculated as MGR, (–)XMID, or ASYM under frost or heat treatment minus the respective estimate in the mild treatment, divided by the estimate in the mild treatment. We used the term frost/heat tolerance sensu lato (s.l.) to refer to tolerance and resistance together. Full details on trait assessment are given in Supporting Information SI2. For analyses, means of replicate trait measures per plant were calculated, on which species means per treatment and round of sowing (for mixed‐model analysis) and finally species means per treatment across rounds of sowing were calculated (for evolutionary models).

### STATISTICAL ANALYSIS

#### Trait expression differing with temperature treatment during growth and elevational distribution

The effect of temperature treatment and median elevation of species distribution on traits was tested using Bayesian generalized linear mixed models and Markov Chain Monte Carlo techniques with the function “brm” of the R package {brms} (Bürkner 2017). The fixed effect of treatment was coded as a categorical variable, and contrasts were performed against the “Mild” treatment or, for tolerance, against “Frost.” The fixed effect of median elevation of species distribution was calculated based on reported species occurrences of a nation‐wide species inventory (infoflora.ch). Median elevation was mean‐centered prior to analyses. Random effects were the round of sowing (i.e., S1 and S2) and the relatedness among species. A phylogeny produced based on several dozen chloroplast genes (Patsiou et al. 2021) was pruned to species included in this study with the function “treedata” {geiger} (Harmon et al. 2008). The final matrix was obtained with the function “vcv” {ape} (Paradis and Schliep 2019) and called with the “cov_ranef” argument in brm. For each model, the contribution of the phylogenetic effect was tested by comparing the model that included it as a random effect to one that did not. Model comparisons were performed using the leave‐one‐out cross validation (i.e., LOO), which was calculated with the function “add_criterion” {brms} combined with the expected log pointwise predictive density (i.e., ELPD) with the function “loo_compare” {brms}. Resistance traits were modeled assuming a beta distribution because of their constrained nature between 0 and 1 (i.e., 100%), (–)XMID and tolerances with a Gaussian distribution, and the remaining traits with a log‐normal distribution. Sampling behavior of MCMC was inspected visually, and number of iterations, warmup and sampling interval adapted to each model to retain an effective sampling size of 1000. Significance was tested by probability of direction calculated with the function “p_direction” {bayestestR} (Makowski et al. 2019). All analyses and figures were done with the statistics software R version 4.0.3 (R Core Team 2014), and calculations were performed at sciCORE (http://scicore.unibas.ch/) scientific computing center of the University of Basel.

#### Past evolutionary forces

Phylogenetic analyses on the evolutionary processes that had shaped trait divergence among species were run separately for the three temperature treatments, and by considering variance in trait means of species of the two rounds of sowing. We tested five evolutionary models using the R packages {geiger} and {mvMORPH} (Clavel et al. 2015): white noise (WN) with trait evolution independent of phylogeny, BM1 with some intensity of random fluctuations, BMM with different intensities of random fluctuations between regimes, OU1, and OUM. For BMM and OUM, the contrasting environmental regimes were low‐ versus high‐elevation distribution of species. Assignment to one of the two classes was made using the InfoFlora (infoflora.ch) distribution information, with a threshold at 1500 m a.s.l. (splitting species of the foothills/hills from those of sub‐/alpine areas). For less frequent species on Swiss territory, the assignment was verified by data of the entire Alps and neighboring mountain massifs (based on Aeschimann et al. 2004). Model comparison was based on the Akaike information criterion with a correction for small sample size (AICc) and took into account uncertainty in the estimation of ancestral states of niche parameters. Details on ancestral state reconstruction and model comparison are described in SI2. Validation of the results was performed by simulations on synthetic data and analyses after the random removal of species (SI2).

Phylogenetic half‐life, that is, the time required for a trait to evolve halfway toward its adaptive optimum, was calculated for all traits assessed in the three growth environments and for each evolutionary model described above. Values were extracted from an OU1 model, except when elevation had a significant effect—either in mixed models or evolutionary analysis; in those cases, values were derived from an OUM process. Small values of half‐life indicate fast adaptation toward the optima and a lack of phylogenetic inertia, whereas high values indicate that traits retain the influence of the ancestral states for a longer time. We tested for an effect of growth environment (a factor with three levels, with “Mild” as baseline) on the evolutionary lability of traits with a generalized linear mixed model with “brm” (as specified above). Phylogenetic half‐life was modeled assuming a log‐normal distribution, and trait was a random effect.

#### Multi‐trait relationships and trade‐offs

To identify putative trade‐offs between pairs of traits measured in the three growth environments, Pearson correlation coefficients were calculated using the function “rcorr” {Hmisc} (Harrell 2019). Before performing correlations, some traits were log_10_‐transformed (i.e., SSIZ, MGR, NLEA, LA, LDI, and RES[+]T2), and all traits were centered to a mean of zero and scaled to the variance. Then, highly collinear traits were removed from the dataset using the function “vifstep” {usdm} with a threshold of 10, which resulted in the drop of 10 traits (i.e., ASYM_Frost_, ASYM_Heat_, NLEA_Mild_, NLEA_Heat_, LA_Frost_, LA_Heat_, SLA_Frost_, LDI_Mild_, LDI_Heat_, and TOL_IGR_Frost_). To further reduce the number of traits while maintaining the most discriminating ones in regard to the elevation of origin of species, discriminant analysis of principal components (DAPC) was performed with the function “dapc” {adegenet} (Jombart 2008). The optimal number of PCs to retain was selected based on stratified cross validation with the function “xvalDapc” {adegenet} and 10,000 simulations for each level of PC retention. Traits contributing with a loading higher than 0.024 (i.e., third quartile of variable contribution) were selected and used for correlation analysis.

## Results

### TRAIT EXPRESSION DIFFERING WITH TEMPERATURE TREATMENT DURING GROWTH AND ELEVATIONAL DISTRIBUTION

**Figure 1:**
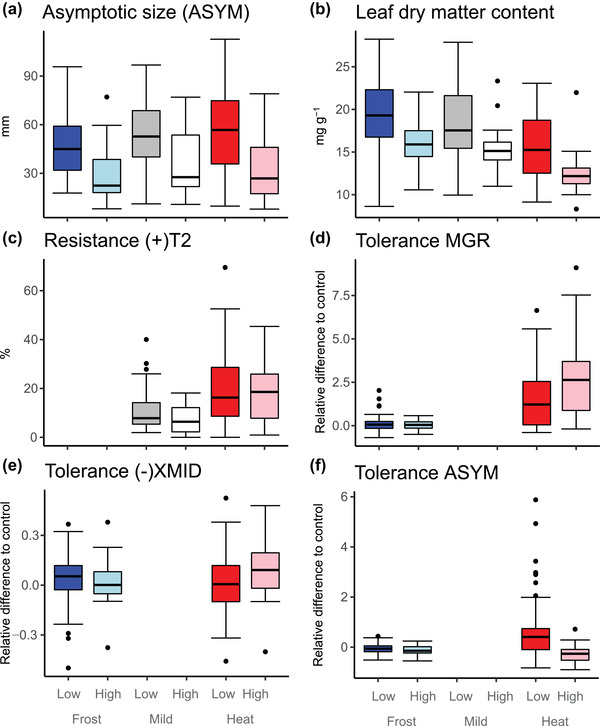
Boxplot showing the distribution of species‐mean trait values for which species differed depending on their median elevation (low vs. high elevation), either across growth treatments or in a particular growth treatment (regular frost, mild, regular heat). For simplicity, only data of the second round of sowing are included and traits for which mixed‐effects models and evolutionary models produced concordant results (data of both rounds of sowing and all traits shown in Fig. S2). Colors inside boxes represent the treatments (blue for Frost, grayscale for Mild, and red for Heat), whereas the intensity represents median elevation of species occurrence (dark colors for low elevation, light colors for high elevation). The thick horizontal line is the median, and the lower and upper hinges are the 25th and 75th percentiles; whiskers extend from the hinges to the most extreme data points within 1.5 × IQR, and dots are values beyond those ranges.

Results on trait expression differing among growth treatments and species depending on their elevational distribution are summarized in Table 1 and Figure 1 (and Supporting Information SI3). A high fraction of traits (∼70%) responded to temperature. Under regular frost compared to mild conditions, plants reached the midpoint of final size earlier (Fig. S2e), but they had smaller asymptotic size (Fig. 1a) and fewer and smaller leaves (Fig. S2g,h). Their leaves had less surface area per dry mass and were thicker (smaller SLA and larger LTh; Figs. 1b and S2k). However, frost resistance of leaves was not significantly different after pre‐exposure to frost during growth (Fig. S2r). Under regular heat during growth compared to mild conditions, the maximal growth rate of plants was significantly higher, the time to maximal growth shorter (Fig. S2d,e), and plants had smaller asymptotic size (Fig. 1a) and smaller leaves (Fig. S2h). Furthermore, leaves had more surface area per dry mass and less dry mass per wet weight (larger SLA and smaller LDMC; Figs. S2i and 1b). Finally, tolerance to heat was generally higher compared to tolerance to frost for maximal growth rate and asymptotic size (Fig. 1d,f).

**Table 1:** Results of mixed‐effects models on the relationship between median elevation of species distribution, treatment during plant growth (regular frost, mild, regular heat), and their interaction on plant traits

			Treatment		Elevation	Treatment × elevation	
Trait	Trait abb.		Frost vs. Mild	Heat vs. Mild		Slope of elevation under Frost vs. Mild	Slope of elevation under Heat vs. Mild
Seed size φ	SSIZ				0.058 [–0.166, 0.028]		
Time to germination	TGER				0.043 [–0.017, 0.101]		
Growth							
Initial growth rate	IGR		–0.006 [–0.033, 0.021]	–0.019 [–0.047, 0.008]	–0.009 [–0.027, 0.011]	0.012 [–0.015, 0.040]	0.015 [–0.011, 0.044]
**Maximal growth rate**	**MGR**		–0.041 [–0.126, 0.037]	**0.422 [0.338, 0.500]** ^***^	–0.030 [–0.093, 0.020]	0.013 [–0.072, 0.089]	0.129 [0.046, 0.211]^*^
**(–) Time to fastest growth** φ	**(–)XMID**		**1.033 [0.447, 1.594]** ^**^	**2.114 [1.534, 2.722]** ^***^	–0.702 [–1.292, –0.105](.)	0.357 [–0.276, 0.961]	**1.491 [0.899, 2.067]** ^***^
**Asymptotic size** φ	**ASYM**	Fig.1a	**–0.139 [–0.185, –0.091]** ^***^	–0.153 [0.200, –0.103]^***^	–0.040 [–0.111, 0.031]	0.025 [–0.072, 0.023]	**–0.134 [–0.184, –0.086]** ^***^
Number of leaves _S2_, φ	NLEA		–0.064 [–0.111, –0.012]^*^	0.005 [–0.043, 0.055]	–0.011 [–0.107, 0.086]	–0.008 [–0.57, 0.042]	–0.025 [–0.071, 0.025]
Leaf traits							
**Leaf area** φ	**LA**		**–0.162 [–0.253, –0.086]** ^**^	–0.255 [–0.338, –0.170]^***^	**–0.349 [–0.483, –0.218]** ^***^	0.033 [–0.051, 0.120]	–0.117 [–0.203, –0.028]^*^
Specific leaf area φ	SLA		**–0.130 [–0.184, –0.075]** ^***^	**0.112 [0.056, 0.167]** ^***^	0.018 [–0.036, 0.075]	–0.030 [–0.083, 0.025]	0.018 [–0.040, 0.072]
**Leaf dry matter content** φ	**LDMC**	Fig.1b	0.042 [0.006, 0.075](.)	**–0.182 [–0.216, –0.146]** ^***^	**–0.066 [–0.106, –0.025]** ^**^	0.008 [–0.027, 0.045]	–0.019 [–0.55, 0.018]
Leaf thickness _S2_, φ	LTh		**0.077 [0.030, 0.126]** ^**^	–0.017 [–0.064, 0.031]	0.026 [–0.016, 0.066]	–0.011 [–0.058, –0.040]	0.0004 [–0.049, 0.050]
**Leaf dissection index** φ	**LDI**		–0.009 [–0.032, 0.012]	–0.017 [–0.041, 0.005]	0.039 [–0.002, 0.077]	0.019 [–0.003, 0.042]	–0.045 [–0.069, –0.022]^**^
Thermal tolerance s.l.							
Frost resistance							
*…acclimated (1 h at –6°C)*_S1_	RES(–)T1				–0.080 [–0.209, 0.057]		
*…nonacclimated (1 h at –5°C)*_S2_	RES(–)T1				0.032 [–0.042, 0.106]		
*…acclimated (1 h at –11°C)*	RES(–)T2				0.123 [0.021, 0.234](.)		
*…nonacclimated (1 h at –10°C)*_S2_	RES(–)T2				0.043 [–0.064, 0.160]		
**Heat resistance**							
*…acclimated (1 h at +47°C)*_S1_, φ	RES(+)T1				–0.029 [–0.174, 0.121]		
*…nonacclimated (1 h at +45°C)*_S2_	RES(+)T1				0.044 [–0.046, 0.133]		
*…acclimated (1 h at +51°C)*	RES(+)T2				0.026 [–0.134, 0.183]		
***…nonacclimated (1 h at +50°C)***_S2_	**RES(+)T2**	Fig. 1c			**–0.176 [–0.317, –0.038]** ^*^		

				Heat vs. Frost	Elevation		Slope of elevation under Heat vs. Frost
Tolerance IGR	TOL_IGR			0.002 [–0.038, 0.041]	0.013 [–0.015, 0.042]		0.012 [–0.029, 0.051]
**Tolerance MGR** φ	**TOL_MGR**	Fig. 1d		**1.495 [1.287, 1.701]** ^***^	0.083 [–0.128, 0.293]		0.315 [0.119, 0.523]^*^
**Tolerance (–)XMID**	**TOL_(–)XMID**	Fig. 1e		0.017 [–0.011, 0.049]	–0.008 [–0.019, 0.032]		0.048 [–0.018, 0.077]^**^
**Tolerance ASYM** φ	**TOL_ASYM**	Fig. 1f		0.411 [0.288, 0.540]^***^	0.013 [–0.107, 0.142]		–0.303 [–0.427, –0.176]^***^

Posterior median (with 90% highest density interval [HDI]) of fixed effects are reported, relative to the baseline of average elevation and mild growth conditions (full details in Table S1, including results on random effects). For tolerance traits, the coefficients express differences between estimates under heat compared to those under frost. Significant effects are highlighted in bold (HDI not overlapping with 0, and probability of direction (pd) >97.5% [(.) pd > 95, ^*^ pd > 97.5%, ^**^ pd > 99.5%, ^***^ pd > 99.95]). Traits for which either elevation or an interaction term was significant are also highlighted in bold (trait names). Furthermore, traits for which the model accounting for phylogeny was better supported (φ) or not (φ_N_) are indicated; when nothing is reported, no statistical difference between models was found. If not specified, a trait was assessed both in sowing round S1 and in S2.

Median elevation of species distribution alone explained only significant variation in the general expression of three traits (Table 1). Species occurring at higher elevation had smaller leaves (Fig. S2h), lower dry matter content (Fig. 1b), and lower heat resistance under no acclimation (RES[+]T2; Fig. 1c). A considerable fraction of traits was significantly affected by an interaction between median elevation of distribution and treatment, but only in the comparison between mild conditions and the heat treatment. The only notable exception was that higher elevation species had increased frost resistance (after acclimation), but only for the first round of sowing and when exposed to the cooler of two frost treatments (Fig. S2r). When exposed to heat, higher compared to lower elevation species had higher growth rates (Fig. S2d), reached maximal growth earlier (higher [–]XMID; Fig. S2e), but ended up being smaller (Fig. 1a), with smaller and less dissected leaves (Fig. S2h,l). In line, higher elevation species showed heightened tolerance to heat—compared to frost—by having a faster maximum growth (Fig. 1d), which was reached earlier (Fig. 1e), but they also showed lower tolerance to heat by ending up being smaller (Fig. 1f). Comparisons between models with and without considering the phylogeny revealed that its inclusion improved the model for about 70% of traits (Tables 1 and S2).

## PAST EVOLUTIONARY FORCES

Table 2 summarizes results of analyses on the evolutionary processes having acted on traits, for each growth environment (for a full account, see Table S3 in Supporting Information SI4). The comparison between the two evolutionary switch models (i.e., equal rate of change between character states, ER, or for‐ and backward rates between states can take different values, ARD) indicated a slightly better performance of the more parameterized model (AIC of 106.9 for ER; AIC of 103.1 for ARD), with a fitted value of *Q* from low → high of 0.030 and low ← high of 0.910.

**Table 2:** Traits measured in the three growth environments (regular frost, mild, regular heat) for which the best supported evolutionary model was Ornstein‐Uhlenbeck with two optima (OUM), and the suggested trait optima (*θ*) for low‐ and high‐elevation species

	Treatment								
Trait	Frost			Mild			Heat		
	Best model	*θ*_LOW_	*θ*_HIGH_	Best model	*θ*_LOW_	*θ*_HIGH_	Best model	*θ*_LOW_	*θ*_HIGH_
Seed size				.					
Time to germination				.					
Growth									
Initial growth rate	.			.			.		
**Maximal growth rate**	.			OUM_–4.12_	0.21	0.18	.		
(–)Time to fastest growth	.			.			.		
**Asymptotic size**	“OUM”_–1.88_	57.15	30.44	“OUM”_1.84_	64.85	38.72	OUM_–2.36_	70.16	23.65
Number of leaves _S2_	.			.			.		
Leaf traits									
Leaf area	.			.			.		
**Specific leaf area**	.			“OUM”_1.35_	22.84	25.44	“OUM”_0.71_	25.16	28.87
**Leaf dry matter content**	OUM_–10.24_	20.64	16.83	.			.		
Leaf thickness _S2_	.			.			.		
Leaf dissection index	.			.			.		
Thermal tolerance s.l.									
Frost resistance									
*…acclimated (1 h at –6°C)*_S1_	.								
*…nonacclimated (1 h at –5°C)*_S2_				.					
*…acclimated (1 h at –11°C)*	.								
*…nonacclimated (1 h at –10°C)*_S2_				.					
**Heat resistance**									
*…acclimated (1 h at +47°C)*_S1_							.		
*…nonacclimated (1 h at +45°C)*_S2_				.					
***…acclimated (1 h at +51°C)***							“OUM”_–1.58_	13.16	21.42
***…nonacclimated (1 h at +50°C)*_S2_**				“OUM”_–0.84_	11.68	6.62			
Tolerance IGR	.						.		
**Tolerance MGR**	.						“OUM”_–0.80_	1.52	2.44
**Tolerance (–)XMID**	“OUM”_1.94_	0.02	0.04				“OUM”_–1.34_	0.00	0.11
**Tolerance ASYM**	“OUM”_–0.30_	–0.06	–0.14				OUM_–9.91_	0.78	–0.30

The table shows when OUM was the best or among the best models for each trait‐treatment combination based on the consensus of 100 simulations on the full phylogeny (full details in Table S3). When OUM was among the best by |ΔAICc| ≤ 2 to other best models, it is indicated by brackets (“OUM”); when it was not among the best supported models, it is indicated by a dot (.). Values in subscript are ΔAICc compared to the next best model (–; next model has higher AICc) or best model (+). If not specified, a trait was assessed both in sowing round S1 and in S2.

Several of the traits that differed between low‐ and high‐elevation species in mixed‐model analyses were confirmed to support a scenario of adaptive evolution with two optima. These traits included maximal growth rate, asymptotic size, leaf dry matter content, heat resistance, and tolerances in growth parameters (traits for which OUM had lowest AICc, although not always with ΔAICc < –2 compared to the next best model; Table 2). The optimum for high‐elevation species was at a lower MGR under control conditions, at a smaller asymptotic size under regular frost and heat, and at a lower LDMC under regular frost. Furthermore, high‐elevation species had an optimum at lower heat resistance when raised under mild conditions, but at higher heat resistance when raised under regular heat. Finally, high‐elevation species had optima at higher tolerance values to heat based on MGR and (–)XMID; they had been under selection for increased speed of growth in response to heat. But they had optima for tolerance to frost and heat based on asymptotic size that were lower. These results appear to be robust, as they did not deviate from the results obtained from bootstrap simulations (Table S3, Fig. S3).

Simulations performed on the phylogeny but with synthetic data (Fig. S4) revealed that adaptive divergence between low‐ and high‐elevation species was identified correctly when variance of trait estimates of species was low (standard error < 100, e.g., equaling a coefficient of variation of 5.6 for simulated data with BM1, *θ* = 1 and *σ* = 1) and the difference between optima (*θ*_LOW_, *θ*_HIGH_) large. False positives for the adaptive model were rare, whereas false negatives in favor of OU1 or WN were frequent. Analyses done after the random removal of a third of the species generally resulted in slightly increased support for OUM (Table S3, Fig. S3).

Measures of phylogenetic half‐life (i.e., ln(2)alpha^‐1^) were significantly larger than 0 for some traits (25%–38% depending on treatment, Table 3). The most constrained traits were associated with size and morphology, for example, ASYM with a half‐life of 11–15 mya (million years ago), LA_Heat_ with 25 mya, LTh with 7–10 mya, and LDI_Frost_ with 15 mya. Mixed‐model analysis with bootstrap simulations revealed that the evolution of trait values under regular frost was less constrained compared to mild conditions or regular heat (i.e., Frost vs. Mild: –0.605 [limits of 90% highest density interval: –0.616, –0.593]; Heat vs. Mild: 0.192 [0.180, 0.204]), resulting in a reduction of average half‐life of about 50% (Fig. S5).

**Table 3:** Half‐life of trait evolution toward the optimum (in mya)

	Treatment		
Trait	Frost	Mild	Heat
	*t*_½_ ± SD	*t*_½_ ± SD	*t*_½_ ± SD
Seed size		33.25 ± 28.42	
Time to germination		11.85 ± 11.41	
Growth			
Initial growth rate	5.40 ± 2.77^*^	6.51 ± 2.34^*^	6.66 ± 2.06 ^*^
Maximal growth rate	0.75 ± 2.74	0.14 × 10^4^ ± 9.46 × 10^4^	25.75 ± 667.31
(–)Time to fastest growth	1.21 × 10^4^ ± 70.84 × 10^4^	0.55 × 10^4^ ± 30.15 × 10^4^	2.9 × 10^4^ ± 142.2 × 10^4^
Asymptotic size	10.90 ± 4.99^*^	15.49 ± 6.88^*^	14.31 ± 9.51
Number of leaves _S2_	2.58 ± 2.58	3.53 ± 3.36	3.52 ± 2.87
Leaf traits			
Leaf area	12.09 ± 12.09	14.23 ± 11.59	25.49 ± 11.98^*^
Specific leaf area	7.48 ± 2.33^*^	5.27 ± 1.78^*^	5.25 ± 1.94^*^
Leaf dry matter content	2.62 ± 3.19	2.86 ± 2.71	349.66 ± 2.51 × 10^4^
Leaf thickness _S2_	9.55 ± 2.56^*^	7.33 ± 1.57^*^	7.81 ± 1.29 ^*^
Leaf dissection index	15.07 ± 6.87^*^	23.36 ± 16.50	11.44 ± 8.89
Thermal tolerance s.l.			
Frost resistance			
*…acclimated (1 h at –6°C)*_S1_	0.75 ± 1.49		
*…nonacclimated (1 h at –5°C)*_S2_		6.24 ± 4.47	
*…acclimated (1 h at –11°C)*	3.43 ± 2.70		
*…nonacclimated (1 h at –10°C)*_S2_		1.74 ± 2.38	
Heat resistance			
*…acclimated (1 h at +47°C)*_S1_			2.14 ± 2.54
*…nonacclimated (1 h at +45°C)*_S2_		4.15 ± 3.32	
*…acclimated (1 h at +51°C)*			1.69 ± 1.77
*…nonacclimated (1 h at +50°C)*_S2_		24.86 × 10^4^ ± 475.96 × 10^4^	
Tolerance IGR	5.74 ± 2.47^*^		5.51 ± 1.75 ^*^
Tolerance MGR	0.16 ± 0.26		0.88 ± 0.70
Tolerance (–)XMID	0.16 ± 0.39		1.46 ± 0.81^*^
Tolerance ASYM	0.09 ± 0.15		0.71 ± 0.49

Values of phylogenetic half‐life (under OU1, or OUM when it was the best model or among the best models by |ΔAICc| ≤ 2 to other such models) are based on ARD models and 100 independent stochastic character maps (full details in Table S3). Values are means ± SD of phylogenetic half‐life in mya for traits within treatments, calculated based on bootstrap replicates (i.e., the random removal of a third of the species, with *N* = 10,000 simulations per trait within environment). Significance in half‐life (^*^) was calculated by (mean – 1.64SD) > 0. If not specified, a trait was assessed both in sowing round S1 and in S2.

## MULTITRAIT RELATIONSHIPS AND TRADE‐OFFS

A principal component analysis on trait values of trait‐growth treatment combinations revealed their correlation structure (Fig. S6 in Supporting Information SI5). The first PC axis explained 15.7% of the total variance and depicted the relationship between timing of plant growth, especially in the heat treatment, and plant size (under mild conditions). The second PC (10.5%) was primarily influenced by LTh, and to a lesser extent by basal resistance and tolerance components, portraying a complex distinction between these two strategies. The optimal number of principal components to retain (i.e., lowest mean squared error and highest mean success) based on cross validation was 35 (accounting for 99% of trait variation; Fig. S7).

**Figure 2:**
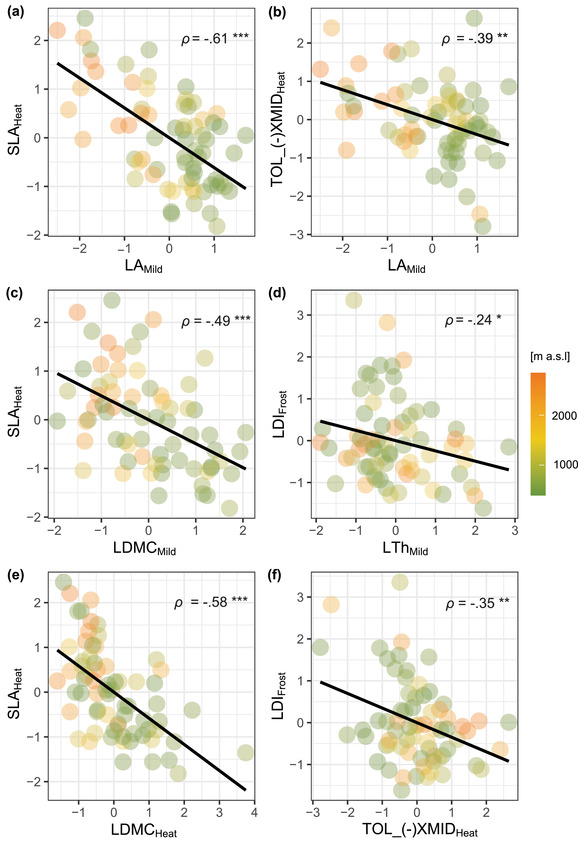
Trait differentiation between low‐ and high‐elevation species, as revealed by discriminant analyses and multitrait correlations. Each point represents a species. The median elevation of origin is represented by a color scale ranging from green (low elevation) to brown (high elevation). The black line reflects the relationship between pairs of traits, and the associated correlation coefficient is reported (significance: ^*^ *P* < 0.05, ^**^ *P* < 0.01, ^***^ *P* < 0.001; full details in Table S4). Trait values are centered and scaled to unit variance.

With these PCs, taxa could be assigned to their elevation of origin, either low or high, with an accuracy of 98% and 94.4%, respectively (Fig. S7). In multivariate space, the trait with the greatest weight was leaf area under mild conditions, whereas the other traits that contributed most to differentiating low‐ and high‐elevation species represented leaf morphology under mild, frost, and warm conditions (i.e., LTh, LDMC, SLA), speed of growth under heat (i.e., TOL_IGR, TOL_[–]XMID), and leaf shape and tolerance under frost (i.e., LDI, TOL_ASYM; Fig. S8). Pearson correlations were significantly negative between specific leaf area under heat and leaf area (LA_Mild_; Fig. 2a) or leaf dry matter content (LDMC_Mild_, Fig. 2c; LDMC_Heat_, Figs. 2e, S9, and S10 and Table S4), with especially the latter correlation being likely driven by nonindependence of calculating estimates. Furthermore, leaf area under mild conditions was negatively correlated with heat tolerance based on the midpoint of growth (TOL_[–]XMID; Fig. 2b), suggesting a trade‐off between maintaining large size and speeding up growth under heat. Tolerance under regular heat based on the midpoint of growth was also negatively associated with leaf dissection index under frost (Fig. 2f), which in turn was negatively associated with leaf thickness under mild conditions (Fig. 2d). However, these two correlations involved some traits not consistently linked to elevational distribution (based on mixed models and evolutionary models).

## Discussion

Past studies in ecology and biogeography have indicated that temperature is a limiting factor of species distribution, suggesting that there are ubiquitous constraints to the evolution of the climate niche. To improve our understanding of such constraints, we studied nearly 100 plant species differing in elevational distribution and presumably with different climate niches. More specifically, we investigated which traits differed with elevational distribution, whether those traits had been under divergent selection over the elevational gradient, and potential sources of constraints in their adaptive divergence. The species were found to systematically differ in few traits. Most importantly, higher elevation plants were found to have smaller and less robust leaves. Further differences emerged when growing conditions included regular heat bouts. Then higher elevation species accelerated growth more, at the cost of a considerable reduction in size. The same or similar traits were found to be under divergent selection over the elevational gradient, and some were involved in moderate trade‐offs, notably the ability to speed up growth under heat and plant size. The discussion focuses on traits under divergent selection, evidence for evolutionary constraints, and hypotheses on the selection environment and adaptive strategies.

### TRAIT DIFFERENCES BETWEEN LOW‐ AND HIGH‐ELEVATION SPECIES

Generalized linear models and evolutionary models mainly overlapped in pointing to differences in traits depending on whether species had low‐ or high‐elevation distributions (Table 4). The traits that were consistently different between low‐ and high‐elevation species in the two types of models included plant size (ASYM), leaf morphology (LDMC), the response of speed of growth to stress, and thermal resistance.

**Table 4:** Summary of results on trait differences between low‐ and high‐elevation species in the three growth treatments (regular frost [F], mild [M], regular heat [H]) across types of analyses (mixed models [brm], testing for two evolutionary optima [OUM], half‐life of trait evolution, discriminant analysis of principal components [DAPC], and [negative] correlations [*ρ*])

Trait	Effect of elevation							Phylogenetic inertia			Trade‐off					
	Brm				OUM			Half‐life			DAPC			*ρ*		
	Elev	F	M	H	F	M	H	F	M	H	F	M	H	F	M	H
Seed size		.		.	.		.	.		.	.		.	.		.
Time to germination		.		.	.		.	.		.	.		.	.		.
Growth																
Initial growth rate			c					x	x	x						
Maximal growth rate			c	x+		x–										
(–)Time to fastest growth			c	x+												
Asymptotic size			c	x–	x–	x–	x–	x	x		_		_			
Number of leaves _S2_			c									_	_			
Leaf traits																
**Leaf area**	**x–**		**c**	**x–**						**x**	_	**x**	_		**x**	
**Specific leaf area**			**c**			**x+**	**x+**	**x**	**x**	**x**	_		**x**			**x**
**Leaf dry matter content**	**x–**		**c**		**x–**							**x**	**x**		**x**	**x**
Leaf thickness _S2_			c					x	x	x		x			x	
**Leaf dissection index**			**c**	**x–**				**x**			**x**	_	_	**x**		
Thermal tolerance s.l.																
Frost resistance																
*…acclimated (1 h at –6°C)*_S1_			.	.		.	.		.	.						
*…nonacclimated (1 h at –5°C)*_S2_		.		.	.		.	.		.						
*…acclimated (1 h at –11°C)*			.	.		.	.		.	.						
*…nonacclimated (1 h at –10°C)*_S2_		.		.	.		.	.		.						
Heat resistance																
*…acclimated (1 h at +47°C)*_S1_		.	.		.	.		.	.							
*…nonacclimated (1 h at +45°C)*_S2_		.		.	.		.	.		.						
*…acclimated (1 h at +51°C)*		.	.		.	.	x+	.	.							
*…nonacclimated (1 h at +50°C)*_S2_	x–	.		.	.	x–	.	.		.						
Tolerance IGR		c	.			.		x	.	x	_	.	x		.	
Tolerance MGR		c	.	x+		.	x+		.			.			.	
**Tolerance (–)XMID**		**c**	.	**x+**	**x+**	.	**x+**		.			.	**x**		.	**x**
Tolerance ASYM		c	.	x–	x–	.	x–		.		x	.			.	

Brm: An "x" indicates that a significant effect of elevation (Elev) or an interaction between elevation and growth environment was found; the sign represents the direction of change relative to the contrast (c) environment. OUM: The "x" indicates that OUM was the best or among the best supported evolutionary models; the sign shows whether the optimum for high‐ compared to low‐elevation species was lower (negative) or higher (positive). Phylogenetic inertia: The "x" represents that the half‐life of trait evolution was >5 mya (half‐life under OU1, or OUM when it was the best model or among the best models). Trade‐off: The "x" indicates that a trait contributed considerably to the loading in DAPC and was involved in significant (negative) correlations (*ρ*). Additional signs indicate a trait was not assessed in a particular environment (.), or was excluded from analysis (_). Lines in bold highlight traits that showed a significant effect of elevation and were involved in negative relationships with others. If not specified, a trait was assessed both in sowing round S1 and in S2.

Across growth environments, alpine species had smaller leaves and less dry matter content in leaves (Figs. S2h and 1b). Evolutionary models supported that optima for plant size were at smaller values for high‐compared to low‐elevation species under all growth conditions. Furthermore, they supported an optimum at lower LDMC under growth conditions with regular frost, and as a trend an optimum at higher SLA, which is typically inversely related to LDMC, under mild conditions or conditions with regular heat. Results for size are in line with previous studies on multispecies comparisons, which reported a reduction in leaf size with increasing elevational distribution (Qi et al. 2014; Zhong et al. 2014). In contrast, previous studies reported either higher LDMC and smaller SLA (Körner et al. 1986; Qi et al. 2014; Rosbakh et al. 2014; Midolo et al. 2019) or the contrary (Zhong et al. 2014). Lower LDMC and higher SLA as found in our study are typically associated with a strategy of fast assimilation and growth but weak hardiness and short leaf life span (Pérez‐Harguindeguy et al. 2013).

The other type of trait that generally differed between low‐ and high‐elevation species was the response to heat during the growth phase. Both regular heat and frost caused plants and their leaves to be smaller, indicating that conditions were generally stressful (Table 1). Furthermore, plants speeded up growth under these conditions; the time to reach the midpoint of asymptotic size was shorter ([–]XMID), and under the regular occurrence of heat bouts, also the maximum growth rate was higher (MGR). An important finding of this study is that higher compared to lower elevation species could accelerate growth under conditions with regular heat bouts (MGR, [–]XMID; Fig. 1d,e), at the cost that their leaves were smaller (Fig. 1a,f). Evolutionary models too provided evidence that tolerance for speeding up growth (TOL_MGR, TOL_[–]XMID) under heat had an optimum at higher values in high‐elevation species. Evolutionary models pointed also to an optimum at higher values for tolerance of speeding up growth under frost (as a trend). Results suggest general selection for escape strategies under stress, and that high‐elevation species seem to have adapted to exploit heat phases better by growing faster. The finding is novel and needs verification in more plant families.

Interestingly, low‐ and high‐elevation species also differed in thermal resistance, although not in the direction that was previously advocated. Mixed‐model analysis supported that heat resistance without prior acclimation decreased with median elevational of species distribution (Table 4). Evolutionary models supported a lower optimum for basal heat resistance in high‐elevation species, but a higher optimum of acclimation‐based heat resistance. However, increased frost resistance (after acclimation) in high‐elevation species was only detected in the first round of sowing but not the second (Fig. S2r), and the result was significant only when the phylogeny was not considered (model 15 in SI3). In contrast, a number of earlier studies documented rather consistently that high‐elevation tree species were more frost resistant (Körner 2003; Taschler and Neuner 2004; Neuner 2014; Neuner et al. 2020; Schrieber et al. 2020). The discrepancy may have two potential reasons. First, the latter studies did not account for phylogeny in their analysis, which could have produced increased type I error (Li and Ives 2017). Second, there may be fundamental differences between trees and herbaceous plants in the role of frost resistance on distribution limits because of differences in the life history or the plant architecture and functioning.

As a side note, our study demonstrated environment dependence in the detection of traits under selection. Evidence for divergent adaptation between low‐ and high‐elevation species was more common for traits recorded under warmer, regular‐heat conditions compared to the regular occurrence of frost (Table 4). This insight warrants attention in evolutionary trait modeling in a comparative context. Comparative studies typically rely on trait measures taken in the field or on collection material (e.g., Luxbacher and Knouft 2009; Edwards and Smith 2010), or after raising organisms under standard conditions (e.g., Kellermann et al. 2012; Mason and Donovan 2015). While the former brings the problem of the inability of separating the effects of genetics and the environment on trait differences, the latter has the flaw that the adaptation potential of a trait may not be detected as the environment is not the one in which divergence is expressed. For Brassicaceae along the elevational gradient, it is warmer conditions that seem to have played a stronger role in adaptive divergence.

In summary, the picture that emerges is that high‐ compared to low‐elevation species of Brassicaceae are fast growers when it is warm, have reduced size and less hardy leaves, and they are neither particularly frost nor heat resistant.

### EVOLUTIONARY INERTIA

Considerable evolutionary half‐lives of traits associated with disparate elevational distribution were found (Table 4). The highest value of phylogenetic inertia was found for leaf area, one of the two most discriminating traits between low‐ and high‐elevation species (Fig. S8). The half‐life was estimated to be ∼25 mya when leaf area was expressed under the regular occurrence of heat (Table 3). Also, asymptotic size and leaf dissection index (under regular occurrence of frost) had considerable half‐lives, between 11 and 15 mya. The remaining traits with significant half‐lives (i.e., IGR, SLA, LTh, TOL_IGR, and TOL_[–]XMID) had lower, but still considerable values ranging from ∼1.5 mya for heat tolerance based on the time until fastest growth, to 9.5 mya for leaf thickness under cold conditions.

Furthermore, phylogenetic inertia of traits was found to depend on the environment in which they were expressed (Fig. S5b,d). The heat treatment was not only the more discriminating among low‐ and high‐elevation species, that and the mild treatment led to trait expression associated with longer phylogenetic half‐lives. The half‐life of traits expressed under mild and heat was 50% longer compared to traits expressed under the regular occurrence of frost. Results therefore suggest that adaptation to exploit or live under generally warmer conditions is more constrained. The result is in line with a recent large‐scale phylogenetic analysis, showing that across plants and animals, the rate of adaptation to warm conditions was much slower than to cold, both in endotherms and ectotherms (Bennet et al. 2021).

In summary, for traits distinguishing high‐ from low‐elevation species, considerable half‐lives indicated constraints to adaptive evolution. Furthermore, under environmental conditions these traits were most divergent, under regular heat, adaptive evolution lagged behind farthest.

### TRADE‐OFFS

We detected trade‐offs among traits that contributed most to the differentiation between low‐ and high‐elevation species, which may explain the evolutionary inertia discussed above (Table 4; Fig. S9). Specific leaf area under heat was negatively related with leaf dry matter content on the one hand (Fig. 2c,e), and with leaf area under mild conditions on the other hand (Fig. 2a). In turn, leaf area under mild conditions was negatively correlated with heat tolerance based on the midpoint of growth (TOL_[–]XMID; Fig. 2b). The phenotypic aspects that these traits represent are probably larger, as highly correlated traits (assessed in particular treatments) were removed before analysis. Based on this reasoning, we may generalize that an important trade‐off was between assimilation‐efficient but not very hardy leaves and plant size. Another was between size and the capacity to speed up growth under heat. In other words, there is good macroevolutionary evidence that assimilation‐potent leaves with little dry mass, fast plant growth under heat, and small size come as a syndrome shaped by trade‐offs that generally distinguishes high‐ from low‐elevation species. Beyond, weak to moderate negative relationships were detected between nonacclimated resistances (to cold or heat) and assimilatory capacity (SLA, number of leaves; SI5). But, resistance did not figure among the nine most relevant traits in differentiating low‐ and high‐elevation species (Table 4; Fig. S8).

The trade‐off complex involving weak leaf morphology, fast growth under heat, and reduced plant size is in high accordance with universal constraints described for plant functioning and life‐history evolution. According to the world‐wide leaf economics spectrum (Wright et al. 2004), species either follow a strategy of quick return on investment, with nutrient‐rich leaves, high photosynthetic rates, and short life spans or a strategy of slow return, with expensive but long‐lived leaves. In a broader context, the continuum of fast production versus slowness is also reflected in the concept of r/K selection (Pianka 1970), where r‐selected species grow more rapidly, but to a smaller size and they reproduce earlier, whereas K‐selected species grow more slowly, but to larger size and they reproduce later. For plants, the concept was expanded, with now three strategies—ruderal (R), stress‐tolerant (S), and competitive (C)—being positioned along three axes of environmental gradients: disturbance, abiotic stress, and competition (Grime 1977). Pierce et al. (2013) showed how these strategies can be correctly attributed with the use of the same leaf traits that showed the main trade‐offs in our work, that is, leaf area, leaf dry matter content, and specific leaf area. However, and in contrary to their reports, LDMC and LA did not form separate axes in our study. Nonetheless, following their sorting suggests that alpine (Brassicaceae) species primarily follow a ruderal (or r) strategy, whereas lowland species follow an S/C (or K) strategy.

Several insights speak in favor that the environmental driver of selection under high‐elevation conditions is the short growing season. On the one hand, our study showed that plants of high elevations were not better at coping with cold, but they had evolved to better exploit warm conditions for fast growth. In line, previous ecophysiological studies reported higher photosynthetic rate in alpine herbaceous species cultivated at warmer temperature (Mächler an Nösberger 1977) or during daily warm spells in the wild (Körner and Diemer 1987), pointing to faster resource acquisition under warm conditions. On the other hand, niche modeling suggested that upper range limits were constrained not primarily by the direct effect of cool temperatures but the brevity of the growing season (Morin et al. 2007; Patsiou et al. 2021). These studies too pointed to speed of growth or development being under selection under higher elevation conditions. Based on the two sets of insights, we propose that whether a species (of Brassicaceae) can live at high elevation depends on the ability to cope with the short growing season, which is achieved by maximizing growth during short thermal windows when the temperature is relatively high. Superficially, the geographic pattern may resemble counter‐gradient variation (Conover and Schultz 1995), where high‐elevation genotypes grow faster, whereas their environment may generally cause growth to be slow. One distinction is that the acceleration of growth is expressed only under warmer conditions, and a second is that the relevant environmental difference may be the shorter growing season.

Insights evoke novel hypotheses on the causes of range limits and the evolution of the climate niche: The short growing season of higher elevations imposes selection in favor of fast growth and leaves optimized for high photosynthetic activity, and plants respond to selection in these traits by exploiting heat phases of the day. Generally warmer conditions at lower elevations impose selection for higher basal heat resistance and leaf endurance. The mentioned traits are involved in allocation and/or genetic trade‐offs between each other and with plant size. The combination of multivariate selection, nonindependence among traits, and generally warm conditions imposing added evolutionary inertia may make climate niche evolution very slow and come to a seeming halt at range limits, producing a pattern of disparate elevational distribution among species.

## Conclusion

Our study highlights that the most discriminating traits separating high‐ from low‐elevation Brassicaceae species are their ability to speed up growth under conditions with heat bouts, at the cost of reduced leaf and plant size, and possibly a more ephemeral lifestyle with less investment into leaves and lower basal heat resistance. Results suggest a general trade‐off between exploiting the short vegetation period at high elevation and being less enduring in general or under certain thermal extremes or under competition. The trade‐off could be a result of multivariate selection differing among low‐ and high‐elevation sites and/or nonindependence of trait expression. In parallel, we found that especially frost resistance did not play a role in differentiating species along the elevational gradient. Finally, we found that signatures of divergent adaptation were more commonly detected under conditions with regular heat compared to mild or regular‐frost conditions, and that adaptation under such warm conditions was more constrained.

## AUTHOR CONTRIBUTIONS

AM and YW conceived the study and conducted the field work. AM executed the experimental work, run the statistical analyses, and wrote the first draft of the manuscript. YW contributed to writing. All authors gave final approval for publication.

## CONFLICT OF INTEREST

The authors declare no conflict of interest.

## DATA ARCHIVING

All relevant data are within this article and its Supporting Information. Traits values are deposited at try‐db.org.

Associate Editor: T. Chapman

Handling Editor: M. Zelditch

## Supporting information

SI1 – List of species and populations used in this study

SI2 – Trait assessment and methods for validating evolutionary models (with Fig. S1)

SI3 – Results of mixed models (with Tables S1, S2, and Fig. S2)

SI4 – Results of evolutionary models (including simulations; with Table S3 and Figs. S3 to S5)

SI5 – Multivariate analysis, and Pearson's correlations (with Table S4 and Figs. S6 to S10)
