# Supplementary material for: Trait divergence and trade‐offs among Brassicaceae species differing in elevational distribution
Source: Evolution. 2022 Jul 20;76(9):1986–2003. doi: 10.1111/evo.14554 (PMC9545065; doi:10.1111/evo.14554)
Supplement: Supplementary file 3 — SI3 – Results of mixed models (with Tables S1, S2, and Fig. S2) [file EVO-76-1986-s004.docx]

Supporting information SI3 – Results of mixed models

**Table S1.** Results of mixed models. Traits that showed a significant effect of elevation (continuous variable) or an interaction effect of elevation-by-treatment are written in red. Effects and their model estimates are highlighted in yellow if there were differences between the model accounting for phylogeny (left) and the model that did not (right). Significant fixed effects are written in bold (95% equal-tailed interval not overlapping with 0). Posterior medians of fixed effects are reported, relative to the baseline of average elevation or mild growth conditions and, for tolerance traits, relative to estimates under frost. For random effects, reporting includes the within-group variance (σ^2^), the between-group variance (τ_00_), and the number of levels, N (i.e., species and/or round of sowing). Models were run on XMID and TOL_XMID.

| **1. Bayesian mixed-effects model for: SSIZ** | | | | |
| --- | --- | --- | --- | --- |
| *Predictors* | *Median* | *CI (95%)* | *Median* | *CI (95%)* |
| **Intercept** | **0.9933** | **0.1471 – 1.8284** | **0.3614** | **0.0911 – 0.6306** |
| Elevation | 0.0583 | -0.1659 – 0.2802 | -0.1134 | -0.3844 – 0.1520 |
| **Random effects** | | | |  |
| σ2 | 1795.1641 | | |  |
| τ00 | 1.1661 |  |  |  |
| N | 95 taxa |  |  |  |
| Observations | 95 |  | 95 |  |

| **2. Bayesian mixed-effects model for: TGER** | | | | | | | | |  |
| --- | --- | --- | --- | --- | --- | --- | --- | --- | --- |
| *Predictors* | *Median* | *CI (95%)* | | | *Median* | *CI (95%)* | | |  |
| **Intercept** | **2.5841** | **1.6294 – 3.1756** | | | **2.4456** | **0.8774 – 3.2305** | | |  |
| Elevation | 0.0426 | -0.0322 – 0.1142 | | | 0.0547 | -0.0060 – 0.1157 | | |  |
| **Random effects** | | | | | | | | |  |
| σ2 | 0.3580 |  | | | 0.3989 |  | | |  |
| τ00 | 0.2033 sowing_id | | | | 0.5075 sowing_id | | | |  |
| 0.0133 taxa | | | | | | | | |  |
| N | 93 taxa |  | | | 2 sowing_id |  | | |  |
| 2 sowing_id | | | | | | | | |  |
| Observations | 168 |  | | | 168 |  | | |  |
| **3. Bayesian mixed-effects model for: IGR** | | | | | | | | | |
| *Predictors* | | | *Median* | *CI (95%)* | | | *Median* | *CI (95%)* | |
| Intercept | | | -0.5728 | -1.5335 – 0.1254 | | | -0.6115 | -1.9583 – 0.4005 | |
| Elevation | | | -0.0088 | -0.0325 – 0.0143 | | | -0.0090 | -0.0316 – 0.0143 | |
| Treatment (Frost vs Mild) | | | -0.0061 | -0.0377 – 0.0250 | | | -0.0062 | -0.0387 – 0.0265 | |
| Treatment (Heat vs Mild) | | | -0.0190 | -0.0513 – 0.0125 | | | -0.0191 | -0.0505 – 0.0140 | |
| Elevation x Frost treatment vs Mild | | | 0.0124 | -0.0185 – 0.0442 | | | 0.0125 | -0.0202 – 0.0449 | |
| Elevation x Heat treatment vs Mild | | | 0.0149 | -0.0172 – 0.0468 | | | 0.0154 | -0.0169 – 0.0476 | |
| **Random effects** | | |  |  | | |  |  | |
| σ2 | | | 0.0004 |  | | | 0.0006 |  | |
| τ00 | | | 0.0039 |  | | | 0.0036 |  | |
| N | | | 90 taxa |  | | | 1 sowing_id |  | |
|  | | | 1 sowing_id |  | | |  |  | |
| Observations | | | 263 |  | | | 263 |  | |

| **4. Bayesian mixed-effects model for: MGR** | | | | |
| --- | --- | --- | --- | --- |
| *Predictors* | *Median* | *CI (95%)* | *Median* | *CI (95%)* |
| **Intercept** | **-1.5334** | **-2.3955 – -0.6486** | **-1.5233** | **-2.4030 – -0.6429** |
| Elevation | -0.0296 | -0.0989 – 0.0392 | -0.0351 | -0.1026 – 0.0328 |
| Treatment (Frost vs Mild) | -0.0409 | -0.1394 – 0.0540 | -0.0419 | -0.1401 – 0.0556 |
| **Treatment (Heat vs Mild)** | **0.4227** | **0.3233 – 0.5221** | **0.4206** | **0.3201 – 0.5214** |
| Elevation x Frost treatment vs Mild | 0.0129 | -0.0801 – 0.1071 | 0.0123 | -0.0825 – 0.1084 |
| **Elevation x Heat treatment vs Mild** | **0.1292** | **0.0312 – 0.2240** | **0.1282** | **0.0331 – 0.2251** |
| **Random effects** |  |  |  |  |
| σ2 | 0.0086 |  | 0.0076 |  |
| τ00 | 0.0312 |  | 0.0318 |  |
| N | 92 taxa |  | 2 sowing_id |  |
|  | 2 sowing_id |  |  |  |
| Observations | 467 |  | 467 |  |

| **5. Bayesian mixed-effects model for: XMID** | | | | |
| --- | --- | --- | --- | --- |
| *Predictors* | *Median* | *CI (95%)* | *Median* | *CI (95%)* |
| **Intercept** | **3.1455** | **1.8422 – 3.7487** | **3.1763** | **1.9100 – 3.7803** |
| Elevation | 0.0189 | -0.0133 – 0.0514 | 0.0189 | -0.0080 – 0.0469 |
| Treatment (Frost vs Mild) | -0.0330 | -0.0684 – 0.0020 | -0.0332 | -0.0738 – 0.0050 |
| **Treatment (Heat vs Mild)** | **-0.1146** | **-0.1502 – -0.0785** | **-0.1131** | **-0.1534 – -0.0741** |
| Elevation x Frost treatment vs Mild | -0.0040 | -0.0393 – 0.0314 | -0.0053 | -0.0441 – 0.0340 |
| **Elevation x Heat treatment vs Mild** | **-0.0650** | **-0.1015 – -0.0282** | **-0.0641** | **-0.1042 – -0.0220** |
| **Random effects** |  |  |  |  |
| σ2 | 29.3880 |  | 24.1119 |  |
| τ00 | 14.7235 |  | 19.9767 |  |
| N | 92 taxa |  | 2 sowing_id |  |
|  | 2 sowing_id |  |  |  |
| Observations | 460 |  | 460 |  |

| **6. Bayesian mixed-effects model for: ASYM** | | | | |
| --- | --- | --- | --- | --- |
| *Predictors* | *Median* | *CI (95%)* | *Median* | *CI (95%)* |
| **Intercept** | **3.8519** | **3.4457 – 4.2379** | **3.8260** | **3.5206 – 4.1236** |
| **Elevation** | -0.0403 | -0.1234 – 0.0472 | **-0.2531** | **-0.3296 – -0.1783** |
| **Treatment (Frost vs Mild)** | **-0.1394** | **-0.1973 – -0.0845** | **-0.1383** | **-0.2511 – -0.0318** |
| **Treatment (Heat vs Mild)** | **-0.1526** | **-0.2118 – -0.0956** | **-0.1338** | **-0.2387 – -0.0257** |
| Elevation x Frost treatment vs Mild | -0.0256 | -0.0822 – 0.0314 | -0.0304 | -0.1393 – 0.0760 |
| **Elevation x Heat treatment vs Mild** | **-0.1349** | **-0.1931 – -0.0757** | **-0.1344** | **-0.2444 – -0.0248** |
| **Random effects** |  |  |  |  |
| σ2 | 597.2925 |  | 3.6393 |  |
| τ00 | 166.9919 |  | 1007.9378 | |
| N | 92 taxa |  | 2 sowing_id |  |
|  | 2 sowing_id |  |  |  |
| Observations | 460 |  | 460 |  |

| **7. Bayesian mixed-effects model for: NLEA** | | | | |
| --- | --- | --- | --- | --- |
| *Predictors* | *Median* | *CI (95%)* | *Median* | *CI (95%)* |
| **Intercept** | **2.7193** | **1.9778 – 3.6339** | **2.7541** | **2.0872 – 3.7375** |
| Elevation | -0.0107 | -0.1272 – 0.1028 | 0.0287 | -0.0940 – 0.1511 |
| **Treatment (Frost vs Mild)** | **-0.0642** | **-0.1226 – -0.0054** | -0.0581 | -0.2304 – 0.1085 |
| Treatment (Heat vs Mild) | 0.0047 | -0.0544 – 0.0638 | 0.0137 | -0.1619 – 0.1813 |
| Elevation x Frost treatment vs Mild | -0.0089 | -0.0660 – 0.0498 | -0.0000 | -0.1678 – 0.1669 |
| Elevation x Heat treatment vs Mild | -0.0247 | -0.0815 – 0.0334 | -0.0179 | -0.1898 – 0.1539 |
| **Random effects** |  |  |  |  |
| σ2 | 102.8772 |  | -61.7739 |  |
| τ00 | 10.2023 |  | 150.9636 |  |
| N | 89 taxa |  | 1 sowing_id |  |
|  | 1 sowing_id |  |  |  |
| Observations | 257 |  | 257 |  |

**8. Bayesian mixed-effects model for: LA**

| *Predictors* | *Median* | *CI (95%)* | *Median* | *CI (95%)* |
| --- | --- | --- | --- | --- |
| **Intercept** | **3.5008** | **2.4944 – 4.5804** | **3.4708** | **2.4693 – 4.5139** |
| **Elevation** | **-0.3482** | **-0.5105 – -0.1898** | **-0.6721** | **-0.8112 – -0.5295** |
| **Treatment (Frost vs Mild)** | **-0.1618** | **-0.2617 – -0.0619** | -0.1320 | -0.3331 – 0.0668 |
| **Treatment (Heat vs Mild)** | -0.2548 | -0.3541 – -0.1543 | **-0.2125** | **-0.4113 – -0.0073** |
| Elevation x Frost treatment vs Mild | 0.0325 | -0.0713 – 0.1322 | 0.0257 | -0.1805 – 0.2276 |
| **Elevation x Heat treatment vs Mild** | **-0.1171** | **-0.2216 – -0.0125** | -0.1269 | -0.3312 – 0.0784 |
| **Random effects** |  | |  | |
| σ2 | 180945.8460 | | 334942.1002 | |
| τ00 | 395.7070 | 4761.9862 | | |
| N | 93 taxa | 2 sowing_id | | |
|  | 2 sowing_id |  | | |
| Observations | 434 | 434 | | |

| **9. Bayesian mixed-effects model for: SLA** | | | | |
| --- | --- | --- | --- | --- |
| *Predictors* | *Median* | *CI (95%)* | *Median* | *CI (95%)* |
| **Intercept** | **3.0456** | **2.8726 – 3.2001** | **3.0949** | **2.8723 – 3.2247** |
| Elevation | 0.0182 | -0.0474 – 0.0854 | 0.0511 | -0.0032 – 0.1033 |
| **Treatment (Frost vs Mild)** | **-0.1301** | **-0.1948 – -0.0641** | **-0.1262** | **-0.2035 – -0.0481** |
| **Treatment (Heat vs Mild)** | **0.1121** | **0.0457 – 0.1789** | **0.1151** | **0.0341 – 0.1941** |
| Elevation x Frost treatment vs Mild | -0.0295 | -0.0942 – 0.0359 | -0.0291 | -0.1044 – 0.0498 |
| Elevation x Heat treatment vs Mild | 0.0175 | -0.0495 – 0.0842 | 0.0355 | -0.0435 – 0.1153 |
| **Random effects** |  |  |  |  |
| σ2 | 63.5872 |  | 20.6432 |  |
| τ00 | 53.3399 |  | 97.3756 |  |
| N | 93 taxa |  | 2 sowing_id |  |
|  | 2 sowing_id |  |  |  |
| Observations | 434 |  | 434 |  |

| **10. Bayesian mixed-effects model for: LDMC** | | | | |
| --- | --- | --- | --- | --- |
| *Predictors* | *Median* | *CI (95%)* | *Median* | *CI (95%)* |
| **Intercept** | **2.9769** | **2.8141 – 3.1553** | **2.9575** | **2.8152 – 3.1424** |
| **Elevation** | **-0.0665** | **-0.1148 – -0.0181** | **-0.0864** | **-0.1241 – -0.0486** |
| Treatment (Frost vs Mild) | 0.0419 | -0.0001 – 0.0833 | 0.0432 | -0.0120 – 0.0989 |
| **Treatment (Heat vs Mild)** | **-0.1818** | **-0.2242 – -0.1403** | **-0.1727** | **-0.2279 – -0.1180** |
| Elevation x Frost treatment vs Mild | 0.0083 | -0.0339 – 0.0502 | 0.0031 | -0.0524 – 0.0570 |
| Elevation x Heat treatment vs Mild | -0.0198 | -0.0624 – 0.0237 | -0.0276 | -0.0838 – 0.0277 |
| **Random effects** |  |  |  |  |
| σ2 | 11.6147 |  | -1.3698 |  |
| τ00 | 17.7749 |  | 30.5715 |  |
| N | 92 taxa |  | 2 sowing_id |  |
|  | 2 sowing_id |  |  |  |
| Observations | 423 |  | 423 |  |

| **11. Bayesian mixed-effects model for: LTh** | | | | |
| --- | --- | --- | --- | --- |
| *Predictors* | *Median* | *CI (95%)* | *Median* | *CI (95%)* |
| **Intercept** | **-1.8316** | **-2.4362 – -1.2884** | **-1.9051** | **-2.5051 – -1.3061** |
| Elevation | 0.0256 | -0.0225 – 0.0750 | 0.0141 | -0.0342 – 0.0610 |
| **Treatment (Frost vs Mild)** | **0.0770** | **0.0178 – 0.1346** | **0.0764** | **0.0094 – 0.1423** |
| Treatment (Heat vs Mild) | -0.0167 | -0.0730 – 0.0407 | -0.0194 | -0.0858 – 0.0463 |
| Elevation x Frost treatment vs Mild | -0.0108 | -0.0690 – 0.0464 | -0.0120 | -0.0774 – 0.0553 |
| Elevation x Heat treatment vs Mild | 0.0004 | -0.0586 – 0.0600 | 0.0021 | -0.0639 – 0.0699 |
| **Random effects** |  |  |  |  |
| σ2 | 0.0029 |  | 0.0027 |  |
| τ00 | 0.0011 |  | 0.0012 |  |
| N | 86 taxa |  | 1 sowing_id |  |
|  | 1 sowing_id |  |  |  |
| Observations | 241 |  | 241 |  |

**12. Bayesian mixed-effects model for: LDI**

| *Predictors* | *Median* | *CI (95%)* | *Median* | *CI (95%)* |
| --- | --- | --- | --- | --- |
| Intercept | 0.4964 | -0.1029 – 0.8456 | 0.5314 | -0.0641 – 0.8177 |
| Elevation | 0.0393 | -0.0058 – 0.0863 | -0.0206 | -0.0612 – 0.0204 |
| Treatment (Frost vs Mild) | -0.0097 | -0.0360 – 0.0169 | -0.0045 | -0.0632 – 0.0536 |
| Treatment (Heat vs Mild) | -0.0170 | -0.0437 – 0.0099 | -0.0278 | -0.0875 – 0.0324 |
| Elevation x Frost treatment vs Mild | 0.0188 | -0.0078 – 0.0455 | 0.0128 | -0.0448 – 0.0718 |
| **Elevation x Heat treatment vs Mild** | **-0.0451** | **-0.0724 – -0.0171** | -0.0525 | -0.1117 – 0.0075 |
| **Random effects** |  |  |  |  |
| σ2 | 0.2810 |  | 0.0787 |  |
| τ00 | 0.0420 | 0.2207 | | |
| N | 93 taxa | 2 sowing_id | | |
|  | 2 sowing_id |  | | |
| Observations | 434 | 434 | | |

| **13. Bayesian mixed-effects model for: RES(-)T1_S1_acc** | | | | |
| --- | --- | --- | --- | --- |
| *Predictors* | *Median* | *CI (95%)* | *Median* | *CI (95%)* |
| **Intercept** | **0.4235** | **0.2291 – 0.7446** | **0.3710** | **0.2409 – 0.4994** |
| Elevation | -0.0792 | -0.2351 – 0.0819 | -0.0854 | -0.2239 – 0.0523 |
| **Random effects** | |  |  |  |
| σ2 | 0.0028 |  |  |  |
| τ00 | 0.0135 |  |  |  |
| N | 62 taxa |  |  |  |
| Observations | 63 |  | 63 |  |

| **14. Bayesian mixed-effects model for: RES(-)T1_S2**  *Predictors Median CI (95%) Median* | | | | **_non-acc**  *CI (95%)* |
| --- | --- | --- | --- | --- |
| **Intercept** | **1.1050** | **0.9060 – 1.3166** | **1.0639** | **0.9863 – 1.1438** |
| Elevation | 0.0317 | -0.0543 – 0.1214 | 0.0072 | -0.0741 – 0.0907 |
| **Random effects** | |  |  |  |
| σ2 | 0.0022 |  |  |  |
| τ00 | 0.0031 |  |  |  |
| N | 87 taxa |  |  |  |
| Observations | 87 |  | 87 |  |

| **15. Bayesian mixed-effects model for: RES(-)T2_S1S2_acc**  *Predictors Median CI (95%) Median CI (95%)* | | | | | |
| --- | --- | --- | --- | --- | --- |
| **Intercept** | **-0.9520** | **-1.4950 – -0.4063** | **-0.9403** | **-1.5043 – -0.3974** |  |
| **Elevation** | 0.1230 | -0.0064 – 0.2488 | **0.1271** | **0.0183 – 0.2375** |  |
| **Random effects** | |  |  |  |  |
| σ2 | 0.0476 |  | 0.0451 |  |  |
| τ00 | 0.0173 |  | 0.0195 |  |  |
| N | 91 taxa |  | 2 sowing_id |  |  |
|  | 2 sowing_id |  |  |  |  |
| Observations | 146 |  | 146 |  |  |

| **16. Bayesian mixed-effects model for: RES(-)T2_non-acc** | | | | |
| --- | --- | --- | --- | --- |
| *Predictors* | *Median* | *CI (95%)* | *Median* | *CI (95%)* |
| **Intercept** | 0.0288 | -0.4267 – 0.3972 | **0.4109** | **0.2950 – 0.5227** |
| Elevation | 0.0433 | -0.0933 – 0.1789 | 0.0179 | -0.0934 – 0.1285 |
| **Random effects** | |  |  |  |
| σ2 | 0.0065 |  |  |  |
| τ00 | 0.0112 |  |  |  |
| N | 87 taxa |  |  |  |
| Observations | 87 |  | 87 |  |

| **17. Bayesian mixed-effects model for: RES(+)T1_S1_acc** | | | | |
| --- | --- | --- | --- | --- |
| *Predictors* | *Median* | *CI (95%)* | *Median* | *CI (95%)* |
| **Intercept** | **0.5506** | **0.1594 – 0.9724** | **0.3080** | **0.1530 – 0.4645** |
| Elevation | -0.0292 | -0.2017 – 0.1452 | 0.0327 | -0.1240 – 0.1884 |
| **Random effects** | |  |  |  |
| σ2 | 0.0140 |  |  |  |
| τ00 | 0.0029 |  |  |  |
| N | 46 taxa |  |  |  |
| Observations | 46 |  | 46 |  |

| **18. Bayesian mixed-effects model for: RES(+)T1_S2_non-acc** | | | | |
| --- | --- | --- | --- | --- |
| *Predictors* | *Median* | *CI (95%)* | *Median* | *CI (95%)* |
| **Intercept** | **0.7355** | **0.5232 – 0.9764** | **0.7053** | **0.6137 – 0.8008** |
| Elevation | 0.0436 | -0.0625 – 0.1525 | -0.0095 | -0.1065 – 0.0881 |
| **Random effects** | |  |  |  |
| σ2 | 0.0035 |  |  |  |
| τ00 | 0.0067 |  |  |  |
| N | 87 taxa |  |  |  |
| Observations | 87 |  | 87 |  |

| **19. Bayesian mixed-effects model for: RES(+)T2_S1S2**  *Predictors Median CI (95%) Median* | | | | **_acc**  *CI (95%)* |
| --- | --- | --- | --- | --- |
| **Intercept** | **-1.2419** | **-1.6665 – -0.6740** | **-1.2997** | **-1.6170 – -0.7160** |
| Elevation | 0.0259 | -0.1764 – 0.2121 | 0.0924 | -0.0575 – 0.2325 |
| **Random effects** | |  |  |  |
| σ2 | 0.0030 |  | -0.0019 |  |
| τ00 | 0.0202 |  | 0.0260 |  |
| N | 86 taxa |  | 2 sowing_id |  |
|  | 2 sowing_id |  |  |  |
| Observations | 132 |  | 132 |  |

| **20. Bayesian mixed-effects model for: RES(+)T2_S2_non-acc** | | | | |
| --- | --- | --- | --- | --- |
| *Predictors* | *Median* | *CI (95%)* | *Median* | *CI (95%)* |
| **Intercept** | **-1.7528** | **-2.1135 – -1.3187** | **-2.0883** | **-2.2421 – -1.9197** |
| **Elevation** | **-0.1760** | **-0.3477 – -0.0061** | **-0.1899** | **-0.3311 – -0.0498** |
| **Random effects** | |  |  |  |
| σ2 | 0.0014 |  |  |  |
| τ00 | 0.0041 |  |  |  |
| N | 87 taxa |  |  |  |
| Observations | 87 |  | 87 |  |

| **21. Bayesian mixed-effects model for: TOL_IGR** | | | | |
| --- | --- | --- | --- | --- |
| *Predictors* | *Median* | *CI (95%)* | *Median* | *CI (95%)* |
| Intercept | 0.0119 | -0.3223 – 0.3207 | 0.0131 | -0.3311 – 0.3387 |
| Elevation | 0.0125 | -0.0219 – 0.0474 | 0.0115 | -0.0224 – 0.0446 |
| Treatment (Heat vs Frost) | 0.0024 | -0.0449 – 0.0498 | 0.0031 | -0.0439 – 0.0513 |
| Elevation x Heat treatment vs Frost | 0.0122 | -0.0343 – 0.0607 | 0.0131 | -0.0333 – 0.0624 |
| **Random effects** |  |  |  |  |
| σ2 | 0.0008 |  | -0.0000 |  |
| τ00 | 0.0223 |  | 0.0228 |  |
| N | 80 taxa |  | 1 sowing_id |  |
|  | 1 sowing_id | | |  |
| Observations | 150 |  | 150 |  |

| **22. Bayesian mixed-effects model for: TOL_MGR** | | | | |
| --- | --- | --- | --- | --- |
| *Predictors* | *Median* | *CI (95%)* | *Median* | *CI (95%)* |
| Intercept | 0.0157 | -0.3524 – 0.3723 | 0.1655 | -0.2196 – 0.4161 |
| Elevation | 0.0827 | -0.1707 – 0.3372 | 0.0358 | -0.1618 – 0.2359 |
| **Treatment (Heat vs Frost)** | **1.4953** | **1.2338 – 1.7401** | **1.3725** | **1.0969 – 1.6343** |
| **Elevation x Heat treatment vs Frost** | **0.3153** | **0.0721 – 0.5639** | **0.3057** | **0.0222 – 0.5661** |
| **Random effects** |  |  |  |  |
| σ2 | 0.5017 |  | 0.0089 |  |
| τ00 | 2.0030 |  | 2.4691 |  |
| N | 80 taxa |  | 2 sowing_id |  |
|  | 2 sowing_id | | |  |
| Observations | 300 |  | 300 |  |

| **23. Bayesian mixed-effects model for: TOL_XMID** | | | | |
| --- | --- | --- | --- | --- |
| *Predictors* | *Median* | *CI (95%)* | *Median* | *CI (95%)* |
| Intercept | -0.0044 | -0.1870 – 0.1980 | -0.0256 | -0.2053 – 0.1654 |
| Elevation | 0.0080 | -0.0230 – 0.0384 | 0.0036 | -0.0230 – 0.0295 |
| Treatment (Heat vs Frost) | -0.0175 | -0.0536 – 0.0186 | -0.0134 | -0.0508 – 0.0237 |
| **Elevation x Heat treatment vs Frost** | **-0.0475** | **-0.0819 – -0.0126** | **-0.0502** | **-0.0874 – -0.0135** |
| **Random effects** |  |  |  |  |
| σ2 | 0.0042 |  | 0.0001 |  |
| τ00 | 0.0254 |  | 0.0296 |  |
| N | 80 taxa |  | 2 sowing_id |  |
|  | 2 sowing_id |  |  |  |
| Observations | 300 |  | 300 |  |

| **24. Bayesian mixed-effects model for: TOL_ASYM** | | | | |
| --- | --- | --- | --- | --- |
| *Predictors* | *Median* | *CI (95%)* | *Median* | *CI (95%)* |
| Intercept | -0.0289 | -0.5527 – 0.5166 | -0.0351 | -0.4709 – 0.3984 |
| Elevation | 0.0134 | -0.1312 – 0.1627 | -0.0601 | -0.1776 – 0.0580 |
| **Treatment (Heat vs Frost)** | **0.4121** | **0.2602 – 0.5589** | **0.4036** | **0.2326 – 0.5726** |
| **Elevation x Heat treatment vs Frost** | **-0.3033** | **-0.4485 – -0.1560** | **-0.3018** | **-0.4668 – -0.1374** |
| **Random effects** |  |  |  |  |
| σ2 | 0.2109 |  | 0.0034 |  |
| τ00 | 0.5894 |  | 0.7897 |  |
| N | 80 taxa |  | 2 sowing_id |  |
|  | 2 sowing_id |  |  |  |
| Observations | 300 |  | 300 |  |

**Table S2.** Comparison between mixed-effects models with *vs* without the consideration of the phylogenetic relationship among species (i.e., phylogeny as random effect). Comparison was performed via the expected log pointwise predictive density (ELPD) using leave-one-out cross validation (LOO). Indicated in bold is when one model was preferred over the other (range given by ELPD difference ± 2 standard error [SE] not overlapping with 0). Negative values of ELPD indicate that the model including the phylogeny performed better, positive values that the model without the phylogeny performed better. In red are the traits for which there was a significant effect of elevation or its interaction with treatment (under considering the phylogeny or not). Models were run on XMID and TOL_XMID.

Trait Trait ID ELPD_diff ± SE

| **Seed size** | | **SSIZ** | | | **-** | **81.1** | | | **±** | **13.1** | |
| --- | --- | --- | --- | --- | --- | --- | --- | --- | --- | --- | --- |
| Time to germination | | TGER | | | - | 6 | | | ± | 3.4 | |
| Initial growth rate | | IGR | | | + | 0.6 | | | ± | 0.8 | |
| Maximal growth rate | | MGR | | | + | 0.5 | | | ± | 0.9 | |
| **Time to fastest growth** | | **XMID** | | | **-** | **40** | | | **±** | **9.2** | |
| **Asymptotic size** | | **ASYM** | | | **-** | **279.3** | | | **±** | **22.1** | |
| **Number of leaves S2** | | **NLEA** | | | **-** | **239.6** | | | **±** | **24.4** | |
| **Leaf area** | | **LA** | | | **-** | **266.1** | | | **±** | **27.2** | |
| **Specific leaf area** | | **SLA** | | | **-** | **66.8** | | | **±** | **17.5** | |
| **Leaf dry matter content** | | **LDMC** | | | **-** | **98** | | | **±** | **14.1** | |
| **Leaf thickness S2** | | **LTh** | | | **-** | **21.5** | | | **±** | **6.8** | |
| **Leaf dissection index** | | **LDI** | | | **-** | **305.6** | | | **±** | **21.5** | |
| Frost resistance | |  | | |  |  | | |  |  | |
| *...acclimated (1h at -6 °C)* _S1_ | | RES(-)T1 | | | + | 0.5 | | | ± | 1.1 | |
| *...non-acclimated (1h at -5 °C)* S2 | | RES(-)T1 | | | - | 8 | | | ± | 4.8 | |
| *...acclimated (1h at -11 °C)* | | RES(-)T2 | | | - | 0.4 | | | ± | 2.2 | |
| *...non-acclimated (1h at -10 °C)* _S2_ | | RES(-)T2 | | | - | 1.8 | | | ± | 3.3 | |
| Heat resistance |  | |  |  | | |  |  | | |  |
| ***...acclimated (1h at +47 °C)* _S1_** | | **RES(+)T1** | | | **-** | **20.7** | | | **±** | **4.7** | |
| *...non-acclimated (1h at +45 °C)* S2 | | RES(+)T1 | | | - | 6.3 | | | ± | 3.2 | |
| *...acclimated (1h at +51 °C)* | | RES(+)T2 | | | + | 6.6 | | | ± | 3.3 | |
| *non-acclimated (1h at +50 °C)* S2 | | RES(+)T2 | | | - | 7.5 | | | ± | 4.1 | |
| Tolerance IGR | | TOL_IGR | | | + | 0.9 | | | ± | 0.8 | |
| **Tolerance MGR** | | **TOL_MGR** | | | **-** | **31.3** | | | **±** | **7.6** | |
| Tolerance XMID | | TOL_XMID | | | - | 7.2 | | | ± | 4.5 | |
| **Tolerance ASYM** | | **TOL_ASYM** | | | **-** | **27.9** | | | **±** | **8.8** | |

**
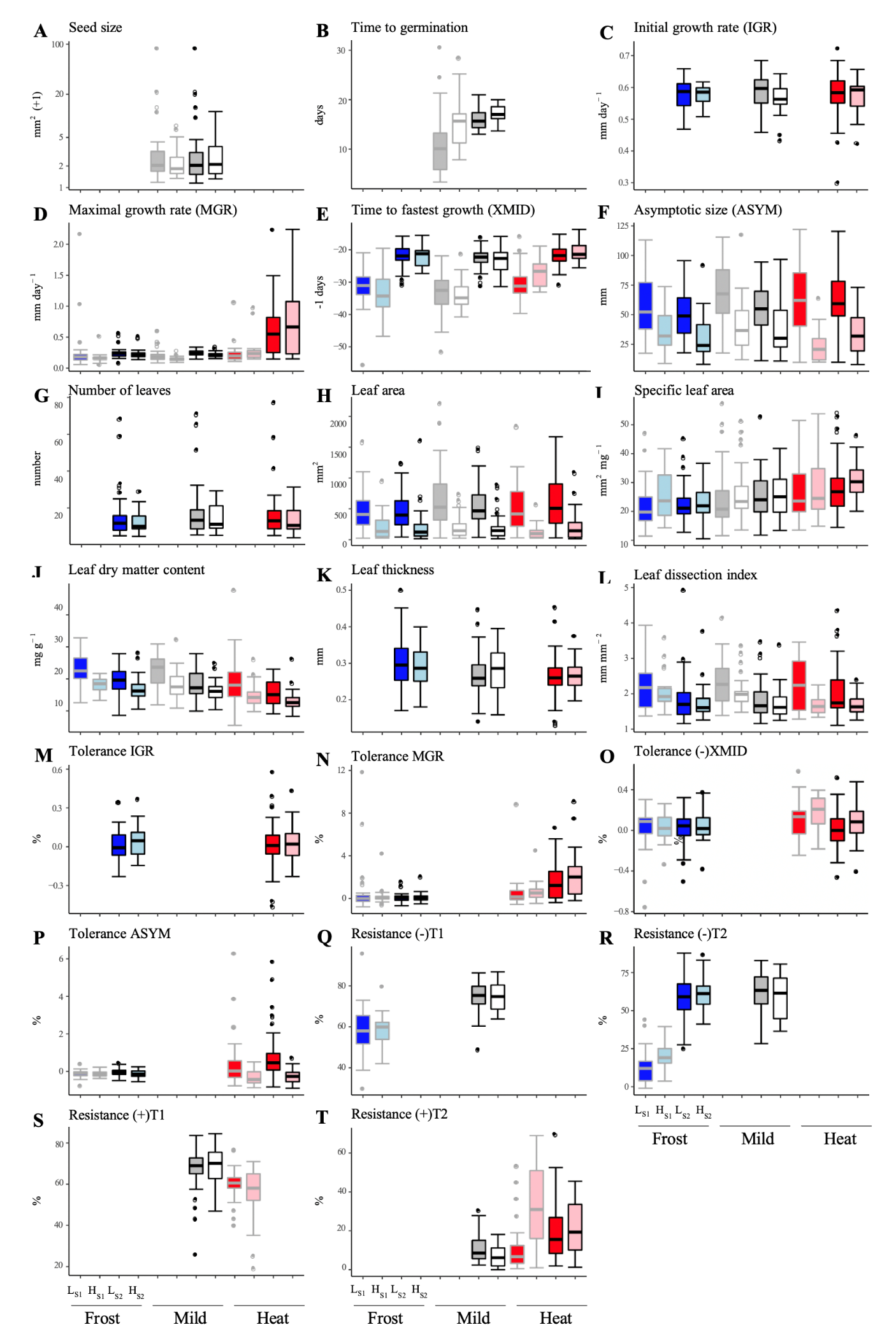
**

**Figure S2.** Boxplots showing the distribution of species-mean trait values for low- (L) *vs* high-elevation species (H) in the three growth treatments (regular frost, mild, regular heat). Colors inside boxes represent the treatments (blue for frost, greyscale for mild, and red for heat), while the color intensity represents median elevation of species occurrence (darker colors for low elevation and lighter colors for high elevation). Colors of the frames of boxes, whiskers and outliers represents the two rounds of sowing (grey S1, black S2). Boxplots depict medians, the lower and upper 25^th^ and 75^th^ percentiles, the most extreme data points < 1.5 * IQR away from hinges, and values beyond those ranges.
