# Supplementary material for: Trait divergence and trade‐offs among Brassicaceae species differing in elevational distribution
Source: Evolution. 2022 Jul 20;76(9):1986–2003. doi: 10.1111/evo.14554 (PMC9545065; doi:10.1111/evo.14554)
Supplement: Supplementary file 5 — SI5 – Multivariate analysis, and Pearson's correlations (with Table S4 and Figs. S6 to S10) [file EVO-76-1986-s003.docx]

Supporting information SI5 – Multivariate analysis, and Pearson’s correlations

**Table S4.** Correlations between all possible pairs of traits expressed in the three growth treatments (based on means between S1 and S2). Pearson’s correlation coefficients and the associated p-values were estimated with 'rcorr' {Hmisc}. C : Control/mild; F : Frost; H : Heat. Significance is indicated: * p < 0.05.

| **Trait_1** | **Trait_2** | **corr.coef** | **p-value** |  |
| --- | --- | --- | --- | --- |
| ASYM.C | LA.C | 0.810 | 0.000 | * |
| ASYM.C | LDI.C | 0.630 | 0.000 | * |
| ASYM.C | LDMC.C | 0.490 | 0.000 | * |
| ASYM.C | MGR.C | 0.210 | 0.080 |  |
| ASYM.C | SLA.C | -0.320 | 0.007 | * |
| ASYM.C | SSIZ.C | 0.130 | 0.299 |  |
| ASYM.C | TGER.C | -0.290 | 0.017 | * |
| ASYM.C | (-)XMID.C | -0.040 | 0.743 |  |
| ASYM.C | ASYM.F | 0.900 | 0.000 | * |
| ASYM.C | LA.F | 0.770 | 0.000 | * |
| ASYM.C | LDI.F | 0.600 | 0.000 | * |
| ASYM.C | LDMC.F | 0.430 | 0.000 | * |
| ASYM.C | MGR.F | 0.060 | 0.640 |  |
| ASYM.C | RES(-)T2.F | -0.260 | 0.031 | * |
| ASYM.C | SLA.F | -0.290 | 0.017 | * |
| ASYM.C | TOL_ASYM.F | -0.080 | 0.507 |  |
| ASYM.C | TOL_MGR.F | -0.130 | 0.274 |  |
| ASYM.C | TOL_(-)XMID.F | -0.010 | 0.929 |  |
| ASYM.C | (-)XMID.F | -0.030 | 0.814 |  |
| ASYM.C | ASYM.H | 0.880 | 0.000 | * |
| ASYM.C | LA.H | 0.780 | 0.000 | * |
| ASYM.C | LDI.H | 0.670 | 0.000 | * |
| ASYM.C | LDMC.H | 0.440 | 0.000 | * |
| ASYM.C | MGR.H | -0.050 | 0.659 |  |
| ASYM.C | RES(+)T2.H | -0.220 | 0.076 |  |
| ASYM.C | SLA.H | -0.390 | 0.001 | * |
| ASYM.C | TOL_ASYM.H | 0.570 | 0.000 | * |
| ASYM.C | TOL_MGR.H | 0.120 | 0.318 |  |
| ASYM.C | TOL_(-)XMID.H | -0.280 | 0.021 | * |
| ASYM.C | (-)XMID.H | -0.320 | 0.007 | * |
| ASYM.C | IGR.C | 0.260 | 0.033 | * |
| ASYM.C | LTH.C | 0.120 | 0.324 |  |
| ASYM.C | NLEA.C | -0.320 | 0.007 | * |
| ASYM.C | RES(-)T1.C | 0.050 | 0.697 |  |
| ASYM.C | RES(-)T2.C | 0.270 | 0.022 | * |
| ASYM.C | RES(+)T1.C | 0.200 | 0.104 |  |
| ASYM.C | RES(+)T2.C | 0.020 | 0.887 |  |
| ASYM.C | IGR.F | 0.030 | 0.801 |  |
| ASYM.C | LTH.F | -0.110 | 0.371 |  |
| ASYM.C | NLEA.F | -0.360 | 0.003 | * |
| ASYM.C | TOL_IGR.F | -0.200 | 0.100 |  |
| ASYM.C | IGR.H | -0.010 | 0.933 |  |
| ASYM.C | LTH.H | 0.040 | 0.720 |  |

| **Trait_1** | **Trait_2** | **corr.coef** | **p-value** |  |
| --- | --- | --- | --- | --- |
| ASYM.C | NLEA.H | -0.290 | 0.015 | * |
| ASYM.C | TOL_IGR.H | 0.050 | 0.694 |  |
| ASYM.C | LA.C | 0.810 | 0.000 | * |
| LA.C | LDI.C | 0.500 | 0.000 | * |
| LA.C | LDMC.C | 0.500 | 0.000 | * |
| LA.C | MGR.C | 0.200 | 0.102 |  |
| LA.C | SLA.C | -0.400 | 0.001 | * |
| LA.C | SSIZ.C | 0.180 | 0.138 |  |
| LA.C | TGER.C | -0.180 | 0.141 |  |
| LA.C | (-)XMID.C | 0.100 | 0.429 |  |
| ASYM.F | LA.C | 0.840 | 0.000 | * |
| LA.C | LA.F | 0.930 | 0.000 | * |
| LA.C | LDI.F | 0.480 | 0.000 | * |
| LA.C | LDMC.F | 0.420 | 0.000 | * |
| LA.C | MGR.F | 0.050 | 0.674 |  |
| LA.C | RES(-)T2.F | -0.300 | 0.012 | * |
| LA.C | SLA.F | -0.420 | 0.000 | * |
| LA.C | TOL_ASYM.F | 0.070 | 0.575 |  |
| LA.C | TOL_MGR.F | -0.070 | 0.544 |  |
| LA.C | TOL_(-)XMID.F | -0.180 | 0.137 |  |
| LA.C | (-)XMID.F | -0.070 | 0.593 |  |
| ASYM.H | LA.C | 0.870 | 0.000 | * |
| LA.C | LA.H | 0.930 | 0.000 | * |
| LA.C | LDI.H | 0.610 | 0.000 | * |
| LA.C | LDMC.H | 0.480 | 0.000 | * |
| LA.C | MGR.H | -0.070 | 0.557 |  |
| LA.C | RES(+)T2.H | -0.190 | 0.126 |  |
| LA.C | SLA.H | -0.520 | 0.000 | * |
| LA.C | TOL_ASYM.H | 0.560 | 0.000 | * |
| LA.C | TOL_MGR.H | 0.070 | 0.546 |  |
| LA.C | TOL_(-)XMID.H | -0.360 | 0.003 | * |
| LA.C | (-)XMID.H | -0.360 | 0.003 | * |
| IGR.C | LA.C | 0.250 | 0.041 | * |
| LA.C | LTH.C | 0.180 | 0.141 |  |
| LA.C | NLEA.C | -0.450 | 0.000 | * |
| LA.C | RES(-)T1.C | 0.180 | 0.146 |  |
| LA.C | RES(-)T2.C | 0.160 | 0.195 |  |
| LA.C | RES(+)T1.C | 0.210 | 0.091 |  |
| LA.C | RES(+)T2.C | 0.070 | 0.593 |  |
| IGR.F | LA.C | 0.060 | 0.604 |  |
| LA.C | LTH.F | -0.030 | 0.782 |  |
| LA.C | NLEA.F | -0.430 | 0.000 | * |
| LA.C | TOL_IGR.F | -0.200 | 0.099 |  |

| **Trait_1** | **Trait_2** | **corr.coef** | **p-value** |  |
| --- | --- | --- | --- | --- |
| IGR.H | LA.C | 0.040 | 0.721 |  |
| LA.C | LTH.H | 0.120 | 0.312 |  |
| LA.C | NLEA.H | -0.420 | 0.000 | * |
| LA.C | TOL_IGR.H | 0.080 | 0.536 |  |
| ASYM.C | LDI.C | 0.630 | 0.000 | * |
| LA.C | LDI.C | 0.500 | 0.000 | * |
| LDI.C | LDMC.C | 0.300 | 0.013 | * |
| LDI.C | MGR.C | 0.110 | 0.349 |  |
| LDI.C | SLA.C | 0.020 | 0.894 |  |
| LDI.C | SSIZ.C | -0.150 | 0.205 |  |
| LDI.C | TGER.C | -0.120 | 0.336 |  |
| LDI.C | (-)XMID.C | -0.030 | 0.837 |  |
| ASYM.F | LDI.C | 0.660 | 0.000 | * |
| LA.F | LDI.C | 0.410 | 0.000 | * |
| LDI.C | LDI.F | 0.950 | 0.000 | * |
| LDI.C | LDMC.F | 0.320 | 0.008 | * |
| LDI.C | MGR.F | -0.020 | 0.882 |  |
| LDI.C | RES(-)T2.F | -0.060 | 0.633 |  |
| LDI.C | SLA.F | 0.060 | 0.624 |  |
| LDI.C | TOL_ASYM.F | 0.020 | 0.855 |  |
| LDI.C | TOL_MGR.F | -0.080 | 0.527 |  |
| LDI.C | TOL_(-)XMID.F | 0.020 | 0.843 |  |
| LDI.C | (-)XMID.F | -0.060 | 0.648 |  |
| ASYM.H | LDI.C | 0.650 | 0.000 | * |
| LA.H | LDI.C | 0.440 | 0.000 | * |
| LDI.C | LDI.H | 0.920 | 0.000 | * |
| LDI.C | LDMC.H | 0.340 | 0.004 | * |
| LDI.C | MGR.H | -0.020 | 0.839 |  |
| LDI.C | RES(+)T2.H | -0.100 | 0.424 |  |
| LDI.C | SLA.H | -0.110 | 0.352 |  |
| LDI.C | TOL_ASYM.H | 0.430 | 0.000 | * |
| LDI.C | TOL_MGR.H | 0.170 | 0.155 |  |
| LDI.C | TOL_(-)XMID.H | -0.330 | 0.006 | * |
| LDI.C | (-)XMID.H | -0.230 | 0.058 |  |
| IGR.C | LDI.C | 0.200 | 0.108 |  |
| LDI.C | LTH.C | -0.260 | 0.034 | * |
| LDI.C | NLEA.C | -0.020 | 0.845 |  |
| LDI.C | RES(-)T1.C | -0.190 | 0.109 |  |
| LDI.C | RES(-)T2.C | 0.140 | 0.266 |  |
| LDI.C | RES(+)T1.C | -0.110 | 0.353 |  |
| LDI.C | RES(+)T2.C | 0.030 | 0.780 |  |
| IGR.F | LDI.C | 0.040 | 0.723 |  |
| LDI.C | LTH.F | -0.390 | 0.001 | * |
| LDI.C | NLEA.F | 0.000 | 0.975 |  |
| LDI.C | TOL_IGR.F | -0.210 | 0.086 |  |
| IGR.H | LDI.C | -0.140 | 0.242 |  |
| LDI.C | LTH.H | 0.040 | 0.728 |  |
| LDI.C | NLEA.H | 0.020 | 0.898 |  |
| LDI.C | TOL_IGR.H | 0.030 | 0.803 |  |

| **Trait_1** | **Trait_2** | **corr.coef** | **p-value** |  |
| --- | --- | --- | --- | --- |
| ASYM.C | LDMC.C | 0.490 | 0.000 | * |
| LA.C | LDMC.C | 0.500 | 0.000 | * |
| LDI.C | LDMC.C | 0.300 | 0.013 | * |
| LDMC.C | MGR.C | 0.200 | 0.097 |  |
| LDMC.C | SLA.C | -0.490 | 0.000 | * |
| LDMC.C | SSIZ.C | 0.190 | 0.124 |  |
| LDMC.C | TGER.C | -0.140 | 0.240 |  |
| LDMC.C | (-)XMID.C | 0.140 | 0.235 |  |
| ASYM.F | LDMC.C | 0.560 | 0.000 | * |
| LA.F | LDMC.C | 0.530 | 0.000 | * |
| LDI.F | LDMC.C | 0.310 | 0.009 | * |
| LDMC.C | LDMC.F | 0.790 | 0.000 | * |
| LDMC.C | MGR.F | -0.020 | 0.862 |  |
| LDMC.C | RES(-)T2.F | -0.320 | 0.008 | * |
| LDMC.C | SLA.F | -0.460 | 0.000 | * |
| LDMC.C | TOL_ASYM.F | 0.090 | 0.468 |  |
| LDMC.C | TOL_MGR.F | -0.030 | 0.827 |  |
| LDMC.C | TOL_(-)XMID.F | 0.030 | 0.805 |  |
| LDMC.C | (-)XMID.F | 0.080 | 0.515 |  |
| ASYM.H | LDMC.C | 0.530 | 0.000 | * |
| LA.H | LDMC.C | 0.540 | 0.000 | * |
| LDI.H | LDMC.C | 0.390 | 0.001 | * |
| LDMC.C | LDMC.H | 0.790 | 0.000 | * |
| LDMC.C | MGR.H | -0.200 | 0.095 |  |
| LDMC.C | RES(+)T2.H | 0.150 | 0.221 |  |
| LDMC.C | SLA.H | -0.510 | 0.000 | * |
| LDMC.C | TOL_ASYM.H | 0.350 | 0.003 | * |
| LDMC.C | TOL_MGR.H | -0.130 | 0.304 |  |
| LDMC.C | TOL_(-)XMID.H | -0.140 | 0.258 |  |
| LDMC.C | (-)XMID.H | -0.220 | 0.064 |  |
| IGR.C | LDMC.C | 0.130 | 0.292 |  |
| LDMC.C | LTH.C | 0.050 | 0.706 |  |
| LDMC.C | NLEA.C | -0.180 | 0.147 |  |
| LDMC.C | RES(-)T1.C | -0.090 | 0.454 |  |
| LDMC.C | RES(-)T2.C | 0.200 | 0.095 |  |
| LDMC.C | RES(+)T1.C | 0.190 | 0.128 |  |
| LDMC.C | RES(+)T2.C | 0.230 | 0.059 |  |
| IGR.F | LDMC.C | -0.150 | 0.223 |  |
| LDMC.C | LTH.F | -0.250 | 0.035 | * |
| LDMC.C | NLEA.F | -0.110 | 0.375 |  |
| LDMC.C | TOL_IGR.F | -0.160 | 0.186 |  |
| IGR.H | LDMC.C | 0.130 | 0.296 |  |
| LDMC.C | LTH.H | 0.020 | 0.889 |  |
| LDMC.C | NLEA.H | -0.060 | 0.602 |  |
| LDMC.C | TOL_IGR.H | 0.110 | 0.369 |  |
| ASYM.C | MGR.C | 0.210 | 0.080 |  |
| LA.C | MGR.C | 0.200 | 0.102 |  |
| LDI.C | MGR.C | 0.110 | 0.349 |  |
| LDMC.C | MGR.C | 0.200 | 0.097 |  |

| **Trait_1** | **Trait_2** | **corr.coef** | **p-value** |  |
| --- | --- | --- | --- | --- |
| MGR.C | SLA.C | -0.230 | 0.062 |  |
| MGR.C | SSIZ.C | 0.030 | 0.794 |  |
| MGR.C | TGER.C | -0.030 | 0.830 |  |
| MGR.C | (-)XMID.C | 0.170 | 0.153 |  |
| ASYM.F | MGR.C | 0.270 | 0.025 | * |
| LA.F | MGR.C | 0.220 | 0.064 |  |
| LDI.F | MGR.C | 0.150 | 0.220 |  |
| LDMC.F | MGR.C | 0.100 | 0.415 |  |
| MGR.C | MGR.F | 0.140 | 0.238 |  |
| MGR.C | RES(-)T2.F | -0.100 | 0.403 |  |
| MGR.C | SLA.F | -0.150 | 0.211 |  |
| MGR.C | TOL_ASYM.F | 0.130 | 0.271 |  |
| MGR.C | TOL_MGR.F | -0.350 | 0.003 | * |
| MGR.C | TOL_(-)XMID.F | -0.100 | 0.423 |  |
| MGR.C | (-)XMID.F | -0.080 | 0.531 |  |
| ASYM.H | MGR.C | 0.260 | 0.030 | * |
| LA.H | MGR.C | 0.150 | 0.230 |  |
| LDI.H | MGR.C | 0.060 | 0.626 |  |
| LDMC.H | MGR.C | 0.090 | 0.452 |  |
| MGR.C | MGR.H | -0.070 | 0.576 |  |
| MGR.C | RES(+)T2.H | -0.130 | 0.284 |  |
| MGR.C | SLA.H | -0.270 | 0.027 | * |
| MGR.C | TOL_ASYM.H | 0.150 | 0.209 |  |
| MGR.C | TOL_MGR.H | -0.190 | 0.128 |  |
| MGR.C | TOL_(-)XMID.H | 0.030 | 0.836 |  |
| MGR.C | (-)XMID.H | -0.150 | 0.226 |  |
| IGR.C | MGR.C | 0.030 | 0.827 |  |
| LTH.C | MGR.C | 0.060 | 0.649 |  |
| MGR.C | NLEA.C | 0.040 | 0.739 |  |
| MGR.C | RES(-)T1.C | 0.080 | 0.498 |  |
| MGR.C | RES(-)T2.C | 0.020 | 0.890 |  |
| MGR.C | RES(+)T1.C | 0.180 | 0.142 |  |
| MGR.C | RES(+)T2.C | 0.120 | 0.320 |  |
| IGR.F | MGR.C | 0.130 | 0.298 |  |
| LTH.F | MGR.C | 0.020 | 0.896 |  |
| MGR.C | NLEA.F | 0.050 | 0.656 |  |
| MGR.C | TOL_IGR.F | -0.040 | 0.726 |  |
| IGR.H | MGR.C | 0.110 | 0.376 |  |
| LTH.H | MGR.C | 0.050 | 0.667 |  |
| MGR.C | NLEA.H | 0.080 | 0.521 |  |
| MGR.C | TOL_IGR.H | 0.180 | 0.149 |  |
| ASYM.C | SLA.C | -0.320 | 0.007 | * |
| LA.C | SLA.C | -0.400 | 0.001 | * |
| LDI.C | SLA.C | 0.020 | 0.894 |  |
| LDMC.C | SLA.C | -0.490 | 0.000 | * |
| MGR.C | SLA.C | -0.230 | 0.062 |  |
| SLA.C | SSIZ.C | -0.470 | 0.000 | * |
| SLA.C | TGER.C | 0.090 | 0.447 |  |
| (-)XMID.C | SLA.C | -0.010 | 0.941 |  |

| **Trait_1** | **Trait_2** | **corr.coef** | **p-value** |  |
| --- | --- | --- | --- | --- |
| ASYM.F | SLA.C | -0.310 | 0.009 | * |
| LA.F | SLA.C | -0.360 | 0.002 | * |
| LDI.F | SLA.C | -0.010 | 0.933 |  |
| LDMC.F | SLA.C | -0.320 | 0.007 | * |
| MGR.F | SLA.C | 0.100 | 0.392 |  |
| RES(-)T2.F | SLA.C | 0.260 | 0.030 | * |
| SLA.C | SLA.F | 0.800 | 0.000 | * |
| SLA.C | TOL_ASYM.F | 0.080 | 0.528 |  |
| SLA.C | TOL_MGR.F | 0.150 | 0.219 |  |
| SLA.C | TOL_(-)XMID.F | 0.190 | 0.110 |  |
| (-)XMID.F | SLA.C | 0.140 | 0.258 |  |
| ASYM.H | SLA.C | -0.290 | 0.015 | * |
| LA.H | SLA.C | -0.350 | 0.003 | * |
| LDI.H | SLA.C | -0.080 | 0.512 |  |
| LDMC.H | SLA.C | -0.300 | 0.012 | * |
| MGR.H | SLA.C | 0.040 | 0.752 |  |
| RES(+)T2.H | SLA.C | 0.030 | 0.820 |  |
| SLA.C | SLA.H | 0.680 | 0.000 | * |
| SLA.C | TOL_ASYM.H | -0.060 | 0.639 |  |
| SLA.C | TOL_MGR.H | 0.020 | 0.886 |  |
| SLA.C | TOL_(-)XMID.H | -0.100 | 0.404 |  |
| (-)XMID.H | SLA.C | 0.220 | 0.064 |  |
| IGR.C | SLA.C | -0.080 | 0.506 |  |
| LTH.C | SLA.C | -0.560 | 0.000 | * |
| NLEA.C | SLA.C | 0.410 | 0.000 | * |
| RES(-)T1.C | SLA.C | -0.370 | 0.002 | * |
| RES(-)T2.C | SLA.C | -0.100 | 0.410 |  |
| RES(+)T1.C | SLA.C | -0.460 | 0.000 | * |
| RES(+)T2.C | SLA.C | 0.200 | 0.107 |  |
| IGR.F | SLA.C | -0.010 | 0.924 |  |
| LTH.F | SLA.C | -0.330 | 0.005 | * |
| NLEA.F | SLA.C | 0.340 | 0.004 | * |
| SLA.C | TOL_IGR.F | 0.110 | 0.385 |  |
| IGR.H | SLA.C | -0.260 | 0.032 | * |
| LTH.H | SLA.C | -0.180 | 0.133 |  |
| NLEA.H | SLA.C | 0.400 | 0.001 | * |
| SLA.C | TOL_IGR.H | -0.060 | 0.633 |  |
| ASYM.C | SSIZ.C | 0.130 | 0.299 |  |
| LA.C | SSIZ.C | 0.180 | 0.138 |  |
| LDI.C | SSIZ.C | -0.150 | 0.205 |  |
| LDMC.C | SSIZ.C | 0.190 | 0.124 |  |
| MGR.C | SSIZ.C | 0.030 | 0.794 |  |
| SLA.C | SSIZ.C | -0.470 | 0.000 | * |
| SSIZ.C | TGER.C | 0.210 | 0.081 |  |
| (-)XMID.C | SSIZ.C | 0.340 | 0.004 | * |
| ASYM.F | SSIZ.C | 0.150 | 0.211 |  |
| LA.F | SSIZ.C | 0.210 | 0.083 |  |
| LDI.F | SSIZ.C | -0.230 | 0.061 |  |
| LDMC.F | SSIZ.C | 0.170 | 0.169 |  |

| **Trait_1** | **Trait_2** | **corr.coef** | **p-value** |  |
| --- | --- | --- | --- | --- |
| MGR.F | SSIZ.C | 0.010 | 0.940 |  |
| RES(-)T2.F | SSIZ.C | -0.170 | 0.150 |  |
| SLA.F | SSIZ.C | -0.610 | 0.000 | * |
| SSIZ.C | TOL_ASYM.F | 0.000 | 0.995 |  |
| SSIZ.C | TOL_MGR.F | -0.110 | 0.358 |  |
| SSIZ.C | TOL_(-)XMID.F | -0.220 | 0.070 |  |
| (-)XMID.F | SSIZ.C | 0.150 | 0.220 |  |
| ASYM.H | SSIZ.C | 0.120 | 0.338 |  |
| LA.H | SSIZ.C | 0.210 | 0.086 |  |
| LDI.H | SSIZ.C | -0.150 | 0.228 |  |
| LDMC.H | SSIZ.C | 0.170 | 0.153 |  |
| MGR.H | SSIZ.C | -0.070 | 0.552 |  |
| RES(+)T2.H | SSIZ.C | 0.040 | 0.726 |  |
| SLA.H | SSIZ.C | -0.470 | 0.000 | * |
| SSIZ.C | TOL_ASYM.H | 0.050 | 0.686 |  |
| SSIZ.C | TOL_MGR.H | 0.010 | 0.954 |  |
| SSIZ.C | TOL_(-)XMID.H | 0.080 | 0.506 |  |
| (-)XMID.H | SSIZ.C | 0.190 | 0.109 |  |
| IGR.C | SSIZ.C | 0.150 | 0.232 |  |
| LTH.C | SSIZ.C | 0.400 | 0.001 | * |
| NLEA.C | SSIZ.C | -0.430 | 0.000 | * |
| RES(-)T1.C | SSIZ.C | 0.270 | 0.026 | * |
| RES(-)T2.C | SSIZ.C | -0.030 | 0.806 |  |
| RES(+)T1.C | SSIZ.C | 0.370 | 0.002 | * |
| RES(+)T2.C | SSIZ.C | -0.140 | 0.237 |  |
| IGR.F | SSIZ.C | -0.170 | 0.159 |  |
| LTH.F | SSIZ.C | 0.290 | 0.015 | * |
| NLEA.F | SSIZ.C | -0.430 | 0.000 | * |
| SSIZ.C | TOL_IGR.F | -0.260 | 0.031 | * |
| IGR.H | SSIZ.C | 0.110 | 0.348 |  |
| LTH.H | SSIZ.C | 0.120 | 0.336 |  |
| NLEA.H | SSIZ.C | -0.430 | 0.000 | * |
| SSIZ.C | TOL_IGR.H | 0.020 | 0.856 |  |
| ASYM.C | TGER.C | -0.290 | 0.017 | * |
| LA.C | TGER.C | -0.180 | 0.141 |  |
| LDI.C | TGER.C | -0.120 | 0.336 |  |
| LDMC.C | TGER.C | -0.140 | 0.240 |  |
| MGR.C | TGER.C | -0.030 | 0.830 |  |
| SLA.C | TGER.C | 0.090 | 0.447 |  |
| SSIZ.C | TGER.C | 0.210 | 0.081 |  |
| (-)XMID.C | TGER.C | 0.390 | 0.001 | * |
| ASYM.F | TGER.C | -0.300 | 0.011 | * |
| LA.F | TGER.C | -0.220 | 0.076 |  |
| LDI.F | TGER.C | -0.140 | 0.264 |  |
| LDMC.F | TGER.C | -0.240 | 0.050 |  |
| MGR.F | TGER.C | 0.030 | 0.776 |  |
| RES(-)T2.F | TGER.C | 0.300 | 0.013 | * |
| SLA.F | TGER.C | 0.070 | 0.587 |  |
| TGER.C | TOL_ASYM.F | 0.030 | 0.836 |  |

| **Trait_1** | **Trait_2** | **corr.coef** | **p-value** |  |
| --- | --- | --- | --- | --- |
| TGER.C | TOL_MGR.F | 0.000 | 0.970 |  |
| TGER.C | TOL_(-)XMID.F | -0.100 | 0.431 |  |
| (-)XMID.F | TGER.C | 0.370 | 0.002 | * |
| ASYM.H | TGER.C | -0.260 | 0.033 | * |
| LA.H | TGER.C | -0.190 | 0.111 |  |
| LDI.H | TGER.C | -0.170 | 0.151 |  |
| LDMC.H | TGER.C | -0.140 | 0.251 |  |
| MGR.H | TGER.C | 0.150 | 0.233 |  |
| RES(+)T2.H | TGER.C | 0.180 | 0.132 |  |
| SLA.H | TGER.C | 0.040 | 0.766 |  |
| TGER.C | TOL_ASYM.H | -0.310 | 0.009 | * |
| TGER.C | TOL_MGR.H | -0.010 | 0.959 |  |
| TGER.C | TOL_(-)XMID.H | 0.050 | 0.710 |  |
| (-)XMID.H | TGER.C | 0.430 | 0.000 | * |
| IGR.C | TGER.C | -0.170 | 0.175 |  |
| LTH.C | TGER.C | -0.150 | 0.222 |  |
| NLEA.C | TGER.C | -0.310 | 0.009 | * |
| RES(-)T1.C | TGER.C | 0.000 | 0.990 |  |
| RES(-)T2.C | TGER.C | -0.320 | 0.007 | * |
| RES(+)T1.C | TGER.C | -0.110 | 0.362 |  |
| RES(+)T2.C | TGER.C | -0.100 | 0.392 |  |
| IGR.F | TGER.C | 0.060 | 0.650 |  |
| LTH.F | TGER.C | 0.000 | 0.969 |  |
| NLEA.F | TGER.C | -0.290 | 0.017 | * |
| TGER.C | TOL_IGR.F | 0.170 | 0.168 |  |
| IGR.H | TGER.C | 0.030 | 0.827 |  |
| LTH.H | TGER.C | 0.060 | 0.642 |  |
| NLEA.H | TGER.C | -0.250 | 0.042 | * |
| TGER.C | TOL_IGR.H | 0.020 | 0.871 |  |
| ASYM.C | (-)XMID.C | -0.040 | 0.743 |  |
| LA.C | (-)XMID.C | 0.100 | 0.429 |  |
| LDI.C | (-)XMID.C | -0.030 | 0.837 |  |
| LDMC.C | (-)XMID.C | 0.140 | 0.235 |  |
| MGR.C | (-)XMID.C | 0.170 | 0.153 |  |
| (-)XMID.C | SLA.C | -0.010 | 0.941 |  |
| (-)XMID.C | SSIZ.C | 0.340 | 0.004 | * |
| (-)XMID.C | TGER.C | 0.390 | 0.001 | * |
| ASYM.F | (-)XMID.C | 0.050 | 0.688 |  |
| LA.F | (-)XMID.C | 0.180 | 0.142 |  |
| LDI.F | (-)XMID.C | -0.030 | 0.804 |  |
| LDMC.F | (-)XMID.C | 0.040 | 0.756 |  |
| MGR.F | (-)XMID.C | 0.170 | 0.171 |  |
| (-)XMID.C | RES(-)T2.F | 0.150 | 0.205 |  |
| (-)XMID.C | SLA.F | -0.100 | 0.405 |  |
| (-)XMID.C | TOL_ASYM.F | 0.290 | 0.016 | * |
| (-)XMID.C | TOL_MGR.F | 0.030 | 0.837 |  |
| (-)XMID.C | TOL_(-)XMID.F | -0.260 | 0.029 | * |
| (-)XMID.C | (-)XMID.F | 0.440 | 0.000 | * |
| ASYM.H | (-)XMID.C | 0.110 | 0.377 |  |

| **Trait_1** | **Trait_2** | **corr.coef** | **p-value** |  |
| --- | --- | --- | --- | --- |
| LA.H | (-)XMID.C | 0.170 | 0.153 |  |
| LDI.H | (-)XMID.C | -0.010 | 0.934 |  |
| LDMC.H | (-)XMID.C | 0.080 | 0.509 |  |
| MGR.H | (-)XMID.C | -0.190 | 0.125 |  |
| (-)XMID.C | RES(+)T2.H | -0.080 | 0.534 |  |
| (-)XMID.C | SLA.H | -0.050 | 0.689 |  |
| (-)XMID.C | TOL_ASYM.H | -0.020 | 0.886 |  |
| (-)XMID.C | TOL_MGR.H | -0.180 | 0.128 |  |
| (-)XMID.C | TOL_(-)XMID.H | 0.170 | 0.162 |  |
| (-)XMID.C | (-)XMID.H | 0.430 | 0.000 | * |
| IGR.C | (-)XMID.C | 0.030 | 0.787 |  |
| LTH.C | (-)XMID.C | 0.060 | 0.644 |  |
| (-)XMID.C | NLEA.C | -0.150 | 0.204 |  |
| (-)XMID.C | RES(-)T1.C | 0.080 | 0.504 |  |
| (-)XMID.C | RES(-)T2.C | 0.040 | 0.735 |  |
| (-)XMID.C | RES(+)T1.C | 0.000 | 0.998 |  |
| (-)XMID.C | RES(+)T2.C | -0.030 | 0.806 |  |
| IGR.F | (-)XMID.C | -0.140 | 0.256 |  |
| LTH.F | (-)XMID.C | 0.010 | 0.926 |  |
| (-)XMID.C | NLEA.F | -0.090 | 0.487 |  |
| (-)XMID.C | TOL_IGR.F | -0.120 | 0.337 |  |
| IGR.H | (-)XMID.C | -0.010 | 0.910 |  |
| LTH.H | (-)XMID.C | 0.090 | 0.464 |  |
| (-)XMID.C | NLEA.H | -0.100 | 0.414 |  |
| (-)XMID.C | TOL_IGR.H | 0.060 | 0.636 |  |
| ASYM.C | ASYM.F | 0.900 | 0.000 | * |
| ASYM.F | LA.C | 0.840 | 0.000 | * |
| ASYM.F | LDI.C | 0.660 | 0.000 | * |
| ASYM.F | LDMC.C | 0.560 | 0.000 | * |
| ASYM.F | MGR.C | 0.270 | 0.025 | * |
| ASYM.F | SLA.C | -0.310 | 0.009 | * |
| ASYM.F | SSIZ.C | 0.150 | 0.211 |  |
| ASYM.F | TGER.C | -0.300 | 0.011 | * |
| ASYM.F | (-)XMID.C | 0.050 | 0.688 |  |
| ASYM.F | LA.F | 0.860 | 0.000 | * |
| ASYM.F | LDI.F | 0.650 | 0.000 | * |
| ASYM.F | LDMC.F | 0.510 | 0.000 | * |
| ASYM.F | MGR.F | -0.010 | 0.951 |  |
| ASYM.F | RES(-)T2.F | -0.270 | 0.026 | * |
| ASYM.F | SLA.F | -0.350 | 0.003 | * |
| ASYM.F | TOL_ASYM.F | 0.210 | 0.081 |  |
| ASYM.F | TOL_MGR.F | -0.100 | 0.410 |  |
| ASYM.F | TOL_(-)XMID.F | -0.130 | 0.274 |  |
| ASYM.F | (-)XMID.F | -0.130 | 0.294 |  |
| ASYM.F | ASYM.H | 0.900 | 0.000 | * |
| ASYM.F | LA.H | 0.820 | 0.000 | * |
| ASYM.F | LDI.H | 0.710 | 0.000 | * |
| ASYM.F | LDMC.H | 0.510 | 0.000 | * |
| ASYM.F | MGR.H | -0.050 | 0.702 |  |

| **Trait_1** | **Trait_2** | **corr.coef** | **p-value** |  |
| --- | --- | --- | --- | --- |
| ASYM.F | RES(+)T2.H | -0.210 | 0.083 |  |
| ASYM.F | SLA.H | -0.460 | 0.000 | * |
| ASYM.F | TOL_ASYM.H | 0.610 | 0.000 | * |
| ASYM.F | TOL_MGR.H | 0.100 | 0.402 |  |
| ASYM.F | TOL_(-)XMID.H | -0.320 | 0.007 | * |
| ASYM.F | (-)XMID.H | -0.350 | 0.003 | * |
| ASYM.F | IGR.C | 0.300 | 0.012 | * |
| ASYM.F | LTH.C | 0.120 | 0.332 |  |
| ASYM.F | NLEA.C | -0.320 | 0.008 | * |
| ASYM.F | RES(-)T1.C | 0.050 | 0.669 |  |
| ASYM.F | RES(-)T2.C | 0.260 | 0.033 | * |
| ASYM.F | RES(+)T1.C | 0.190 | 0.115 |  |
| ASYM.F | RES(+)T2.C | 0.120 | 0.338 |  |
| ASYM.F | IGR.F | 0.020 | 0.886 |  |
| ASYM.F | LTH.F | -0.180 | 0.129 |  |
| ASYM.F | NLEA.F | -0.300 | 0.013 | * |
| ASYM.F | TOL_IGR.F | -0.280 | 0.020 | * |
| ASYM.F | IGR.H | -0.040 | 0.725 |  |
| ASYM.F | LTH.H | 0.070 | 0.591 |  |
| ASYM.F | NLEA.H | -0.260 | 0.033 | * |
| ASYM.F | TOL_IGR.H | 0.080 | 0.534 |  |
| ASYM.C | LA.F | 0.770 | 0.000 | * |
| LA.C | LA.F | 0.930 | 0.000 | * |
| LA.F | LDI.C | 0.410 | 0.000 | * |
| LA.F | LDMC.C | 0.530 | 0.000 | * |
| LA.F | MGR.C | 0.220 | 0.064 |  |
| LA.F | SLA.C | -0.360 | 0.002 | * |
| LA.F | SSIZ.C | 0.210 | 0.083 |  |
| LA.F | TGER.C | -0.220 | 0.076 |  |
| LA.F | (-)XMID.C | 0.180 | 0.142 |  |
| ASYM.F | LA.F | 0.860 | 0.000 | * |
| LA.F | LDI.F | 0.430 | 0.000 | * |
| LA.F | LDMC.F | 0.450 | 0.000 | * |
| LA.F | MGR.F | 0.040 | 0.723 |  |
| LA.F | RES(-)T2.F | -0.230 | 0.061 |  |
| LA.F | SLA.F | -0.440 | 0.000 | * |
| LA.F | TOL_ASYM.F | 0.160 | 0.194 |  |
| LA.F | TOL_MGR.F | -0.040 | 0.738 |  |
| LA.F | TOL_(-)XMID.F | -0.110 | 0.348 |  |
| LA.F | (-)XMID.F | -0.010 | 0.903 |  |
| ASYM.H | LA.F | 0.850 | 0.000 | * |
| LA.F | LA.H | 0.940 | 0.000 | * |
| LA.F | LDI.H | 0.540 | 0.000 | * |
| LA.F | LDMC.H | 0.520 | 0.000 | * |
| LA.F | MGR.H | -0.070 | 0.586 |  |
| LA.F | RES(+)T2.H | -0.210 | 0.089 |  |
| LA.F | SLA.H | -0.560 | 0.000 | * |
| LA.F | TOL_ASYM.H | 0.540 | 0.000 | * |
| LA.F | TOL_MGR.H | 0.050 | 0.663 |  |

| **Trait_1** | **Trait_2** | **corr.coef** | **p-value** |  |
| --- | --- | --- | --- | --- |
| LA.F | TOL_(-)XMID.H | -0.310 | 0.009 | * |
| LA.F | (-)XMID.H | -0.290 | 0.017 | * |
| IGR.C | LA.F | 0.230 | 0.060 |  |
| LA.F | LTH.C | 0.190 | 0.110 |  |
| LA.F | NLEA.C | -0.490 | 0.000 | * |
| LA.F | RES(-)T1.C | 0.170 | 0.153 |  |
| LA.F | RES(-)T2.C | 0.160 | 0.190 |  |
| LA.F | RES(+)T1.C | 0.220 | 0.066 |  |
| LA.F | RES(+)T2.C | 0.120 | 0.319 |  |
| IGR.F | LA.F | 0.060 | 0.649 |  |
| LA.F | LTH.F | -0.060 | 0.626 |  |
| LA.F | NLEA.F | -0.470 | 0.000 | * |
| LA.F | TOL_IGR.F | -0.190 | 0.109 |  |
| IGR.H | LA.F | 0.040 | 0.754 |  |
| LA.F | LTH.H | 0.140 | 0.260 |  |
| LA.F | NLEA.H | -0.440 | 0.000 | * |
| LA.F | TOL_IGR.H | 0.080 | 0.538 |  |
| ASYM.C | LDI.F | 0.600 | 0.000 | * |
| LA.C | LDI.F | 0.480 | 0.000 | * |
| LDI.C | LDI.F | 0.950 | 0.000 | * |
| LDI.F | LDMC.C | 0.310 | 0.009 | * |
| LDI.F | MGR.C | 0.150 | 0.220 |  |
| LDI.F | SLA.C | -0.010 | 0.933 |  |
| LDI.F | SSIZ.C | -0.230 | 0.061 |  |
| LDI.F | TGER.C | -0.140 | 0.264 |  |
| LDI.F | (-)XMID.C | -0.030 | 0.804 |  |
| ASYM.F | LDI.F | 0.650 | 0.000 | * |
| LA.F | LDI.F | 0.430 | 0.000 | * |
| LDI.F | LDMC.F | 0.300 | 0.013 | * |
| LDI.F | MGR.F | 0.000 | 0.976 |  |
| LDI.F | RES(-)T2.F | -0.010 | 0.966 |  |
| LDI.F | SLA.F | 0.070 | 0.572 |  |
| LDI.F | TOL_ASYM.F | 0.090 | 0.442 |  |
| LDI.F | TOL_MGR.F | -0.030 | 0.793 |  |
| LDI.F | TOL_(-)XMID.F | 0.010 | 0.960 |  |
| LDI.F | (-)XMID.F | -0.100 | 0.428 |  |
| ASYM.H | LDI.F | 0.610 | 0.000 | * |
| LA.H | LDI.F | 0.430 | 0.000 | * |
| LDI.F | LDI.H | 0.890 | 0.000 | * |
| LDI.F | LDMC.H | 0.320 | 0.007 | * |
| LDI.F | MGR.H | -0.020 | 0.850 |  |
| LDI.F | RES(+)T2.H | -0.070 | 0.575 |  |
| LDI.F | SLA.H | -0.110 | 0.349 |  |
| LDI.F | TOL_ASYM.H | 0.390 | 0.001 | * |
| LDI.F | TOL_MGR.H | 0.180 | 0.150 |  |
| LDI.F | TOL_(-)XMID.H | -0.260 | 0.032 | * |
| LDI.F | (-)XMID.H | -0.210 | 0.080 |  |
| IGR.C | LDI.F | 0.240 | 0.046 | * |
| LDI.F | LTH.C | -0.210 | 0.081 |  |

| **Trait_1** | **Trait_2** | **corr.coef** | **p-value** |  |
| --- | --- | --- | --- | --- |
| LDI.F | NLEA.C | -0.030 | 0.835 |  |
| LDI.F | RES(-)T1.C | -0.210 | 0.084 |  |
| LDI.F | RES(-)T2.C | 0.200 | 0.104 |  |
| LDI.F | RES(+)T1.C | -0.150 | 0.214 |  |
| LDI.F | RES(+)T2.C | 0.030 | 0.831 |  |
| IGR.F | LDI.F | 0.040 | 0.741 |  |
| LDI.F | LTH.F | -0.320 | 0.008 | * |
| LDI.F | NLEA.F | 0.000 | 0.998 |  |
| LDI.F | TOL_IGR.F | -0.250 | 0.035 | * |
| IGR.H | LDI.F | -0.120 | 0.319 |  |
| LDI.F | LTH.H | 0.020 | 0.889 |  |
| LDI.F | NLEA.H | 0.010 | 0.953 |  |
| LDI.F | TOL_IGR.H | 0.040 | 0.763 |  |
| ASYM.C | LDMC.F | 0.430 | 0.000 | * |
| LA.C | LDMC.F | 0.420 | 0.000 | * |
| LDI.C | LDMC.F | 0.320 | 0.008 | * |
| LDMC.C | LDMC.F | 0.790 | 0.000 | * |
| LDMC.F | MGR.C | 0.100 | 0.415 |  |
| LDMC.F | SLA.C | -0.320 | 0.007 | * |
| LDMC.F | SSIZ.C | 0.170 | 0.169 |  |
| LDMC.F | TGER.C | -0.240 | 0.050 |  |
| LDMC.F | (-)XMID.C | 0.040 | 0.756 |  |
| ASYM.F | LDMC.F | 0.510 | 0.000 | * |
| LA.F | LDMC.F | 0.450 | 0.000 | * |
| LDI.F | LDMC.F | 0.300 | 0.013 | * |
| LDMC.F | MGR.F | -0.110 | 0.368 |  |
| LDMC.F | RES(-)T2.F | -0.440 | 0.000 | * |
| LDMC.F | SLA.F | -0.500 | 0.000 | * |
| LDMC.F | TOL_ASYM.F | 0.090 | 0.444 |  |
| LDMC.F | TOL_MGR.F | 0.060 | 0.604 |  |
| LDMC.F | TOL_(-)XMID.F | 0.030 | 0.828 |  |
| LDMC.F | (-)XMID.F | -0.040 | 0.716 |  |
| ASYM.H | LDMC.F | 0.500 | 0.000 | * |
| LA.H | LDMC.F | 0.460 | 0.000 | * |
| LDI.H | LDMC.F | 0.360 | 0.002 | * |
| LDMC.F | LDMC.H | 0.730 | 0.000 | * |
| LDMC.F | MGR.H | -0.210 | 0.076 |  |
| LDMC.F | RES(+)T2.H | 0.030 | 0.788 |  |
| LDMC.F | SLA.H | -0.430 | 0.000 | * |
| LDMC.F | TOL_ASYM.H | 0.380 | 0.001 | * |
| LDMC.F | TOL_MGR.H | -0.060 | 0.600 |  |
| LDMC.F | TOL_(-)XMID.H | -0.160 | 0.181 |  |
| LDMC.F | (-)XMID.H | -0.240 | 0.044 | * |
| IGR.C | LDMC.F | 0.030 | 0.831 |  |
| LDMC.F | LTH.C | -0.080 | 0.502 |  |
| LDMC.F | NLEA.C | -0.030 | 0.791 |  |
| LDMC.F | RES(-)T1.C | -0.210 | 0.089 |  |
| LDMC.F | RES(-)T2.C | 0.110 | 0.368 |  |
| LDMC.F | RES(+)T1.C | 0.100 | 0.415 |  |

| **Trait_1** | **Trait_2** | **corr.coef** | **p-value** |  |
| --- | --- | --- | --- | --- |
| LDMC.F | RES(+)T2.C | 0.300 | 0.012 | * |
| IGR.F | LDMC.F | -0.110 | 0.348 |  |
| LDMC.F | LTH.F | -0.340 | 0.005 | * |
| LDMC.F | NLEA.F | 0.000 | 0.978 |  |
| LDMC.F | TOL_IGR.F | -0.090 | 0.484 |  |
| IGR.H | LDMC.F | 0.050 | 0.669 |  |
| LDMC.F | LTH.H | -0.090 | 0.457 |  |
| LDMC.F | NLEA.H | 0.070 | 0.595 |  |
| LDMC.F | TOL_IGR.H | 0.120 | 0.321 |  |
| ASYM.C | MGR.F | 0.060 | 0.640 |  |
| LA.C | MGR.F | 0.050 | 0.674 |  |
| LDI.C | MGR.F | -0.020 | 0.882 |  |
| LDMC.C | MGR.F | -0.020 | 0.862 |  |
| MGR.C | MGR.F | 0.140 | 0.238 |  |
| MGR.F | SLA.C | 0.100 | 0.392 |  |
| MGR.F | SSIZ.C | 0.010 | 0.940 |  |
| MGR.F | TGER.C | 0.030 | 0.776 |  |
| MGR.F | (-)XMID.C | 0.170 | 0.171 |  |
| ASYM.F | MGR.F | -0.010 | 0.951 |  |
| LA.F | MGR.F | 0.040 | 0.723 |  |
| LDI.F | MGR.F | 0.000 | 0.976 |  |
| LDMC.F | MGR.F | -0.110 | 0.368 |  |
| MGR.F | RES(-)T2.F | -0.030 | 0.806 |  |
| MGR.F | SLA.F | 0.190 | 0.117 |  |
| MGR.F | TOL_ASYM.F | 0.010 | 0.960 |  |
| MGR.F | TOL_MGR.F | 0.350 | 0.003 | * |
| MGR.F | TOL_(-)XMID.F | -0.010 | 0.940 |  |
| MGR.F | (-)XMID.F | 0.210 | 0.089 |  |
| ASYM.H | MGR.F | -0.010 | 0.927 |  |
| LA.H | MGR.F | 0.060 | 0.648 |  |
| LDI.H | MGR.F | -0.050 | 0.710 |  |
| LDMC.H | MGR.F | 0.040 | 0.770 |  |
| MGR.F | MGR.H | 0.090 | 0.462 |  |
| MGR.F | RES(+)T2.H | 0.000 | 0.981 |  |
| MGR.F | SLA.H | 0.120 | 0.320 |  |
| MGR.F | TOL_ASYM.H | 0.000 | 0.992 |  |
| MGR.F | TOL_MGR.H | 0.100 | 0.434 |  |
| MGR.F | TOL_(-)XMID.H | 0.030 | 0.789 |  |
| MGR.F | (-)XMID.H | 0.160 | 0.199 |  |
| IGR.C | MGR.F | 0.130 | 0.296 |  |
| LTH.C | MGR.F | -0.010 | 0.947 |  |
| MGR.F | NLEA.C | 0.110 | 0.382 |  |
| MGR.F | RES(-)T1.C | -0.120 | 0.327 |  |
| MGR.F | RES(-)T2.C | 0.070 | 0.573 |  |
| MGR.F | RES(+)T1.C | -0.070 | 0.587 |  |
| MGR.F | RES(+)T2.C | -0.130 | 0.304 |  |
| IGR.F | MGR.F | 0.120 | 0.324 |  |
| LTH.F | MGR.F | 0.000 | 0.989 |  |
| MGR.F | NLEA.F | 0.020 | 0.851 |  |

| **Trait_1** | **Trait_2** | **corr.coef** | **p-value** |  |
| --- | --- | --- | --- | --- |
| MGR.F | TOL_IGR.F | 0.000 | 0.999 |  |
| IGR.H | MGR.F | -0.040 | 0.766 |  |
| LTH.H | MGR.F | -0.050 | 0.665 |  |
| MGR.F | NLEA.H | 0.050 | 0.708 |  |
| MGR.F | TOL_IGR.H | 0.060 | 0.609 |  |
| ASYM.C | RES(-)T2.F | -0.260 | 0.031 | * |
| LA.C | RES(-)T2.F | -0.300 | 0.012 | * |
| LDI.C | RES(-)T2.F | -0.060 | 0.633 |  |
| LDMC.C | RES(-)T2.F | -0.320 | 0.008 | * |
| MGR.C | RES(-)T2.F | -0.100 | 0.403 |  |
| RES(-)T2.F | SLA.C | 0.260 | 0.030 | * |
| RES(-)T2.F | SSIZ.C | -0.170 | 0.150 |  |
| RES(-)T2.F | TGER.C | 0.300 | 0.013 | * |
| (-)XMID.C | RES(-)T2.F | 0.150 | 0.205 |  |
| ASYM.F | RES(-)T2.F | -0.270 | 0.026 | * |
| LA.F | RES(-)T2.F | -0.230 | 0.061 |  |
| LDI.F | RES(-)T2.F | -0.010 | 0.966 |  |
| LDMC.F | RES(-)T2.F | -0.440 | 0.000 | * |
| MGR.F | RES(-)T2.F | -0.030 | 0.806 |  |
| RES(-)T2.F | SLA.F | 0.260 | 0.032 | * |
| RES(-)T2.F | TOL_ASYM.F | 0.070 | 0.595 |  |
| RES(-)T2.F | TOL_MGR.F | 0.080 | 0.490 |  |
| RES(-)T2.F | TOL_(-)XMID.F | -0.030 | 0.811 |  |
| (-)XMID.F | RES(-)T2.F | 0.310 | 0.010 | * |
| ASYM.H | RES(-)T2.F | -0.190 | 0.123 |  |
| LA.H | RES(-)T2.F | -0.230 | 0.059 |  |
| LDI.H | RES(-)T2.F | -0.070 | 0.569 |  |
| LDMC.H | RES(-)T2.F | -0.280 | 0.020 | * |
| MGR.H | RES(-)T2.F | 0.260 | 0.034 | * |
| RES(-)T2.F | RES(+)T2.H | 0.070 | 0.592 |  |
| RES(-)T2.F | SLA.H | 0.230 | 0.062 |  |
| RES(-)T2.F | TOL_ASYM.H | -0.280 | 0.021 | * |
| RES(-)T2.F | TOL_MGR.H | 0.050 | 0.664 |  |
| RES(-)T2.F | TOL_(-)XMID.H | -0.010 | 0.955 |  |
| (-)XMID.H | RES(-)T2.F | 0.340 | 0.004 | * |
| IGR.C | RES(-)T2.F | -0.100 | 0.409 |  |
| LTH.C | RES(-)T2.F | -0.160 | 0.181 |  |
| NLEA.C | RES(-)T2.F | 0.000 | 0.983 |  |
| RES(-)T1.C | RES(-)T2.F | 0.220 | 0.066 |  |
| RES(-)T2.C | RES(-)T2.F | 0.110 | 0.377 |  |
| RES(-)T2.F | RES(+)T1.C | -0.010 | 0.925 |  |
| RES(-)T2.F | RES(+)T2.C | 0.080 | 0.502 |  |
| IGR.F | RES(-)T2.F | 0.050 | 0.662 |  |
| LTH.F | RES(-)T2.F | 0.050 | 0.654 |  |
| NLEA.F | RES(-)T2.F | 0.040 | 0.724 |  |
| RES(-)T2.F | TOL_IGR.F | 0.130 | 0.288 |  |
| IGR.H | RES(-)T2.F | 0.130 | 0.286 |  |
| LTH.H | RES(-)T2.F | 0.150 | 0.234 |  |
| NLEA.H | RES(-)T2.F | -0.030 | 0.786 |  |

| **Trait_1** | **Trait_2** | **corr.coef** | **p-value** |  |
| --- | --- | --- | --- | --- |
| RES(-)T2.F | TOL_IGR.H | 0.090 | 0.452 |  |
| ASYM.C | SLA.F | -0.290 | 0.017 | * |
| LA.C | SLA.F | -0.420 | 0.000 | * |
| LDI.C | SLA.F | 0.060 | 0.624 |  |
| LDMC.C | SLA.F | -0.460 | 0.000 | * |
| MGR.C | SLA.F | -0.150 | 0.211 |  |
| SLA.C | SLA.F | 0.800 | 0.000 | * |
| SLA.F | SSIZ.C | -0.610 | 0.000 | * |
| SLA.F | TGER.C | 0.070 | 0.587 |  |
| (-)XMID.C | SLA.F | -0.100 | 0.405 |  |
| ASYM.F | SLA.F | -0.350 | 0.003 | * |
| LA.F | SLA.F | -0.440 | 0.000 | * |
| LDI.F | SLA.F | 0.070 | 0.572 |  |
| LDMC.F | SLA.F | -0.500 | 0.000 | * |
| MGR.F | SLA.F | 0.190 | 0.117 |  |
| RES(-)T2.F | SLA.F | 0.260 | 0.032 | * |
| SLA.F | TOL_ASYM.F | 0.050 | 0.692 |  |
| SLA.F | TOL_MGR.F | 0.150 | 0.226 |  |
| SLA.F | TOL_(-)XMID.F | 0.140 | 0.249 |  |
| (-)XMID.F | SLA.F | 0.090 | 0.481 |  |
| ASYM.H | SLA.F | -0.320 | 0.007 | * |
| LA.H | SLA.F | -0.390 | 0.001 | * |
| LDI.H | SLA.F | -0.020 | 0.851 |  |
| LDMC.H | SLA.F | -0.380 | 0.001 | * |
| MGR.H | SLA.F | 0.150 | 0.234 |  |
| RES(+)T2.H | SLA.F | 0.060 | 0.651 |  |
| SLA.F | SLA.H | 0.760 | 0.000 | * |
| SLA.F | TOL_ASYM.H | -0.110 | 0.389 |  |
| SLA.F | TOL_MGR.H | 0.070 | 0.563 |  |
| SLA.F | TOL_(-)XMID.H | 0.030 | 0.822 |  |
| (-)XMID.H | SLA.F | 0.190 | 0.123 |  |
| IGR.C | SLA.F | -0.030 | 0.838 |  |
| LTH.C | SLA.F | -0.470 | 0.000 | * |
| NLEA.C | SLA.F | 0.430 | 0.000 | * |
| RES(-)T1.C | SLA.F | -0.410 | 0.001 | * |
| RES(-)T2.C | SLA.F | -0.070 | 0.579 |  |
| RES(+)T1.C | SLA.F | -0.490 | 0.000 | * |
| RES(+)T2.C | SLA.F | 0.070 | 0.559 |  |
| IGR.F | SLA.F | -0.020 | 0.858 |  |
| LTH.F | SLA.F | -0.310 | 0.009 | * |
| NLEA.F | SLA.F | 0.380 | 0.001 | * |
| SLA.F | TOL_IGR.F | 0.060 | 0.647 |  |
| IGR.H | SLA.F | -0.240 | 0.045 | * |
| LTH.H | SLA.F | -0.170 | 0.169 |  |
| NLEA.H | SLA.F | 0.440 | 0.000 | * |
| SLA.F | TOL_IGR.H | -0.060 | 0.649 |  |
| ASYM.C | TOL_ASYM.F | -0.080 | 0.507 |  |
| LA.C | TOL_ASYM.F | 0.070 | 0.575 |  |
| LDI.C | TOL_ASYM.F | 0.020 | 0.855 |  |

| **Trait_1** | **Trait_2** | **corr.coef** | **p-value** |  |
| --- | --- | --- | --- | --- |
| LDMC.C | TOL_ASYM.F | 0.090 | 0.468 |  |
| MGR.C | TOL_ASYM.F | 0.130 | 0.271 |  |
| SLA.C | TOL_ASYM.F | 0.080 | 0.528 |  |
| SSIZ.C | TOL_ASYM.F | 0.000 | 0.995 |  |
| TGER.C | TOL_ASYM.F | 0.030 | 0.836 |  |
| (-)XMID.C | TOL_ASYM.F | 0.290 | 0.016 | * |
| ASYM.F | TOL_ASYM.F | 0.210 | 0.081 |  |
| LA.F | TOL_ASYM.F | 0.160 | 0.194 |  |
| LDI.F | TOL_ASYM.F | 0.090 | 0.442 |  |
| LDMC.F | TOL_ASYM.F | 0.090 | 0.444 |  |
| MGR.F | TOL_ASYM.F | 0.010 | 0.960 |  |
| RES(-)T2.F | TOL_ASYM.F | 0.070 | 0.595 |  |
| SLA.F | TOL_ASYM.F | 0.050 | 0.692 |  |
| TOL_ASYM.F | TOL_MGR.F | 0.090 | 0.444 |  |
| TOL_ASYM.F | TOL_(-)XMID.F | -0.520 | 0.000 | * |
| (-)XMID.F | TOL_ASYM.F | -0.120 | 0.333 |  |
| ASYM.H | TOL_ASYM.F | 0.030 | 0.789 |  |
| LA.H | TOL_ASYM.F | 0.080 | 0.527 |  |
| LDI.H | TOL_ASYM.F | 0.050 | 0.656 |  |
| LDMC.H | TOL_ASYM.F | 0.020 | 0.858 |  |
| MGR.H | TOL_ASYM.F | -0.050 | 0.697 |  |
| RES(+)T2.H | TOL_ASYM.F | 0.090 | 0.456 |  |
| SLA.H | TOL_ASYM.F | -0.010 | 0.963 |  |
| TOL_ASYM.F | TOL_ASYM.H | 0.060 | 0.600 |  |
| TOL_ASYM.F | TOL_MGR.H | -0.130 | 0.292 |  |
| TOL_ASYM.F | TOL_(-)XMID.H | -0.040 | 0.737 |  |
| (-)XMID.H | TOL_ASYM.F | 0.110 | 0.385 |  |
| IGR.C | TOL_ASYM.F | 0.170 | 0.161 |  |
| LTH.C | TOL_ASYM.F | 0.010 | 0.945 |  |
| NLEA.C | TOL_ASYM.F | 0.090 | 0.447 |  |
| RES(-)T1.C | TOL_ASYM.F | -0.100 | 0.424 |  |
| RES(-)T2.C | TOL_ASYM.F | -0.070 | 0.566 |  |
| RES(+)T1.C | TOL_ASYM.F | -0.110 | 0.364 |  |
| RES(+)T2.C | TOL_ASYM.F | 0.250 | 0.041 | * |
| IGR.F | TOL_ASYM.F | -0.110 | 0.378 |  |
| LTH.F | TOL_ASYM.F | -0.140 | 0.243 |  |
| NLEA.F | TOL_ASYM.F | 0.150 | 0.228 |  |
| TOL_ASYM.F | TOL_IGR.F | -0.200 | 0.097 |  |
| IGR.H | TOL_ASYM.F | -0.210 | 0.086 |  |
| LTH.H | TOL_ASYM.F | -0.070 | 0.553 |  |
| NLEA.H | TOL_ASYM.F | 0.130 | 0.288 |  |
| TOL_ASYM.F | TOL_IGR.H | -0.070 | 0.588 |  |
| ASYM.C | TOL_MGR.F | -0.130 | 0.274 |  |
| LA.C | TOL_MGR.F | -0.070 | 0.544 |  |
| LDI.C | TOL_MGR.F | -0.080 | 0.527 |  |
| LDMC.C | TOL_MGR.F | -0.030 | 0.827 |  |
| MGR.C | TOL_MGR.F | -0.350 | 0.003 | * |
| SLA.C | TOL_MGR.F | 0.150 | 0.219 |  |
| SSIZ.C | TOL_MGR.F | -0.110 | 0.358 |  |

| **Trait_1** | **Trait_2** | **corr.coef** | **p-value** |  |
| --- | --- | --- | --- | --- |
| TGER.C | TOL_MGR.F | 0.000 | 0.970 |  |
| (-)XMID.C | TOL_MGR.F | 0.030 | 0.837 |  |
| ASYM.F | TOL_MGR.F | -0.100 | 0.410 |  |
| LA.F | TOL_MGR.F | -0.040 | 0.738 |  |
| LDI.F | TOL_MGR.F | -0.030 | 0.793 |  |
| LDMC.F | TOL_MGR.F | 0.060 | 0.604 |  |
| MGR.F | TOL_MGR.F | 0.350 | 0.003 | * |
| RES(-)T2.F | TOL_MGR.F | 0.080 | 0.490 |  |
| SLA.F | TOL_MGR.F | 0.150 | 0.226 |  |
| TOL_ASYM.F | TOL_MGR.F | 0.090 | 0.444 |  |
| TOL_MGR.F | TOL_(-)XMID.F | 0.010 | 0.953 |  |
| (-)XMID.F | TOL_MGR.F | 0.000 | 0.989 |  |
| ASYM.H | TOL_MGR.F | -0.080 | 0.539 |  |
| LA.H | TOL_MGR.F | -0.010 | 0.911 |  |
| LDI.H | TOL_MGR.F | -0.040 | 0.739 |  |
| LDMC.H | TOL_MGR.F | 0.060 | 0.651 |  |
| MGR.H | TOL_MGR.F | 0.000 | 0.986 |  |
| RES(+)T2.H | TOL_MGR.F | -0.080 | 0.493 |  |
| SLA.H | TOL_MGR.F | 0.270 | 0.025 | * |
| TOL_ASYM.H | TOL_MGR.F | -0.090 | 0.468 |  |
| TOL_MGR.F | TOL_MGR.H | 0.100 | 0.432 |  |
| TOL_MGR.F | TOL_(-)XMID.H | 0.030 | 0.827 |  |
| (-)XMID.H | TOL_MGR.F | 0.010 | 0.917 |  |
| IGR.C | TOL_MGR.F | -0.070 | 0.554 |  |
| LTH.C | TOL_MGR.F | -0.020 | 0.881 |  |
| NLEA.C | TOL_MGR.F | 0.000 | 0.982 |  |
| RES(-)T1.C | TOL_MGR.F | -0.190 | 0.113 |  |
| RES(-)T2.C | TOL_MGR.F | 0.060 | 0.629 |  |
| RES(+)T1.C | TOL_MGR.F | -0.070 | 0.553 |  |
| RES(+)T2.C | TOL_MGR.F | -0.010 | 0.950 |  |
| IGR.F | TOL_MGR.F | 0.060 | 0.643 |  |
| LTH.F | TOL_MGR.F | -0.090 | 0.471 |  |
| NLEA.F | TOL_MGR.F | -0.010 | 0.913 |  |
| TOL_IGR.F | TOL_MGR.F | 0.060 | 0.619 |  |
| IGR.H | TOL_MGR.F | -0.120 | 0.329 |  |
| LTH.H | TOL_MGR.F | -0.030 | 0.796 |  |
| NLEA.H | TOL_MGR.F | -0.060 | 0.637 |  |
| TOL_IGR.H | TOL_MGR.F | -0.170 | 0.157 |  |
| ASYM.C | TOL_(-)XMID.F | -0.010 | 0.929 |  |
| LA.C | TOL_(-)XMID.F | -0.180 | 0.137 |  |
| LDI.C | TOL_(-)XMID.F | 0.020 | 0.843 |  |
| LDMC.C | TOL_(-)XMID.F | 0.030 | 0.805 |  |
| MGR.C | TOL_(-)XMID.F | -0.100 | 0.423 |  |
| SLA.C | TOL_(-)XMID.F | 0.190 | 0.110 |  |
| SSIZ.C | TOL_(-)XMID.F | -0.220 | 0.070 |  |
| TGER.C | TOL_(-)XMID.F | -0.100 | 0.431 |  |
| (-)XMID.C | TOL_(-)XMID.F | -0.260 | 0.029 | * |
| ASYM.F | TOL_(-)XMID.F | -0.130 | 0.274 |  |
| LA.F | TOL_(-)XMID.F | -0.110 | 0.348 |  |

| **Trait_1** | **Trait_2** | **corr.coef** | **p-value** |  |
| --- | --- | --- | --- | --- |
| LDI.F | TOL_(-)XMID.F | 0.010 | 0.960 |  |
| LDMC.F | TOL_(-)XMID.F | 0.030 | 0.828 |  |
| MGR.F | TOL_(-)XMID.F | -0.010 | 0.940 |  |
| RES(-)T2.F | TOL_(-)XMID.F | -0.030 | 0.811 |  |
| SLA.F | TOL_(-)XMID.F | 0.140 | 0.249 |  |
| TOL_ASYM.F | TOL_(-)XMID.F | -0.520 | 0.000 | * |
| TOL_MGR.F | TOL_(-)XMID.F | 0.010 | 0.953 |  |
| (-)XMID.F | TOL_(-)XMID.F | 0.290 | 0.016 | * |
| ASYM.H | TOL_(-)XMID.F | -0.080 | 0.522 |  |
| LA.H | TOL_(-)XMID.F | -0.130 | 0.277 |  |
| LDI.H | TOL_(-)XMID.F | 0.020 | 0.868 |  |
| LDMC.H | TOL_(-)XMID.F | 0.110 | 0.367 |  |
| MGR.H | TOL_(-)XMID.F | 0.030 | 0.826 |  |
| RES(+)T2.H | TOL_(-)XMID.F | -0.030 | 0.787 |  |
| SLA.H | TOL_(-)XMID.F | 0.170 | 0.155 |  |
| TOL_ASYM.H | TOL_(-)XMID.F | -0.090 | 0.480 |  |
| TOL_MGR.H | TOL_(-)XMID.F | 0.130 | 0.296 |  |
| TOL_(-)XMID.F | TOL_(-)XMID.H | -0.160 | 0.182 |  |
| (-)XMID.H | TOL_(-)XMID.F | -0.190 | 0.126 |  |
| IGR.C | TOL_(-)XMID.F | -0.030 | 0.835 |  |
| LTH.C | TOL_(-)XMID.F | -0.080 | 0.526 |  |
| NLEA.C | TOL_(-)XMID.F | 0.040 | 0.731 |  |
| RES(-)T1.C | TOL_(-)XMID.F | -0.120 | 0.317 |  |
| RES(-)T2.C | TOL_(-)XMID.F | -0.150 | 0.211 |  |
| RES(+)T1.C | TOL_(-)XMID.F | -0.160 | 0.180 |  |
| RES(+)T2.C | TOL_(-)XMID.F | 0.000 | 0.986 |  |
| IGR.F | TOL_(-)XMID.F | 0.050 | 0.704 |  |
| LTH.F | TOL_(-)XMID.F | -0.150 | 0.222 |  |
| NLEA.F | TOL_(-)XMID.F | 0.050 | 0.683 |  |
| TOL_IGR.F | TOL_(-)XMID.F | 0.110 | 0.381 |  |
| IGR.H | TOL_(-)XMID.F | -0.060 | 0.618 |  |
| LTH.H | TOL_(-)XMID.F | 0.070 | 0.551 |  |
| NLEA.H | TOL_(-)XMID.F | 0.020 | 0.854 |  |
| TOL_IGR.H | TOL_(-)XMID.F | -0.070 | 0.590 |  |
| ASYM.C | (-)XMID.F | -0.030 | 0.814 |  |
| LA.C | (-)XMID.F | -0.070 | 0.593 |  |
| LDI.C | (-)XMID.F | -0.060 | 0.648 |  |
| LDMC.C | (-)XMID.F | 0.080 | 0.515 |  |
| MGR.C | (-)XMID.F | -0.080 | 0.531 |  |
| (-)XMID.F | SLA.C | 0.140 | 0.258 |  |
| (-)XMID.F | SSIZ.C | 0.150 | 0.220 |  |
| (-)XMID.F | TGER.C | 0.370 | 0.002 | * |
| (-)XMID.C | (-)XMID.F | 0.440 | 0.000 | * |
| ASYM.F | (-)XMID.F | -0.130 | 0.294 |  |
| LA.F | (-)XMID.F | -0.010 | 0.903 |  |
| LDI.F | (-)XMID.F | -0.100 | 0.428 |  |
| LDMC.F | (-)XMID.F | -0.040 | 0.716 |  |
| MGR.F | (-)XMID.F | 0.210 | 0.089 |  |
| (-)XMID.F | RES(-)T2.F | 0.310 | 0.010 | * |

| **Trait_1** | **Trait_2** | **corr.coef** | **p-value** |  |
| --- | --- | --- | --- | --- |
| (-)XMID.F | SLA.F | 0.090 | 0.481 |  |
| (-)XMID.F | TOL_ASYM.F | -0.120 | 0.333 |  |
| (-)XMID.F | TOL_MGR.F | 0.000 | 0.989 |  |
| (-)XMID.F | TOL_(-)XMID.F | 0.290 | 0.016 | * |
| ASYM.H | (-)XMID.F | -0.020 | 0.889 |  |
| LA.H | (-)XMID.F | 0.010 | 0.928 |  |
| LDI.H | (-)XMID.F | -0.020 | 0.891 |  |
| LDMC.H | (-)XMID.F | 0.030 | 0.832 |  |
| MGR.H | (-)XMID.F | 0.030 | 0.816 |  |
| (-)XMID.F | RES(+)T2.H | 0.080 | 0.494 |  |
| (-)XMID.F | SLA.H | 0.170 | 0.161 |  |
| (-)XMID.F | TOL_ASYM.H | -0.150 | 0.230 |  |
| (-)XMID.F | TOL_MGR.H | -0.100 | 0.433 |  |
| (-)XMID.F | TOL_(-)XMID.H | 0.040 | 0.723 |  |
| (-)XMID.F | (-)XMID.H | 0.470 | 0.000 | * |
| IGR.C | (-)XMID.F | -0.130 | 0.276 |  |
| LTH.C | (-)XMID.F | -0.040 | 0.727 |  |
| (-)XMID.F | NLEA.C | -0.020 | 0.865 |  |
| (-)XMID.F | RES(-)T1.C | -0.010 | 0.910 |  |
| (-)XMID.F | RES(-)T2.C | -0.040 | 0.720 |  |
| (-)XMID.F | RES(+)T1.C | -0.030 | 0.805 |  |
| (-)XMID.F | RES(+)T2.C | -0.080 | 0.521 |  |
| IGR.F | (-)XMID.F | -0.180 | 0.137 |  |
| LTH.F | (-)XMID.F | -0.100 | 0.405 |  |
| (-)XMID.F | NLEA.F | 0.020 | 0.891 |  |
| (-)XMID.F | TOL_IGR.F | 0.090 | 0.463 |  |
| IGR.H | (-)XMID.F | 0.120 | 0.346 |  |
| LTH.H | (-)XMID.F | 0.020 | 0.900 |  |
| (-)XMID.F | NLEA.H | 0.040 | 0.759 |  |
| (-)XMID.F | TOL_IGR.H | 0.150 | 0.211 |  |
| ASYM.C | ASYM.H | 0.880 | 0.000 | * |
| ASYM.H | LA.C | 0.870 | 0.000 | * |
| ASYM.H | LDI.C | 0.650 | 0.000 | * |
| ASYM.H | LDMC.C | 0.530 | 0.000 | * |
| ASYM.H | MGR.C | 0.260 | 0.030 | * |
| ASYM.H | SLA.C | -0.290 | 0.015 | * |
| ASYM.H | SSIZ.C | 0.120 | 0.338 |  |
| ASYM.H | TGER.C | -0.260 | 0.033 | * |
| ASYM.H | (-)XMID.C | 0.110 | 0.377 |  |
| ASYM.F | ASYM.H | 0.900 | 0.000 | * |
| ASYM.H | LA.F | 0.850 | 0.000 | * |
| ASYM.H | LDI.F | 0.610 | 0.000 | * |
| ASYM.H | LDMC.F | 0.500 | 0.000 | * |
| ASYM.H | MGR.F | -0.010 | 0.927 |  |
| ASYM.H | RES(-)T2.F | -0.190 | 0.123 |  |
| ASYM.H | SLA.F | -0.320 | 0.007 | * |
| ASYM.H | TOL_ASYM.F | 0.030 | 0.789 |  |
| ASYM.H | TOL_MGR.F | -0.080 | 0.539 |  |
| ASYM.H | TOL_(-)XMID.F | -0.080 | 0.522 |  |

| **Trait_1** | **Trait_2** | **corr.coef** | **p-value** |  |
| --- | --- | --- | --- | --- |
| ASYM.H | (-)XMID.F | -0.020 | 0.889 |  |
| ASYM.H | LA.H | 0.890 | 0.000 | * |
| ASYM.H | LDI.H | 0.730 | 0.000 | * |
| ASYM.H | LDMC.H | 0.480 | 0.000 | * |
| ASYM.H | MGR.H | -0.090 | 0.449 |  |
| ASYM.H | RES(+)T2.H | -0.280 | 0.019 | * |
| ASYM.H | SLA.H | -0.430 | 0.000 | * |
| ASYM.H | TOL_ASYM.H | 0.700 | 0.000 | * |
| ASYM.H | TOL_MGR.H | 0.030 | 0.794 |  |
| ASYM.H | TOL_(-)XMID.H | -0.380 | 0.001 | * |
| ASYM.H | (-)XMID.H | -0.380 | 0.001 | * |
| ASYM.H | IGR.C | 0.230 | 0.058 |  |
| ASYM.H | LTH.C | 0.050 | 0.687 |  |
| ASYM.H | NLEA.C | -0.360 | 0.003 | * |
| ASYM.H | RES(-)T1.C | 0.080 | 0.494 |  |
| ASYM.H | RES(-)T2.C | 0.210 | 0.088 |  |
| ASYM.H | RES(+)T1.C | 0.210 | 0.079 |  |
| ASYM.H | RES(+)T2.C | 0.100 | 0.393 |  |
| ASYM.H | IGR.F | 0.100 | 0.426 |  |
| ASYM.H | LTH.F | -0.150 | 0.206 |  |
| ASYM.H | NLEA.F | -0.330 | 0.005 | * |
| ASYM.H | TOL_IGR.F | -0.210 | 0.079 |  |
| ASYM.H | IGR.H | 0.040 | 0.714 |  |
| ASYM.H | LTH.H | 0.110 | 0.371 |  |
| ASYM.H | NLEA.H | -0.300 | 0.013 | * |
| ASYM.H | TOL_IGR.H | 0.110 | 0.369 |  |
| ASYM.C | LA.H | 0.780 | 0.000 | * |
| LA.C | LA.H | 0.930 | 0.000 | * |
| LA.H | LDI.C | 0.440 | 0.000 | * |
| LA.H | LDMC.C | 0.540 | 0.000 | * |
| LA.H | MGR.C | 0.150 | 0.230 |  |
| LA.H | SLA.C | -0.350 | 0.003 | * |
| LA.H | SSIZ.C | 0.210 | 0.086 |  |
| LA.H | TGER.C | -0.190 | 0.111 |  |
| LA.H | (-)XMID.C | 0.170 | 0.153 |  |
| ASYM.F | LA.H | 0.820 | 0.000 | * |
| LA.F | LA.H | 0.940 | 0.000 | * |
| LA.H | LDI.F | 0.430 | 0.000 | * |
| LA.H | LDMC.F | 0.460 | 0.000 | * |
| LA.H | MGR.F | 0.060 | 0.648 |  |
| LA.H | RES(-)T2.F | -0.230 | 0.059 |  |
| LA.H | SLA.F | -0.390 | 0.001 | * |
| LA.H | TOL_ASYM.F | 0.080 | 0.527 |  |
| LA.H | TOL_MGR.F | -0.010 | 0.911 |  |
| LA.H | TOL_(-)XMID.F | -0.130 | 0.277 |  |
| LA.H | (-)XMID.F | 0.010 | 0.928 |  |
| ASYM.H | LA.H | 0.890 | 0.000 | * |
| LA.H | LDI.H | 0.590 | 0.000 | * |
| LA.H | LDMC.H | 0.500 | 0.000 | * |

| **Trait_1** | **Trait_2** | **corr.coef** | **p-value** |  |
| --- | --- | --- | --- | --- |
| LA.H | MGR.H | -0.080 | 0.502 |  |
| LA.H | RES(+)T2.H | -0.160 | 0.200 |  |
| LA.H | SLA.H | -0.480 | 0.000 | * |
| LA.H | TOL_ASYM.H | 0.600 | 0.000 | * |
| LA.H | TOL_MGR.H | 0.030 | 0.783 |  |
| LA.H | TOL_(-)XMID.H | -0.330 | 0.005 | * |
| LA.H | (-)XMID.H | -0.310 | 0.010 | * |
| IGR.C | LA.H | 0.290 | 0.014 | * |
| LA.H | LTH.C | 0.180 | 0.133 |  |
| LA.H | NLEA.C | -0.470 | 0.000 | * |
| LA.H | RES(-)T1.C | 0.140 | 0.251 |  |
| LA.H | RES(-)T2.C | 0.160 | 0.198 |  |
| LA.H | RES(+)T1.C | 0.290 | 0.016 | * |
| LA.H | RES(+)T2.C | 0.060 | 0.650 |  |
| IGR.F | LA.H | 0.050 | 0.680 |  |
| LA.H | LTH.F | -0.060 | 0.641 |  |
| LA.H | NLEA.F | -0.460 | 0.000 | * |
| LA.H | TOL_IGR.F | -0.260 | 0.033 | * |
| IGR.H | LA.H | 0.040 | 0.742 |  |
| LA.H | LTH.H | 0.100 | 0.411 |  |
| LA.H | NLEA.H | -0.430 | 0.000 | * |
| LA.H | TOL_IGR.H | 0.030 | 0.822 |  |
| ASYM.C | LDI.H | 0.670 | 0.000 | * |
| LA.C | LDI.H | 0.610 | 0.000 | * |
| LDI.C | LDI.H | 0.920 | 0.000 | * |
| LDI.H | LDMC.C | 0.390 | 0.001 | * |
| LDI.H | MGR.C | 0.060 | 0.626 |  |
| LDI.H | SLA.C | -0.080 | 0.512 |  |
| LDI.H | SSIZ.C | -0.150 | 0.228 |  |
| LDI.H | TGER.C | -0.170 | 0.151 |  |
| LDI.H | (-)XMID.C | -0.010 | 0.934 |  |
| ASYM.F | LDI.H | 0.710 | 0.000 | * |
| LA.F | LDI.H | 0.540 | 0.000 | * |
| LDI.F | LDI.H | 0.890 | 0.000 | * |
| LDI.H | LDMC.F | 0.360 | 0.002 | * |
| LDI.H | MGR.F | -0.050 | 0.710 |  |
| LDI.H | RES(-)T2.F | -0.070 | 0.569 |  |
| LDI.H | SLA.F | -0.020 | 0.851 |  |
| LDI.H | TOL_ASYM.F | 0.050 | 0.656 |  |
| LDI.H | TOL_MGR.F | -0.040 | 0.739 |  |
| LDI.H | TOL_(-)XMID.F | 0.020 | 0.868 |  |
| LDI.H | (-)XMID.F | -0.020 | 0.891 |  |
| ASYM.H | LDI.H | 0.730 | 0.000 | * |
| LA.H | LDI.H | 0.590 | 0.000 | * |
| LDI.H | LDMC.H | 0.380 | 0.001 | * |
| LDI.H | MGR.H | -0.010 | 0.908 |  |
| LDI.H | RES(+)T2.H | -0.100 | 0.425 |  |
| LDI.H | SLA.H | -0.150 | 0.230 |  |
| LDI.H | TOL_ASYM.H | 0.510 | 0.000 | * |

| **Trait_1** | **Trait_2** | **corr.coef** | **p-value** |  |
| --- | --- | --- | --- | --- |
| LDI.H | TOL_MGR.H | 0.130 | 0.305 |  |
| LDI.H | TOL_(-)XMID.H | -0.430 | 0.000 | * |
| LDI.H | (-)XMID.H | -0.340 | 0.004 | * |
| IGR.C | LDI.H | 0.240 | 0.045 | * |
| LDI.H | LTH.C | -0.140 | 0.243 |  |
| LDI.H | NLEA.C | -0.060 | 0.620 |  |
| LDI.H | RES(-)T1.C | -0.130 | 0.305 |  |
| LDI.H | RES(-)T2.C | 0.130 | 0.303 |  |
| LDI.H | RES(+)T1.C | -0.030 | 0.777 |  |
| LDI.H | RES(+)T2.C | 0.020 | 0.867 |  |
| IGR.F | LDI.H | 0.030 | 0.827 |  |
| LDI.H | LTH.F | -0.330 | 0.006 | * |
| LDI.H | NLEA.F | -0.040 | 0.746 |  |
| LDI.H | TOL_IGR.F | -0.220 | 0.065 |  |
| IGR.H | LDI.H | -0.150 | 0.227 |  |
| LDI.H | LTH.H | 0.010 | 0.928 |  |
| LDI.H | NLEA.H | -0.040 | 0.717 |  |
| LDI.H | TOL_IGR.H | -0.050 | 0.703 |  |
| ASYM.C | LDMC.H | 0.440 | 0.000 | * |
| LA.C | LDMC.H | 0.480 | 0.000 | * |
| LDI.C | LDMC.H | 0.340 | 0.004 | * |
| LDMC.C | LDMC.H | 0.790 | 0.000 | * |
| LDMC.H | MGR.C | 0.090 | 0.452 |  |
| LDMC.H | SLA.C | -0.300 | 0.012 | * |
| LDMC.H | SSIZ.C | 0.170 | 0.153 |  |
| LDMC.H | TGER.C | -0.140 | 0.251 |  |
| LDMC.H | (-)XMID.C | 0.080 | 0.509 |  |
| ASYM.F | LDMC.H | 0.510 | 0.000 | * |
| LA.F | LDMC.H | 0.520 | 0.000 | * |
| LDI.F | LDMC.H | 0.320 | 0.007 | * |
| LDMC.F | LDMC.H | 0.730 | 0.000 | * |
| LDMC.H | MGR.F | 0.040 | 0.770 |  |
| LDMC.H | RES(-)T2.F | -0.280 | 0.020 | * |
| LDMC.H | SLA.F | -0.380 | 0.001 | * |
| LDMC.H | TOL_ASYM.F | 0.020 | 0.858 |  |
| LDMC.H | TOL_MGR.F | 0.060 | 0.651 |  |
| LDMC.H | TOL_(-)XMID.F | 0.110 | 0.367 |  |
| LDMC.H | (-)XMID.F | 0.030 | 0.832 |  |
| ASYM.H | LDMC.H | 0.480 | 0.000 | * |
| LA.H | LDMC.H | 0.500 | 0.000 | * |
| LDI.H | LDMC.H | 0.380 | 0.001 | * |
| LDMC.H | MGR.H | -0.030 | 0.817 |  |
| LDMC.H | RES(+)T2.H | 0.220 | 0.067 |  |
| LDMC.H | SLA.H | -0.600 | 0.000 | * |
| LDMC.H | TOL_ASYM.H | 0.340 | 0.004 | * |
| LDMC.H | TOL_MGR.H | 0.080 | 0.498 |  |
| LDMC.H | TOL_(-)XMID.H | -0.240 | 0.046 | * |
| LDMC.H | (-)XMID.H | -0.200 | 0.104 |  |
| IGR.C | LDMC.H | 0.030 | 0.790 |  |

| **Trait_1** | **Trait_2** | **corr.coef** | **p-value** |  |
| --- | --- | --- | --- | --- |
| LDMC.H | LTH.C | -0.120 | 0.344 |  |
| LDMC.H | NLEA.C | -0.220 | 0.067 |  |
| LDMC.H | RES(-)T1.C | -0.200 | 0.104 |  |
| LDMC.H | RES(-)T2.C | 0.120 | 0.321 |  |
| LDMC.H | RES(+)T1.C | 0.030 | 0.813 |  |
| LDMC.H | RES(+)T2.C | 0.350 | 0.003 | * |
| IGR.F | LDMC.H | -0.220 | 0.064 |  |
| LDMC.H | LTH.F | -0.310 | 0.010 | * |
| LDMC.H | NLEA.F | -0.160 | 0.190 |  |
| LDMC.H | TOL_IGR.F | -0.150 | 0.210 |  |
| IGR.H | LDMC.H | 0.010 | 0.932 |  |
| LDMC.H | LTH.H | 0.100 | 0.418 |  |
| LDMC.H | NLEA.H | -0.160 | 0.176 |  |
| LDMC.H | TOL_IGR.H | 0.030 | 0.836 |  |
| ASYM.C | MGR.H | -0.050 | 0.659 |  |
| LA.C | MGR.H | -0.070 | 0.557 |  |
| LDI.C | MGR.H | -0.020 | 0.839 |  |
| LDMC.C | MGR.H | -0.200 | 0.095 |  |
| MGR.C | MGR.H | -0.070 | 0.576 |  |
| MGR.H | SLA.C | 0.040 | 0.752 |  |
| MGR.H | SSIZ.C | -0.070 | 0.552 |  |
| MGR.H | TGER.C | 0.150 | 0.233 |  |
| MGR.H | (-)XMID.C | -0.190 | 0.125 |  |
| ASYM.F | MGR.H | -0.050 | 0.702 |  |
| LA.F | MGR.H | -0.070 | 0.586 |  |
| LDI.F | MGR.H | -0.020 | 0.850 |  |
| LDMC.F | MGR.H | -0.210 | 0.076 |  |
| MGR.F | MGR.H | 0.090 | 0.462 |  |
| MGR.H | RES(-)T2.F | 0.260 | 0.034 | * |
| MGR.H | SLA.F | 0.150 | 0.234 |  |
| MGR.H | TOL_ASYM.F | -0.050 | 0.697 |  |
| MGR.H | TOL_MGR.F | 0.000 | 0.986 |  |
| MGR.H | TOL_(-)XMID.F | 0.030 | 0.826 |  |
| MGR.H | (-)XMID.F | 0.030 | 0.816 |  |
| ASYM.H | MGR.H | -0.090 | 0.449 |  |
| LA.H | MGR.H | -0.080 | 0.502 |  |
| LDI.H | MGR.H | -0.010 | 0.908 |  |
| LDMC.H | MGR.H | -0.030 | 0.817 |  |
| MGR.H | RES(+)T2.H | 0.150 | 0.222 |  |
| MGR.H | SLA.H | 0.000 | 0.969 |  |
| MGR.H | TOL_ASYM.H | -0.210 | 0.087 |  |
| MGR.H | TOL_MGR.H | 0.710 | 0.000 | * |
| MGR.H | TOL_(-)XMID.H | -0.130 | 0.288 |  |
| MGR.H | (-)XMID.H | 0.120 | 0.332 |  |
| IGR.C | MGR.H | -0.060 | 0.619 |  |
| LTH.C | MGR.H | 0.100 | 0.412 |  |
| MGR.H | NLEA.C | -0.160 | 0.195 |  |
| MGR.H | RES(-)T1.C | 0.060 | 0.652 |  |
| MGR.H | RES(-)T2.C | -0.210 | 0.088 |  |

| **Trait_1** | **Trait_2** | **corr.coef** | **p-value** |  |
| --- | --- | --- | --- | --- |
| MGR.H | RES(+)T1.C | -0.020 | 0.851 |  |
| MGR.H | RES(+)T2.C | 0.070 | 0.590 |  |
| IGR.F | MGR.H | -0.130 | 0.280 |  |
| LTH.F | MGR.H | 0.010 | 0.946 |  |
| MGR.H | NLEA.F | -0.110 | 0.356 |  |
| MGR.H | TOL_IGR.F | -0.020 | 0.901 |  |
| IGR.H | MGR.H | 0.030 | 0.789 |  |
| LTH.H | MGR.H | 0.110 | 0.385 |  |
| MGR.H | NLEA.H | -0.200 | 0.102 |  |
| MGR.H | TOL_IGR.H | 0.150 | 0.222 |  |
| ASYM.C | RES(+)T2.H | -0.220 | 0.076 |  |
| LA.C | RES(+)T2.H | -0.190 | 0.126 |  |
| LDI.C | RES(+)T2.H | -0.100 | 0.424 |  |
| LDMC.C | RES(+)T2.H | 0.150 | 0.221 |  |
| MGR.C | RES(+)T2.H | -0.130 | 0.284 |  |
| RES(+)T2.H | SLA.C | 0.030 | 0.820 |  |
| RES(+)T2.H | SSIZ.C | 0.040 | 0.726 |  |
| RES(+)T2.H | TGER.C | 0.180 | 0.132 |  |
| (-)XMID.C | RES(+)T2.H | -0.080 | 0.534 |  |
| ASYM.F | RES(+)T2.H | -0.210 | 0.083 |  |
| LA.F | RES(+)T2.H | -0.210 | 0.089 |  |
| LDI.F | RES(+)T2.H | -0.070 | 0.575 |  |
| LDMC.F | RES(+)T2.H | 0.030 | 0.788 |  |
| MGR.F | RES(+)T2.H | 0.000 | 0.981 |  |
| RES(-)T2.F | RES(+)T2.H | 0.070 | 0.592 |  |
| RES(+)T2.H | SLA.F | 0.060 | 0.651 |  |
| RES(+)T2.H | TOL_ASYM.F | 0.090 | 0.456 |  |
| RES(+)T2.H | TOL_MGR.F | -0.080 | 0.493 |  |
| RES(+)T2.H | TOL_(-)XMID.F | -0.030 | 0.787 |  |
| (-)XMID.F | RES(+)T2.H | 0.080 | 0.494 |  |
| ASYM.H | RES(+)T2.H | -0.280 | 0.019 | * |
| LA.H | RES(+)T2.H | -0.160 | 0.200 |  |
| LDI.H | RES(+)T2.H | -0.100 | 0.425 |  |
| LDMC.H | RES(+)T2.H | 0.220 | 0.067 |  |
| MGR.H | RES(+)T2.H | 0.150 | 0.222 |  |
| RES(+)T2.H | SLA.H | -0.080 | 0.520 |  |
| RES(+)T2.H | TOL_ASYM.H | -0.150 | 0.208 |  |
| RES(+)T2.H | TOL_MGR.H | 0.040 | 0.717 |  |
| RES(+)T2.H | TOL_(-)XMID.H | 0.010 | 0.961 |  |
| (-)XMID.H | RES(+)T2.H | 0.150 | 0.228 |  |
| IGR.C | RES(+)T2.H | 0.070 | 0.585 |  |
| LTH.C | RES(+)T2.H | -0.100 | 0.429 |  |
| NLEA.C | RES(+)T2.H | 0.040 | 0.754 |  |
| RES(-)T1.C | RES(+)T2.H | -0.180 | 0.136 |  |
| RES(-)T2.C | RES(+)T2.H | -0.070 | 0.564 |  |
| RES(+)T1.C | RES(+)T2.H | -0.050 | 0.708 |  |
| RES(+)T2.C | RES(+)T2.H | 0.180 | 0.129 |  |
| IGR.F | RES(+)T2.H | -0.260 | 0.029 | * |
| LTH.F | RES(+)T2.H | -0.040 | 0.736 |  |

| **Trait_1** | **Trait_2** | **corr.coef** | **p-value** |  |
| --- | --- | --- | --- | --- |
| NLEA.F | RES(+)T2.H | -0.040 | 0.748 |  |
| RES(+)T2.H | TOL_IGR.F | -0.070 | 0.573 |  |
| IGR.H | RES(+)T2.H | -0.040 | 0.748 |  |
| LTH.H | RES(+)T2.H | -0.080 | 0.531 |  |
| NLEA.H | RES(+)T2.H | 0.110 | 0.360 |  |
| RES(+)T2.H | TOL_IGR.H | -0.100 | 0.415 |  |
| ASYM.C | SLA.H | -0.390 | 0.001 | * |
| LA.C | SLA.H | -0.520 | 0.000 | * |
| LDI.C | SLA.H | -0.110 | 0.352 |  |
| LDMC.C | SLA.H | -0.510 | 0.000 | * |
| MGR.C | SLA.H | -0.270 | 0.027 | * |
| SLA.C | SLA.H | 0.680 | 0.000 | * |
| SLA.H | SSIZ.C | -0.470 | 0.000 | * |
| SLA.H | TGER.C | 0.040 | 0.766 |  |
| (-)XMID.C | SLA.H | -0.050 | 0.689 |  |
| ASYM.F | SLA.H | -0.460 | 0.000 | * |
| LA.F | SLA.H | -0.560 | 0.000 | * |
| LDI.F | SLA.H | -0.110 | 0.349 |  |
| LDMC.F | SLA.H | -0.430 | 0.000 | * |
| MGR.F | SLA.H | 0.120 | 0.320 |  |
| RES(-)T2.F | SLA.H | 0.230 | 0.062 |  |
| SLA.F | SLA.H | 0.760 | 0.000 | * |
| SLA.H | TOL_ASYM.F | -0.010 | 0.963 |  |
| SLA.H | TOL_MGR.F | 0.270 | 0.025 | * |
| SLA.H | TOL_(-)XMID.F | 0.170 | 0.155 |  |
| (-)XMID.F | SLA.H | 0.170 | 0.161 |  |
| ASYM.H | SLA.H | -0.430 | 0.000 | * |
| LA.H | SLA.H | -0.480 | 0.000 | * |
| LDI.H | SLA.H | -0.150 | 0.230 |  |
| LDMC.H | SLA.H | -0.600 | 0.000 | * |
| MGR.H | SLA.H | 0.000 | 0.969 |  |
| RES(+)T2.H | SLA.H | -0.080 | 0.520 |  |
| SLA.H | TOL_ASYM.H | -0.260 | 0.030 | * |
| SLA.H | TOL_MGR.H | -0.060 | 0.629 |  |
| SLA.H | TOL_(-)XMID.H | 0.130 | 0.275 |  |
| (-)XMID.H | SLA.H | 0.190 | 0.125 |  |
| IGR.C | SLA.H | 0.010 | 0.960 |  |
| LTH.C | SLA.H | -0.250 | 0.040 | * |
| NLEA.C | SLA.H | 0.530 | 0.000 | * |
| RES(-)T1.C | SLA.H | -0.230 | 0.059 |  |
| RES(-)T2.C | SLA.H | 0.030 | 0.797 |  |
| RES(+)T1.C | SLA.H | -0.280 | 0.018 | * |
| RES(+)T2.C | SLA.H | -0.080 | 0.522 |  |
| IGR.F | SLA.H | -0.010 | 0.921 |  |
| LTH.F | SLA.H | -0.150 | 0.226 |  |
| NLEA.F | SLA.H | 0.470 | 0.000 | * |
| SLA.H | TOL_IGR.F | 0.060 | 0.646 |  |
| IGR.H | SLA.H | -0.090 | 0.440 |  |
| LTH.H | SLA.H | -0.240 | 0.046 | * |

| **Trait_1** | **Trait_2** | **corr.coef** | **p-value** |  |
| --- | --- | --- | --- | --- |
| NLEA.H | SLA.H | 0.490 | 0.000 | * |
| SLA.H | TOL_IGR.H | -0.020 | 0.892 |  |
| ASYM.C | TOL_ASYM.H | 0.570 | 0.000 | * |
| LA.C | TOL_ASYM.H | 0.560 | 0.000 | * |
| LDI.C | TOL_ASYM.H | 0.430 | 0.000 | * |
| LDMC.C | TOL_ASYM.H | 0.350 | 0.003 | * |
| MGR.C | TOL_ASYM.H | 0.150 | 0.209 |  |
| SLA.C | TOL_ASYM.H | -0.060 | 0.639 |  |
| SSIZ.C | TOL_ASYM.H | 0.050 | 0.686 |  |
| TGER.C | TOL_ASYM.H | -0.310 | 0.009 | * |
| (-)XMID.C | TOL_ASYM.H | -0.020 | 0.886 |  |
| ASYM.F | TOL_ASYM.H | 0.610 | 0.000 | * |
| LA.F | TOL_ASYM.H | 0.540 | 0.000 | * |
| LDI.F | TOL_ASYM.H | 0.390 | 0.001 | * |
| LDMC.F | TOL_ASYM.H | 0.380 | 0.001 | * |
| MGR.F | TOL_ASYM.H | 0.000 | 0.992 |  |
| RES(-)T2.F | TOL_ASYM.H | -0.280 | 0.021 | * |
| SLA.F | TOL_ASYM.H | -0.110 | 0.389 |  |
| TOL_ASYM.F | TOL_ASYM.H | 0.060 | 0.600 |  |
| TOL_ASYM.H | TOL_MGR.F | -0.090 | 0.468 |  |
| TOL_ASYM.H | TOL_(-)XMID.F | -0.090 | 0.480 |  |
| (-)XMID.F | TOL_ASYM.H | -0.150 | 0.230 |  |
| ASYM.H | TOL_ASYM.H | 0.700 | 0.000 | * |
| LA.H | TOL_ASYM.H | 0.600 | 0.000 | * |
| LDI.H | TOL_ASYM.H | 0.510 | 0.000 | * |
| LDMC.H | TOL_ASYM.H | 0.340 | 0.004 | * |
| MGR.H | TOL_ASYM.H | -0.210 | 0.087 |  |
| RES(+)T2.H | TOL_ASYM.H | -0.150 | 0.208 |  |
| SLA.H | TOL_ASYM.H | -0.260 | 0.030 | * |
| TOL_ASYM.H | TOL_MGR.H | -0.020 | 0.898 |  |
| TOL_ASYM.H | TOL_(-)XMID.H | -0.440 | 0.000 | * |
| (-)XMID.H | TOL_ASYM.H | -0.470 | 0.000 | * |
| IGR.C | TOL_ASYM.H | 0.320 | 0.007 | * |
| LTH.C | TOL_ASYM.H | -0.080 | 0.506 |  |
| NLEA.C | TOL_ASYM.H | -0.140 | 0.255 |  |
| RES(-)T1.C | TOL_ASYM.H | -0.030 | 0.838 |  |
| RES(-)T2.C | TOL_ASYM.H | 0.280 | 0.022 | * |
| RES(+)T1.C | TOL_ASYM.H | 0.080 | 0.515 |  |
| RES(+)T2.C | TOL_ASYM.H | 0.210 | 0.090 |  |
| IGR.F | TOL_ASYM.H | 0.190 | 0.118 |  |
| LTH.F | TOL_ASYM.H | -0.150 | 0.217 |  |
| NLEA.F | TOL_ASYM.H | -0.160 | 0.200 |  |
| TOL_ASYM.H | TOL_IGR.F | -0.240 | 0.044 | * |
| IGR.H | TOL_ASYM.H | -0.020 | 0.898 |  |
| LTH.H | TOL_ASYM.H | 0.090 | 0.483 |  |
| NLEA.H | TOL_ASYM.H | -0.070 | 0.557 |  |
| TOL_ASYM.H | TOL_IGR.H | 0.060 | 0.603 |  |
| ASYM.C | TOL_MGR.H | 0.120 | 0.318 |  |
| LA.C | TOL_MGR.H | 0.070 | 0.546 |  |

| **Trait_1** | **Trait_2** | **corr.coef** | **p-value** |  |
| --- | --- | --- | --- | --- |
| LDI.C | TOL_MGR.H | 0.170 | 0.155 |  |
| LDMC.C | TOL_MGR.H | -0.130 | 0.304 |  |
| MGR.C | TOL_MGR.H | -0.190 | 0.128 |  |
| SLA.C | TOL_MGR.H | 0.020 | 0.886 |  |
| SSIZ.C | TOL_MGR.H | 0.010 | 0.954 |  |
| TGER.C | TOL_MGR.H | -0.010 | 0.959 |  |
| (-)XMID.C | TOL_MGR.H | -0.180 | 0.128 |  |
| ASYM.F | TOL_MGR.H | 0.100 | 0.402 |  |
| LA.F | TOL_MGR.H | 0.050 | 0.663 |  |
| LDI.F | TOL_MGR.H | 0.180 | 0.150 |  |
| LDMC.F | TOL_MGR.H | -0.060 | 0.600 |  |
| MGR.F | TOL_MGR.H | 0.100 | 0.434 |  |
| RES(-)T2.F | TOL_MGR.H | 0.050 | 0.664 |  |
| SLA.F | TOL_MGR.H | 0.070 | 0.563 |  |
| TOL_ASYM.F | TOL_MGR.H | -0.130 | 0.292 |  |
| TOL_MGR.F | TOL_MGR.H | 0.100 | 0.432 |  |
| TOL_MGR.H | TOL_(-)XMID.F | 0.130 | 0.296 |  |
| (-)XMID.F | TOL_MGR.H | -0.100 | 0.433 |  |
| ASYM.H | TOL_MGR.H | 0.030 | 0.794 |  |
| LA.H | TOL_MGR.H | 0.030 | 0.783 |  |
| LDI.H | TOL_MGR.H | 0.130 | 0.305 |  |
| LDMC.H | TOL_MGR.H | 0.080 | 0.498 |  |
| MGR.H | TOL_MGR.H | 0.710 | 0.000 | * |
| RES(+)T2.H | TOL_MGR.H | 0.040 | 0.717 |  |
| SLA.H | TOL_MGR.H | -0.060 | 0.629 |  |
| TOL_ASYM.H | TOL_MGR.H | -0.020 | 0.898 |  |
| TOL_MGR.H | TOL_(-)XMID.H | -0.180 | 0.128 |  |
| (-)XMID.H | TOL_MGR.H | -0.080 | 0.518 |  |
| IGR.C | TOL_MGR.H | 0.120 | 0.333 |  |
| LTH.C | TOL_MGR.H | 0.180 | 0.147 |  |
| NLEA.C | TOL_MGR.H | -0.110 | 0.359 |  |
| RES(-)T1.C | TOL_MGR.H | 0.080 | 0.506 |  |
| RES(-)T2.C | TOL_MGR.H | -0.130 | 0.269 |  |
| RES(+)T1.C | TOL_MGR.H | -0.110 | 0.356 |  |
| RES(+)T2.C | TOL_MGR.H | 0.010 | 0.925 |  |
| IGR.F | TOL_MGR.H | -0.100 | 0.408 |  |
| LTH.F | TOL_MGR.H | 0.070 | 0.550 |  |
| NLEA.F | TOL_MGR.H | -0.080 | 0.515 |  |
| TOL_IGR.F | TOL_MGR.H | -0.190 | 0.111 |  |
| IGR.H | TOL_MGR.H | -0.040 | 0.752 |  |
| LTH.H | TOL_MGR.H | 0.210 | 0.087 |  |
| NLEA.H | TOL_MGR.H | -0.220 | 0.075 |  |
| TOL_IGR.H | TOL_MGR.H | 0.070 | 0.593 |  |
| ASYM.C | TOL_(-)XMID.H | -0.280 | 0.021 | * |
| LA.C | TOL_(-)XMID.H | -0.360 | 0.003 | * |
| LDI.C | TOL_(-)XMID.H | -0.330 | 0.006 | * |
| LDMC.C | TOL_(-)XMID.H | -0.140 | 0.258 |  |
| MGR.C | TOL_(-)XMID.H | 0.030 | 0.836 |  |
| SLA.C | TOL_(-)XMID.H | -0.100 | 0.404 |  |

| **Trait_1** | **Trait_2** | **corr.coef** | **p-value** |  |
| --- | --- | --- | --- | --- |
| SSIZ.C | TOL_(-)XMID.H | 0.080 | 0.506 |  |
| TGER.C | TOL_(-)XMID.H | 0.050 | 0.710 |  |
| (-)XMID.C | TOL_(-)XMID.H | 0.170 | 0.162 |  |
| ASYM.F | TOL_(-)XMID.H | -0.320 | 0.007 | * |
| LA.F | TOL_(-)XMID.H | -0.310 | 0.009 | * |
| LDI.F | TOL_(-)XMID.H | -0.260 | 0.032 | * |
| LDMC.F | TOL_(-)XMID.H | -0.160 | 0.181 |  |
| MGR.F | TOL_(-)XMID.H | 0.030 | 0.789 |  |
| RES(-)T2.F | TOL_(-)XMID.H | -0.010 | 0.955 |  |
| SLA.F | TOL_(-)XMID.H | 0.030 | 0.822 |  |
| TOL_ASYM.F | TOL_(-)XMID.H | -0.040 | 0.737 |  |
| TOL_MGR.F | TOL_(-)XMID.H | 0.030 | 0.827 |  |
| TOL_(-)XMID.F | TOL_(-)XMID.H | -0.160 | 0.182 |  |
| (-)XMID.F | TOL_(-)XMID.H | 0.040 | 0.723 |  |
| ASYM.H | TOL_(-)XMID.H | -0.380 | 0.001 | * |
| LA.H | TOL_(-)XMID.H | -0.330 | 0.005 | * |
| LDI.H | TOL_(-)XMID.H | -0.430 | 0.000 | * |
| LDMC.H | TOL_(-)XMID.H | -0.240 | 0.046 | * |
| MGR.H | TOL_(-)XMID.H | -0.130 | 0.288 |  |
| RES(+)T2.H | TOL_(-)XMID.H | 0.010 | 0.961 |  |
| SLA.H | TOL_(-)XMID.H | 0.130 | 0.275 |  |
| TOL_ASYM.H | TOL_(-)XMID.H | -0.440 | 0.000 | * |
| TOL_MGR.H | TOL_(-)XMID.H | -0.180 | 0.128 |  |
| (-)XMID.H | TOL_(-)XMID.H | 0.530 | 0.000 | * |
| IGR.C | TOL_(-)XMID.H | -0.080 | 0.504 |  |
| LTH.C | TOL_(-)XMID.H | 0.100 | 0.409 |  |
| NLEA.C | TOL_(-)XMID.H | 0.210 | 0.083 |  |
| RES(-)T1.C | TOL_(-)XMID.H | -0.170 | 0.161 |  |
| RES(-)T2.C | TOL_(-)XMID.H | 0.190 | 0.109 |  |
| RES(+)T1.C | TOL_(-)XMID.H | -0.080 | 0.510 |  |
| RES(+)T2.C | TOL_(-)XMID.H | -0.240 | 0.051 |  |
| IGR.F | TOL_(-)XMID.H | -0.090 | 0.485 |  |
| LTH.F | TOL_(-)XMID.H | 0.210 | 0.079 |  |
| NLEA.F | TOL_(-)XMID.H | 0.100 | 0.404 |  |
| TOL_IGR.F | TOL_(-)XMID.H | 0.060 | 0.596 |  |
| IGR.H | TOL_(-)XMID.H | 0.080 | 0.523 |  |
| LTH.H | TOL_(-)XMID.H | 0.010 | 0.929 |  |
| NLEA.H | TOL_(-)XMID.H | 0.240 | 0.048 | * |
| TOL_IGR.H | TOL_(-)XMID.H | 0.040 | 0.724 |  |
| ASYM.C | (-)XMID.H | -0.320 | 0.007 | * |
| LA.C | (-)XMID.H | -0.360 | 0.003 | * |
| LDI.C | (-)XMID.H | -0.230 | 0.058 |  |
| LDMC.C | (-)XMID.H | -0.220 | 0.064 |  |
| MGR.C | (-)XMID.H | -0.150 | 0.226 |  |
| (-)XMID.H | SLA.C | 0.220 | 0.064 |  |
| (-)XMID.H | SSIZ.C | 0.190 | 0.109 |  |
| (-)XMID.H | TGER.C | 0.430 | 0.000 | * |
| (-)XMID.C | (-)XMID.H | 0.430 | 0.000 | * |
| ASYM.F | (-)XMID.H | -0.350 | 0.003 | * |

| **Trait_1** | **Trait_2** | **corr.coef** | **p-value** |  |
| --- | --- | --- | --- | --- |
| LA.F | (-)XMID.H | -0.290 | 0.017 | * |
| LDI.F | (-)XMID.H | -0.210 | 0.080 |  |
| LDMC.F | (-)XMID.H | -0.240 | 0.044 | * |
| MGR.F | (-)XMID.H | 0.160 | 0.199 |  |
| (-)XMID.H | RES(-)T2.F | 0.340 | 0.004 | * |
| (-)XMID.H | SLA.F | 0.190 | 0.123 |  |
| (-)XMID.H | TOL_ASYM.F | 0.110 | 0.385 |  |
| (-)XMID.H | TOL_MGR.F | 0.010 | 0.917 |  |
| (-)XMID.H | TOL_(-)XMID.F | -0.190 | 0.126 |  |
| (-)XMID.F | (-)XMID.H | 0.470 | 0.000 | * |
| ASYM.H | (-)XMID.H | -0.380 | 0.001 | * |
| LA.H | (-)XMID.H | -0.310 | 0.010 | * |
| LDI.H | (-)XMID.H | -0.340 | 0.004 | * |
| LDMC.H | (-)XMID.H | -0.200 | 0.104 |  |
| MGR.H | (-)XMID.H | 0.120 | 0.332 |  |
| (-)XMID.H | RES(+)T2.H | 0.150 | 0.228 |  |
| (-)XMID.H | SLA.H | 0.190 | 0.125 |  |
| (-)XMID.H | TOL_ASYM.H | -0.470 | 0.000 | * |
| (-)XMID.H | TOL_MGR.H | -0.080 | 0.518 |  |
| (-)XMID.H | TOL_(-)XMID.H | 0.530 | 0.000 | * |
| IGR.C | (-)XMID.H | -0.140 | 0.242 |  |
| LTH.C | (-)XMID.H | -0.170 | 0.157 |  |
| (-)XMID.H | NLEA.C | 0.030 | 0.823 |  |
| (-)XMID.H | RES(-)T1.C | -0.160 | 0.189 |  |
| (-)XMID.H | RES(-)T2.C | 0.070 | 0.581 |  |
| (-)XMID.H | RES(+)T1.C | -0.080 | 0.526 |  |
| (-)XMID.H | RES(+)T2.C | -0.180 | 0.137 |  |
| IGR.F | (-)XMID.H | -0.280 | 0.019 | * |
| LTH.F | (-)XMID.H | 0.000 | 0.988 |  |
| (-)XMID.H | NLEA.F | 0.000 | 0.990 |  |
| (-)XMID.H | TOL_IGR.F | -0.020 | 0.860 |  |
| IGR.H | (-)XMID.H | 0.010 | 0.942 |  |
| LTH.H | (-)XMID.H | 0.020 | 0.859 |  |
| (-)XMID.H | NLEA.H | 0.100 | 0.416 |  |
| (-)XMID.H | TOL_IGR.H | 0.130 | 0.291 |  |
| ASYM.C | IGR.C | 0.260 | 0.033 | * |
| IGR.C | LA.C | 0.250 | 0.041 | * |
| IGR.C | LDI.C | 0.200 | 0.108 |  |
| IGR.C | LDMC.C | 0.130 | 0.292 |  |
| IGR.C | MGR.C | 0.030 | 0.827 |  |
| IGR.C | SLA.C | -0.080 | 0.506 |  |
| IGR.C | SSIZ.C | 0.150 | 0.232 |  |
| IGR.C | TGER.C | -0.170 | 0.175 |  |
| IGR.C | (-)XMID.C | 0.030 | 0.787 |  |
| ASYM.F | IGR.C | 0.300 | 0.012 | * |
| IGR.C | LA.F | 0.230 | 0.060 |  |
| IGR.C | LDI.F | 0.240 | 0.046 | * |
| IGR.C | LDMC.F | 0.030 | 0.831 |  |
| IGR.C | MGR.F | 0.130 | 0.296 |  |

| **Trait_1** | **Trait_2** | **corr.coef** | **p-value** |  |
| --- | --- | --- | --- | --- |
| IGR.C | RES(-)T2.F | -0.100 | 0.409 |  |
| IGR.C | SLA.F | -0.030 | 0.838 |  |
| IGR.C | TOL_ASYM.F | 0.170 | 0.161 |  |
| IGR.C | TOL_MGR.F | -0.070 | 0.554 |  |
| IGR.C | TOL_(-)XMID.F | -0.030 | 0.835 |  |
| IGR.C | (-)XMID.F | -0.130 | 0.276 |  |
| ASYM.H | IGR.C | 0.230 | 0.058 |  |
| IGR.C | LA.H | 0.290 | 0.014 | * |
| IGR.C | LDI.H | 0.240 | 0.045 | * |
| IGR.C | LDMC.H | 0.030 | 0.790 |  |
| IGR.C | MGR.H | -0.060 | 0.619 |  |
| IGR.C | RES(+)T2.H | 0.070 | 0.585 |  |
| IGR.C | SLA.H | 0.010 | 0.960 |  |
| IGR.C | TOL_ASYM.H | 0.320 | 0.007 | * |
| IGR.C | TOL_MGR.H | 0.120 | 0.333 |  |
| IGR.C | TOL_(-)XMID.H | -0.080 | 0.504 |  |
| IGR.C | (-)XMID.H | -0.140 | 0.242 |  |
| IGR.C | LTH.C | 0.090 | 0.446 |  |
| IGR.C | NLEA.C | 0.020 | 0.845 |  |
| IGR.C | RES(-)T1.C | 0.010 | 0.908 |  |
| IGR.C | RES(-)T2.C | 0.230 | 0.056 |  |
| IGR.C | RES(+)T1.C | 0.160 | 0.197 |  |
| IGR.C | RES(+)T2.C | -0.140 | 0.242 |  |
| IGR.C | IGR.F | -0.030 | 0.794 |  |
| IGR.C | LTH.F | 0.180 | 0.143 |  |
| IGR.C | NLEA.F | -0.070 | 0.564 |  |
| IGR.C | TOL_IGR.F | -0.730 | 0.000 | * |
| IGR.C | IGR.H | -0.080 | 0.530 |  |
| IGR.C | LTH.H | 0.120 | 0.333 |  |
| IGR.C | NLEA.H | 0.030 | 0.816 |  |
| IGR.C | TOL_IGR.H | -0.130 | 0.284 |  |
| ASYM.C | LTH.C | 0.120 | 0.324 |  |
| LA.C | LTH.C | 0.180 | 0.141 |  |
| LDI.C | LTH.C | -0.260 | 0.034 | * |
| LDMC.C | LTH.C | 0.050 | 0.706 |  |
| LTH.C | MGR.C | 0.060 | 0.649 |  |
| LTH.C | SLA.C | -0.560 | 0.000 | * |
| LTH.C | SSIZ.C | 0.400 | 0.001 | * |
| LTH.C | TGER.C | -0.150 | 0.222 |  |
| LTH.C | (-)XMID.C | 0.060 | 0.644 |  |
| ASYM.F | LTH.C | 0.120 | 0.332 |  |
| LA.F | LTH.C | 0.190 | 0.110 |  |
| LDI.F | LTH.C | -0.210 | 0.081 |  |
| LDMC.F | LTH.C | -0.080 | 0.502 |  |
| LTH.C | MGR.F | -0.010 | 0.947 |  |
| LTH.C | RES(-)T2.F | -0.160 | 0.181 |  |
| LTH.C | SLA.F | -0.470 | 0.000 | * |
| LTH.C | TOL_ASYM.F | 0.010 | 0.945 |  |
| LTH.C | TOL_MGR.F | -0.020 | 0.881 |  |

| **Trait_1** | **Trait_2** | **corr.coef** | **p-value** |  |
| --- | --- | --- | --- | --- |
| LTH.C | TOL_(-)XMID.F | -0.080 | 0.526 |  |
| LTH.C | (-)XMID.F | -0.040 | 0.727 |  |
| ASYM.H | LTH.C | 0.050 | 0.687 |  |
| LA.H | LTH.C | 0.180 | 0.133 |  |
| LDI.H | LTH.C | -0.140 | 0.243 |  |
| LDMC.H | LTH.C | -0.120 | 0.344 |  |
| LTH.C | MGR.H | 0.100 | 0.412 |  |
| LTH.C | RES(+)T2.H | -0.100 | 0.429 |  |
| LTH.C | SLA.H | -0.250 | 0.040 | * |
| LTH.C | TOL_ASYM.H | -0.080 | 0.506 |  |
| LTH.C | TOL_MGR.H | 0.180 | 0.147 |  |
| LTH.C | TOL_(-)XMID.H | 0.100 | 0.409 |  |
| LTH.C | (-)XMID.H | -0.170 | 0.157 |  |
| IGR.C | LTH.C | 0.090 | 0.446 |  |
| LTH.C | NLEA.C | -0.330 | 0.005 | * |
| LTH.C | RES(-)T1.C | 0.510 | 0.000 | * |
| LTH.C | RES(-)T2.C | -0.030 | 0.780 |  |
| LTH.C | RES(+)T1.C | 0.400 | 0.001 | * |
| LTH.C | RES(+)T2.C | -0.160 | 0.183 |  |
| IGR.F | LTH.C | 0.030 | 0.794 |  |
| LTH.C | LTH.F | 0.560 | 0.000 | * |
| LTH.C | NLEA.F | -0.290 | 0.017 | * |
| LTH.C | TOL_IGR.F | -0.070 | 0.559 |  |
| IGR.H | LTH.C | 0.100 | 0.411 |  |
| LTH.C | LTH.H | 0.110 | 0.390 |  |
| LTH.C | NLEA.H | -0.410 | 0.000 | * |
| LTH.C | TOL_IGR.H | -0.090 | 0.475 |  |
| ASYM.C | NLEA.C | -0.320 | 0.007 | * |
| LA.C | NLEA.C | -0.450 | 0.000 | * |
| LDI.C | NLEA.C | -0.020 | 0.845 |  |
| LDMC.C | NLEA.C | -0.180 | 0.147 |  |
| MGR.C | NLEA.C | 0.040 | 0.739 |  |
| NLEA.C | SLA.C | 0.410 | 0.000 | * |
| NLEA.C | SSIZ.C | -0.430 | 0.000 | * |
| NLEA.C | TGER.C | -0.310 | 0.009 | * |
| (-)XMID.C | NLEA.C | -0.150 | 0.204 |  |
| ASYM.F | NLEA.C | -0.320 | 0.008 | * |
| LA.F | NLEA.C | -0.490 | 0.000 | * |
| LDI.F | NLEA.C | -0.030 | 0.835 |  |
| LDMC.F | NLEA.C | -0.030 | 0.791 |  |
| MGR.F | NLEA.C | 0.110 | 0.382 |  |
| NLEA.C | RES(-)T2.F | 0.000 | 0.983 |  |
| NLEA.C | SLA.F | 0.430 | 0.000 | * |
| NLEA.C | TOL_ASYM.F | 0.090 | 0.447 |  |
| NLEA.C | TOL_MGR.F | 0.000 | 0.982 |  |
| NLEA.C | TOL_(-)XMID.F | 0.040 | 0.731 |  |
| (-)XMID.F | NLEA.C | -0.020 | 0.865 |  |
| ASYM.H | NLEA.C | -0.360 | 0.003 | * |
| LA.H | NLEA.C | -0.470 | 0.000 | * |

| **Trait_1** | **Trait_2** | **corr.coef** | **p-value** |  |
| --- | --- | --- | --- | --- |
| LDI.H | NLEA.C | -0.060 | 0.620 |  |
| LDMC.H | NLEA.C | -0.220 | 0.067 |  |
| MGR.H | NLEA.C | -0.160 | 0.195 |  |
| NLEA.C | RES(+)T2.H | 0.040 | 0.754 |  |
| NLEA.C | SLA.H | 0.530 | 0.000 | * |
| NLEA.C | TOL_ASYM.H | -0.140 | 0.255 |  |
| NLEA.C | TOL_MGR.H | -0.110 | 0.359 |  |
| NLEA.C | TOL_(-)XMID.H | 0.210 | 0.083 |  |
| (-)XMID.H | NLEA.C | 0.030 | 0.823 |  |
| IGR.C | NLEA.C | 0.020 | 0.845 |  |
| LTH.C | NLEA.C | -0.330 | 0.005 | * |
| NLEA.C | RES(-)T1.C | -0.280 | 0.019 | * |
| NLEA.C | RES(-)T2.C | 0.130 | 0.279 |  |
| NLEA.C | RES(+)T1.C | -0.220 | 0.070 |  |
| NLEA.C | RES(+)T2.C | 0.010 | 0.938 |  |
| IGR.F | NLEA.C | -0.150 | 0.207 |  |
| LTH.F | NLEA.C | -0.160 | 0.186 |  |
| NLEA.C | NLEA.F | 0.870 | 0.000 | * |
| NLEA.C | TOL_IGR.F | -0.020 | 0.886 |  |
| IGR.H | NLEA.C | -0.190 | 0.127 |  |
| LTH.H | NLEA.C | -0.240 | 0.044 | * |
| NLEA.C | NLEA.H | 0.920 | 0.000 | * |
| NLEA.C | TOL_IGR.H | -0.130 | 0.276 |  |
| ASYM.C | RES(-)T1.C | 0.050 | 0.697 |  |
| LA.C | RES(-)T1.C | 0.180 | 0.146 |  |
| LDI.C | RES(-)T1.C | -0.190 | 0.109 |  |
| LDMC.C | RES(-)T1.C | -0.090 | 0.454 |  |
| MGR.C | RES(-)T1.C | 0.080 | 0.498 |  |
| RES(-)T1.C | SLA.C | -0.370 | 0.002 | * |
| RES(-)T1.C | SSIZ.C | 0.270 | 0.026 | * |
| RES(-)T1.C | TGER.C | 0.000 | 0.990 |  |
| (-)XMID.C | RES(-)T1.C | 0.080 | 0.504 |  |
| ASYM.F | RES(-)T1.C | 0.050 | 0.669 |  |
| LA.F | RES(-)T1.C | 0.170 | 0.153 |  |
| LDI.F | RES(-)T1.C | -0.210 | 0.084 |  |
| LDMC.F | RES(-)T1.C | -0.210 | 0.089 |  |
| MGR.F | RES(-)T1.C | -0.120 | 0.327 |  |
| RES(-)T1.C | RES(-)T2.F | 0.220 | 0.066 |  |
| RES(-)T1.C | SLA.F | -0.410 | 0.001 | * |
| RES(-)T1.C | TOL_ASYM.F | -0.100 | 0.424 |  |
| RES(-)T1.C | TOL_MGR.F | -0.190 | 0.113 |  |
| RES(-)T1.C | TOL_(-)XMID.F | -0.120 | 0.317 |  |
| (-)XMID.F | RES(-)T1.C | -0.010 | 0.910 |  |
| ASYM.H | RES(-)T1.C | 0.080 | 0.494 |  |
| LA.H | RES(-)T1.C | 0.140 | 0.251 |  |
| LDI.H | RES(-)T1.C | -0.130 | 0.305 |  |
| LDMC.H | RES(-)T1.C | -0.200 | 0.104 |  |
| MGR.H | RES(-)T1.C | 0.060 | 0.652 |  |
| RES(-)T1.C | RES(+)T2.H | -0.180 | 0.136 |  |

| **Trait_1** | **Trait_2** | **corr.coef** | **p-value** |  |
| --- | --- | --- | --- | --- |
| RES(-)T1.C | SLA.H | -0.230 | 0.059 |  |
| RES(-)T1.C | TOL_ASYM.H | -0.030 | 0.838 |  |
| RES(-)T1.C | TOL_MGR.H | 0.080 | 0.506 |  |
| RES(-)T1.C | TOL_(-)XMID.H | -0.170 | 0.161 |  |
| (-)XMID.H | RES(-)T1.C | -0.160 | 0.189 |  |
| IGR.C | RES(-)T1.C | 0.010 | 0.908 |  |
| LTH.C | RES(-)T1.C | 0.510 | 0.000 | * |
| NLEA.C | RES(-)T1.C | -0.280 | 0.019 | * |
| RES(-)T1.C | RES(-)T2.C | 0.030 | 0.799 |  |
| RES(-)T1.C | RES(+)T1.C | 0.570 | 0.000 | * |
| RES(-)T1.C | RES(+)T2.C | -0.040 | 0.738 |  |
| IGR.F | RES(-)T1.C | 0.260 | 0.031 | * |
| LTH.F | RES(-)T1.C | 0.490 | 0.000 | * |
| NLEA.F | RES(-)T1.C | -0.230 | 0.058 |  |
| RES(-)T1.C | TOL_IGR.F | 0.090 | 0.454 |  |
| IGR.H | RES(-)T1.C | 0.160 | 0.182 |  |
| LTH.H | RES(-)T1.C | 0.250 | 0.038 | * |
| NLEA.H | RES(-)T1.C | -0.330 | 0.006 | * |
| RES(-)T1.C | TOL_IGR.H | -0.010 | 0.918 |  |
| ASYM.C | RES(-)T2.C | 0.270 | 0.022 | * |
| LA.C | RES(-)T2.C | 0.160 | 0.195 |  |
| LDI.C | RES(-)T2.C | 0.140 | 0.266 |  |
| LDMC.C | RES(-)T2.C | 0.200 | 0.095 |  |
| MGR.C | RES(-)T2.C | 0.020 | 0.890 |  |
| RES(-)T2.C | SLA.C | -0.100 | 0.410 |  |
| RES(-)T2.C | SSIZ.C | -0.030 | 0.806 |  |
| RES(-)T2.C | TGER.C | -0.320 | 0.007 | * |
| (-)XMID.C | RES(-)T2.C | 0.040 | 0.735 |  |
| ASYM.F | RES(-)T2.C | 0.260 | 0.033 | * |
| LA.F | RES(-)T2.C | 0.160 | 0.190 |  |
| LDI.F | RES(-)T2.C | 0.200 | 0.104 |  |
| LDMC.F | RES(-)T2.C | 0.110 | 0.368 |  |
| MGR.F | RES(-)T2.C | 0.070 | 0.573 |  |
| RES(-)T2.C | RES(-)T2.F | 0.110 | 0.377 |  |
| RES(-)T2.C | SLA.F | -0.070 | 0.579 |  |
| RES(-)T2.C | TOL_ASYM.F | -0.070 | 0.566 |  |
| RES(-)T2.C | TOL_MGR.F | 0.060 | 0.629 |  |
| RES(-)T2.C | TOL_(-)XMID.F | -0.150 | 0.211 |  |
| (-)XMID.F | RES(-)T2.C | -0.040 | 0.720 |  |
| ASYM.H | RES(-)T2.C | 0.210 | 0.088 |  |
| LA.H | RES(-)T2.C | 0.160 | 0.198 |  |
| LDI.H | RES(-)T2.C | 0.130 | 0.303 |  |
| LDMC.H | RES(-)T2.C | 0.120 | 0.321 |  |
| MGR.H | RES(-)T2.C | -0.210 | 0.088 |  |
| RES(-)T2.C | RES(+)T2.H | -0.070 | 0.564 |  |
| RES(-)T2.C | SLA.H | 0.030 | 0.797 |  |
| RES(-)T2.C | TOL_ASYM.H | 0.280 | 0.022 | * |
| RES(-)T2.C | TOL_MGR.H | -0.130 | 0.269 |  |
| RES(-)T2.C | TOL_(-)XMID.H | 0.190 | 0.109 |  |

| **Trait_1** | **Trait_2** | **corr.coef** | **p-value** |  |
| --- | --- | --- | --- | --- |
| (-)XMID.H | RES(-)T2.C | 0.070 | 0.581 |  |
| IGR.C | RES(-)T2.C | 0.230 | 0.056 |  |
| LTH.C | RES(-)T2.C | -0.030 | 0.780 |  |
| NLEA.C | RES(-)T2.C | 0.130 | 0.279 |  |
| RES(-)T1.C | RES(-)T2.C | 0.030 | 0.799 |  |
| RES(-)T2.C | RES(+)T1.C | 0.170 | 0.153 |  |
| RES(-)T2.C | RES(+)T2.C | -0.110 | 0.369 |  |
| IGR.F | RES(-)T2.C | -0.040 | 0.719 |  |
| LTH.F | RES(-)T2.C | 0.050 | 0.656 |  |
| NLEA.F | RES(-)T2.C | 0.100 | 0.412 |  |
| RES(-)T2.C | TOL_IGR.F | -0.190 | 0.126 |  |
| IGR.H | RES(-)T2.C | 0.290 | 0.015 | * |
| LTH.H | RES(-)T2.C | -0.100 | 0.434 |  |
| NLEA.H | RES(-)T2.C | 0.140 | 0.238 |  |
| RES(-)T2.C | TOL_IGR.H | 0.180 | 0.143 |  |
| ASYM.C | RES(+)T1.C | 0.200 | 0.104 |  |
| LA.C | RES(+)T1.C | 0.210 | 0.091 |  |
| LDI.C | RES(+)T1.C | -0.110 | 0.353 |  |
| LDMC.C | RES(+)T1.C | 0.190 | 0.128 |  |
| MGR.C | RES(+)T1.C | 0.180 | 0.142 |  |
| RES(+)T1.C | SLA.C | -0.460 | 0.000 | * |
| RES(+)T1.C | SSIZ.C | 0.370 | 0.002 | * |
| RES(+)T1.C | TGER.C | -0.110 | 0.362 |  |
| (-)XMID.C | RES(+)T1.C | 0.000 | 0.998 |  |
| ASYM.F | RES(+)T1.C | 0.190 | 0.115 |  |
| LA.F | RES(+)T1.C | 0.220 | 0.066 |  |
| LDI.F | RES(+)T1.C | -0.150 | 0.214 |  |
| LDMC.F | RES(+)T1.C | 0.100 | 0.415 |  |
| MGR.F | RES(+)T1.C | -0.070 | 0.587 |  |
| RES(-)T2.F | RES(+)T1.C | -0.010 | 0.925 |  |
| RES(+)T1.C | SLA.F | -0.490 | 0.000 | * |
| RES(+)T1.C | TOL_ASYM.F | -0.110 | 0.364 |  |
| RES(+)T1.C | TOL_MGR.F | -0.070 | 0.553 |  |
| RES(+)T1.C | TOL_(-)XMID.F | -0.160 | 0.180 |  |
| (-)XMID.F | RES(+)T1.C | -0.030 | 0.805 |  |
| ASYM.H | RES(+)T1.C | 0.210 | 0.079 |  |
| LA.H | RES(+)T1.C | 0.290 | 0.016 | * |
| LDI.H | RES(+)T1.C | -0.030 | 0.777 |  |
| LDMC.H | RES(+)T1.C | 0.030 | 0.813 |  |
| MGR.H | RES(+)T1.C | -0.020 | 0.851 |  |
| RES(+)T1.C | RES(+)T2.H | -0.050 | 0.708 |  |
| RES(+)T1.C | SLA.H | -0.280 | 0.018 | * |
| RES(+)T1.C | TOL_ASYM.H | 0.080 | 0.515 |  |
| RES(+)T1.C | TOL_MGR.H | -0.110 | 0.356 |  |
| RES(+)T1.C | TOL_(-)XMID.H | -0.080 | 0.510 |  |
| (-)XMID.H | RES(+)T1.C | -0.080 | 0.526 |  |
| IGR.C | RES(+)T1.C | 0.160 | 0.197 |  |
| LTH.C | RES(+)T1.C | 0.400 | 0.001 | * |
| NLEA.C | RES(+)T1.C | -0.220 | 0.070 |  |

| **Trait_1** | **Trait_2** | **corr.coef** | **p-value** |  |
| --- | --- | --- | --- | --- |
| RES(-)T1.C | RES(+)T1.C | 0.570 | 0.000 | * |
| RES(-)T2.C | RES(+)T1.C | 0.170 | 0.153 |  |
| RES(+)T1.C | RES(+)T2.C | -0.120 | 0.322 |  |
| IGR.F | RES(+)T1.C | 0.030 | 0.821 |  |
| LTH.F | RES(+)T1.C | 0.310 | 0.009 | * |
| NLEA.F | RES(+)T1.C | -0.240 | 0.047 | * |
| RES(+)T1.C | TOL_IGR.F | -0.190 | 0.127 |  |
| IGR.H | RES(+)T1.C | 0.120 | 0.317 |  |
| LTH.H | RES(+)T1.C | 0.020 | 0.867 |  |
| NLEA.H | RES(+)T1.C | -0.250 | 0.039 | * |
| RES(+)T1.C | TOL_IGR.H | -0.050 | 0.700 |  |
| ASYM.C | RES(+)T2.C | 0.020 | 0.887 |  |
| LA.C | RES(+)T2.C | 0.070 | 0.593 |  |
| LDI.C | RES(+)T2.C | 0.030 | 0.780 |  |
| LDMC.C | RES(+)T2.C | 0.230 | 0.059 |  |
| MGR.C | RES(+)T2.C | 0.120 | 0.320 |  |
| RES(+)T2.C | SLA.C | 0.200 | 0.107 |  |
| RES(+)T2.C | SSIZ.C | -0.140 | 0.237 |  |
| RES(+)T2.C | TGER.C | -0.100 | 0.392 |  |
| (-)XMID.C | RES(+)T2.C | -0.030 | 0.806 |  |
| ASYM.F | RES(+)T2.C | 0.120 | 0.338 |  |
| LA.F | RES(+)T2.C | 0.120 | 0.319 |  |
| LDI.F | RES(+)T2.C | 0.030 | 0.831 |  |
| LDMC.F | RES(+)T2.C | 0.300 | 0.012 | * |
| MGR.F | RES(+)T2.C | -0.130 | 0.304 |  |
| RES(-)T2.F | RES(+)T2.C | 0.080 | 0.502 |  |
| RES(+)T2.C | SLA.F | 0.070 | 0.559 |  |
| RES(+)T2.C | TOL_ASYM.F | 0.250 | 0.041 | * |
| RES(+)T2.C | TOL_MGR.F | -0.010 | 0.950 |  |
| RES(+)T2.C | TOL_(-)XMID.F | 0.000 | 0.986 |  |
| (-)XMID.F | RES(+)T2.C | -0.080 | 0.521 |  |
| ASYM.H | RES(+)T2.C | 0.100 | 0.393 |  |
| LA.H | RES(+)T2.C | 0.060 | 0.650 |  |
| LDI.H | RES(+)T2.C | 0.020 | 0.867 |  |
| LDMC.H | RES(+)T2.C | 0.350 | 0.003 | * |
| MGR.H | RES(+)T2.C | 0.070 | 0.590 |  |
| RES(+)T2.C | RES(+)T2.H | 0.180 | 0.129 |  |
| RES(+)T2.C | SLA.H | -0.080 | 0.522 |  |
| RES(+)T2.C | TOL_ASYM.H | 0.210 | 0.090 |  |
| RES(+)T2.C | TOL_MGR.H | 0.010 | 0.925 |  |
| RES(+)T2.C | TOL_(-)XMID.H | -0.240 | 0.051 |  |
| (-)XMID.H | RES(+)T2.C | -0.180 | 0.137 |  |
| IGR.C | RES(+)T2.C | -0.140 | 0.242 |  |
| LTH.C | RES(+)T2.C | -0.160 | 0.183 |  |
| NLEA.C | RES(+)T2.C | 0.010 | 0.938 |  |
| RES(-)T1.C | RES(+)T2.C | -0.040 | 0.738 |  |
| RES(-)T2.C | RES(+)T2.C | -0.110 | 0.369 |  |
| RES(+)T1.C | RES(+)T2.C | -0.120 | 0.322 |  |
| IGR.F | RES(+)T2.C | -0.070 | 0.545 |  |

| **Trait_1** | **Trait_2** | **corr.coef** | **p-value** |  |
| --- | --- | --- | --- | --- |
| LTH.F | RES(+)T2.C | -0.290 | 0.016 | * |
| NLEA.F | RES(+)T2.C | 0.060 | 0.614 |  |
| RES(+)T2.C | TOL_IGR.F | 0.120 | 0.320 |  |
| IGR.H | RES(+)T2.C | 0.020 | 0.879 |  |
| LTH.H | RES(+)T2.C | 0.030 | 0.804 |  |
| NLEA.H | RES(+)T2.C | 0.080 | 0.489 |  |
| RES(+)T2.C | TOL_IGR.H | 0.060 | 0.634 |  |
| ASYM.C | IGR.F | 0.030 | 0.801 |  |
| IGR.F | LA.C | 0.060 | 0.604 |  |
| IGR.F | LDI.C | 0.040 | 0.723 |  |
| IGR.F | LDMC.C | -0.150 | 0.223 |  |
| IGR.F | MGR.C | 0.130 | 0.298 |  |
| IGR.F | SLA.C | -0.010 | 0.924 |  |
| IGR.F | SSIZ.C | -0.170 | 0.159 |  |
| IGR.F | TGER.C | 0.060 | 0.650 |  |
| IGR.F | (-)XMID.C | -0.140 | 0.256 |  |
| ASYM.F | IGR.F | 0.020 | 0.886 |  |
| IGR.F | LA.F | 0.060 | 0.649 |  |
| IGR.F | LDI.F | 0.040 | 0.741 |  |
| IGR.F | LDMC.F | -0.110 | 0.348 |  |
| IGR.F | MGR.F | 0.120 | 0.324 |  |
| IGR.F | RES(-)T2.F | 0.050 | 0.662 |  |
| IGR.F | SLA.F | -0.020 | 0.858 |  |
| IGR.F | TOL_ASYM.F | -0.110 | 0.378 |  |
| IGR.F | TOL_MGR.F | 0.060 | 0.643 |  |
| IGR.F | TOL_(-)XMID.F | 0.050 | 0.704 |  |
| IGR.F | (-)XMID.F | -0.180 | 0.137 |  |
| ASYM.H | IGR.F | 0.100 | 0.426 |  |
| IGR.F | LA.H | 0.050 | 0.680 |  |
| IGR.F | LDI.H | 0.030 | 0.827 |  |
| IGR.F | LDMC.H | -0.220 | 0.064 |  |
| IGR.F | MGR.H | -0.130 | 0.280 |  |
| IGR.F | RES(+)T2.H | -0.260 | 0.029 | * |
| IGR.F | SLA.H | -0.010 | 0.921 |  |
| IGR.F | TOL_ASYM.H | 0.190 | 0.118 |  |
| IGR.F | TOL_MGR.H | -0.100 | 0.408 |  |
| IGR.F | TOL_(-)XMID.H | -0.090 | 0.485 |  |
| IGR.F | (-)XMID.H | -0.280 | 0.019 | * |
| IGR.C | IGR.F | -0.030 | 0.794 |  |
| IGR.F | LTH.C | 0.030 | 0.794 |  |
| IGR.F | NLEA.C | -0.150 | 0.207 |  |
| IGR.F | RES(-)T1.C | 0.260 | 0.031 | * |
| IGR.F | RES(-)T2.C | -0.040 | 0.719 |  |
| IGR.F | RES(+)T1.C | 0.030 | 0.821 |  |
| IGR.F | RES(+)T2.C | -0.070 | 0.545 |  |
| IGR.F | LTH.F | 0.070 | 0.579 |  |
| IGR.F | NLEA.F | -0.180 | 0.135 |  |
| IGR.F | TOL_IGR.F | 0.560 | 0.000 | * |
| IGR.F | IGR.H | 0.190 | 0.126 |  |

| **Trait_1** | **Trait_2** | **corr.coef** | **p-value** |  |
| --- | --- | --- | --- | --- |
| IGR.F | LTH.H | 0.250 | 0.041 | * |
| IGR.F | NLEA.H | -0.150 | 0.207 |  |
| IGR.F | TOL_IGR.H | 0.090 | 0.475 |  |
| ASYM.C | LTH.F | -0.110 | 0.371 |  |
| LA.C | LTH.F | -0.030 | 0.782 |  |
| LDI.C | LTH.F | -0.390 | 0.001 | * |
| LDMC.C | LTH.F | -0.250 | 0.035 | * |
| LTH.F | MGR.C | 0.020 | 0.896 |  |
| LTH.F | SLA.C | -0.330 | 0.005 | * |
| LTH.F | SSIZ.C | 0.290 | 0.015 | * |
| LTH.F | TGER.C | 0.000 | 0.969 |  |
| LTH.F | (-)XMID.C | 0.010 | 0.926 |  |
| ASYM.F | LTH.F | -0.180 | 0.129 |  |
| LA.F | LTH.F | -0.060 | 0.626 |  |
| LDI.F | LTH.F | -0.320 | 0.008 | * |
| LDMC.F | LTH.F | -0.340 | 0.005 | * |
| LTH.F | MGR.F | 0.000 | 0.989 |  |
| LTH.F | RES(-)T2.F | 0.050 | 0.654 |  |
| LTH.F | SLA.F | -0.310 | 0.009 | * |
| LTH.F | TOL_ASYM.F | -0.140 | 0.243 |  |
| LTH.F | TOL_MGR.F | -0.090 | 0.471 |  |
| LTH.F | TOL_(-)XMID.F | -0.150 | 0.222 |  |
| LTH.F | (-)XMID.F | -0.100 | 0.405 |  |
| ASYM.H | LTH.F | -0.150 | 0.206 |  |
| LA.H | LTH.F | -0.060 | 0.641 |  |
| LDI.H | LTH.F | -0.330 | 0.006 | * |
| LDMC.H | LTH.F | -0.310 | 0.010 | * |
| LTH.F | MGR.H | 0.010 | 0.946 |  |
| LTH.F | RES(+)T2.H | -0.040 | 0.736 |  |
| LTH.F | SLA.H | -0.150 | 0.226 |  |
| LTH.F | TOL_ASYM.H | -0.150 | 0.217 |  |
| LTH.F | TOL_MGR.H | 0.070 | 0.550 |  |
| LTH.F | TOL_(-)XMID.H | 0.210 | 0.079 |  |
| LTH.F | (-)XMID.H | 0.000 | 0.988 |  |
| IGR.C | LTH.F | 0.180 | 0.143 |  |
| LTH.C | LTH.F | 0.560 | 0.000 | * |
| LTH.F | NLEA.C | -0.160 | 0.186 |  |
| LTH.F | RES(-)T1.C | 0.490 | 0.000 | * |
| LTH.F | RES(-)T2.C | 0.050 | 0.656 |  |
| LTH.F | RES(+)T1.C | 0.310 | 0.009 | * |
| LTH.F | RES(+)T2.C | -0.290 | 0.016 | * |
| IGR.F | LTH.F | 0.070 | 0.579 |  |
| LTH.F | NLEA.F | -0.230 | 0.063 |  |
| LTH.F | TOL_IGR.F | -0.080 | 0.490 |  |
| IGR.H | LTH.F | 0.110 | 0.390 |  |
| LTH.F | LTH.H | 0.240 | 0.043 | * |
| LTH.F | NLEA.H | -0.270 | 0.022 | * |
| LTH.F | TOL_IGR.H | -0.010 | 0.912 |  |
| ASYM.C | NLEA.F | -0.360 | 0.003 | * |

| **Trait_1** | **Trait_2** | **corr.coef** | **p-value** |  |
| --- | --- | --- | --- | --- |
| LA.C | NLEA.F | -0.430 | 0.000 | * |
| LDI.C | NLEA.F | 0.000 | 0.975 |  |
| LDMC.C | NLEA.F | -0.110 | 0.375 |  |
| MGR.C | NLEA.F | 0.050 | 0.656 |  |
| NLEA.F | SLA.C | 0.340 | 0.004 | * |
| NLEA.F | SSIZ.C | -0.430 | 0.000 | * |
| NLEA.F | TGER.C | -0.290 | 0.017 | * |
| (-)XMID.C | NLEA.F | -0.090 | 0.487 |  |
| ASYM.F | NLEA.F | -0.300 | 0.013 | * |
| LA.F | NLEA.F | -0.470 | 0.000 | * |
| LDI.F | NLEA.F | 0.000 | 0.998 |  |
| LDMC.F | NLEA.F | 0.000 | 0.978 |  |
| MGR.F | NLEA.F | 0.020 | 0.851 |  |
| NLEA.F | RES(-)T2.F | 0.040 | 0.724 |  |
| NLEA.F | SLA.F | 0.380 | 0.001 | * |
| NLEA.F | TOL_ASYM.F | 0.150 | 0.228 |  |
| NLEA.F | TOL_MGR.F | -0.010 | 0.913 |  |
| NLEA.F | TOL_(-)XMID.F | 0.050 | 0.683 |  |
| (-)XMID.F | NLEA.F | 0.020 | 0.891 |  |
| ASYM.H | NLEA.F | -0.330 | 0.005 | * |
| LA.H | NLEA.F | -0.460 | 0.000 | * |
| LDI.H | NLEA.F | -0.040 | 0.746 |  |
| LDMC.H | NLEA.F | -0.160 | 0.190 |  |
| MGR.H | NLEA.F | -0.110 | 0.356 |  |
| NLEA.F | RES(+)T2.H | -0.040 | 0.748 |  |
| NLEA.F | SLA.H | 0.470 | 0.000 | * |
| NLEA.F | TOL_ASYM.H | -0.160 | 0.200 |  |
| NLEA.F | TOL_MGR.H | -0.080 | 0.515 |  |
| NLEA.F | TOL_(-)XMID.H | 0.100 | 0.404 |  |
| (-)XMID.H | NLEA.F | 0.000 | 0.990 |  |
| IGR.C | NLEA.F | -0.070 | 0.564 |  |
| LTH.C | NLEA.F | -0.290 | 0.017 | * |
| NLEA.C | NLEA.F | 0.870 | 0.000 | * |
| NLEA.F | RES(-)T1.C | -0.230 | 0.058 |  |
| NLEA.F | RES(-)T2.C | 0.100 | 0.412 |  |
| NLEA.F | RES(+)T1.C | -0.240 | 0.047 | * |
| NLEA.F | RES(+)T2.C | 0.060 | 0.614 |  |
| IGR.F | NLEA.F | -0.180 | 0.135 |  |
| LTH.F | NLEA.F | -0.230 | 0.063 |  |
| NLEA.F | TOL_IGR.F | 0.010 | 0.954 |  |
| IGR.H | NLEA.F | -0.140 | 0.239 |  |
| LTH.H | NLEA.F | -0.180 | 0.144 |  |
| NLEA.F | NLEA.H | 0.800 | 0.000 | * |
| NLEA.F | TOL_IGR.H | 0.000 | 0.984 |  |
| ASYM.C | TOL_IGR.F | -0.200 | 0.100 |  |
| LA.C | TOL_IGR.F | -0.200 | 0.099 |  |
| LDI.C | TOL_IGR.F | -0.210 | 0.086 |  |
| LDMC.C | TOL_IGR.F | -0.160 | 0.186 |  |
| MGR.C | TOL_IGR.F | -0.040 | 0.726 |  |

| **Trait_1** | **Trait_2** | **corr.coef** | **p-value** |  |
| --- | --- | --- | --- | --- |
| SLA.C | TOL_IGR.F | 0.110 | 0.385 |  |
| SSIZ.C | TOL_IGR.F | -0.260 | 0.031 | * |
| TGER.C | TOL_IGR.F | 0.170 | 0.168 |  |
| (-)XMID.C | TOL_IGR.F | -0.120 | 0.337 |  |
| ASYM.F | TOL_IGR.F | -0.280 | 0.020 | * |
| LA.F | TOL_IGR.F | -0.190 | 0.109 |  |
| LDI.F | TOL_IGR.F | -0.250 | 0.035 | * |
| LDMC.F | TOL_IGR.F | -0.090 | 0.484 |  |
| MGR.F | TOL_IGR.F | 0.000 | 0.999 |  |
| RES(-)T2.F | TOL_IGR.F | 0.130 | 0.288 |  |
| SLA.F | TOL_IGR.F | 0.060 | 0.647 |  |
| TOL_ASYM.F | TOL_IGR.F | -0.200 | 0.097 |  |
| TOL_IGR.F | TOL_MGR.F | 0.060 | 0.619 |  |
| TOL_IGR.F | TOL_(-)XMID.F | 0.110 | 0.381 |  |
| (-)XMID.F | TOL_IGR.F | 0.090 | 0.463 |  |
| ASYM.H | TOL_IGR.F | -0.210 | 0.079 |  |
| LA.H | TOL_IGR.F | -0.260 | 0.033 | * |
| LDI.H | TOL_IGR.F | -0.220 | 0.065 |  |
| LDMC.H | TOL_IGR.F | -0.150 | 0.210 |  |
| MGR.H | TOL_IGR.F | -0.020 | 0.901 |  |
| RES(+)T2.H | TOL_IGR.F | -0.070 | 0.573 |  |
| SLA.H | TOL_IGR.F | 0.060 | 0.646 |  |
| TOL_ASYM.H | TOL_IGR.F | -0.240 | 0.044 | * |
| TOL_IGR.F | TOL_MGR.H | -0.190 | 0.111 |  |
| TOL_IGR.F | TOL_(-)XMID.H | 0.060 | 0.596 |  |
| (-)XMID.H | TOL_IGR.F | -0.020 | 0.860 |  |
| IGR.C | TOL_IGR.F | -0.730 | 0.000 | * |
| LTH.C | TOL_IGR.F | -0.070 | 0.559 |  |
| NLEA.C | TOL_IGR.F | -0.020 | 0.886 |  |
| RES(-)T1.C | TOL_IGR.F | 0.090 | 0.454 |  |
| RES(-)T2.C | TOL_IGR.F | -0.190 | 0.126 |  |
| RES(+)T1.C | TOL_IGR.F | -0.190 | 0.127 |  |
| RES(+)T2.C | TOL_IGR.F | 0.120 | 0.320 |  |
| IGR.F | TOL_IGR.F | 0.560 | 0.000 | * |
| LTH.F | TOL_IGR.F | -0.080 | 0.490 |  |
| NLEA.F | TOL_IGR.F | 0.010 | 0.954 |  |
| IGR.H | TOL_IGR.F | 0.150 | 0.212 |  |
| LTH.H | TOL_IGR.F | 0.020 | 0.852 |  |
| NLEA.H | TOL_IGR.F | 0.010 | 0.912 |  |
| TOL_IGR.F | TOL_IGR.H | 0.150 | 0.211 |  |
| ASYM.C | IGR.H | -0.010 | 0.933 |  |
| IGR.H | LA.C | 0.040 | 0.721 |  |
| IGR.H | LDI.C | -0.140 | 0.242 |  |
| IGR.H | LDMC.C | 0.130 | 0.296 |  |
| IGR.H | MGR.C | 0.110 | 0.376 |  |
| IGR.H | SLA.C | -0.260 | 0.032 | * |
| IGR.H | SSIZ.C | 0.110 | 0.348 |  |
| IGR.H | TGER.C | 0.030 | 0.827 |  |
| IGR.H | (-)XMID.C | -0.010 | 0.910 |  |

| **Trait_1** | **Trait_2** | **corr.coef** | **p-value** |  |
| --- | --- | --- | --- | --- |
| ASYM.F | IGR.H | -0.040 | 0.725 |  |
| IGR.H | LA.F | 0.040 | 0.754 |  |
| IGR.H | LDI.F | -0.120 | 0.319 |  |
| IGR.H | LDMC.F | 0.050 | 0.669 |  |
| IGR.H | MGR.F | -0.040 | 0.766 |  |
| IGR.H | RES(-)T2.F | 0.130 | 0.286 |  |
| IGR.H | SLA.F | -0.240 | 0.045 | * |
| IGR.H | TOL_ASYM.F | -0.210 | 0.086 |  |
| IGR.H | TOL_MGR.F | -0.120 | 0.329 |  |
| IGR.H | TOL_(-)XMID.F | -0.060 | 0.618 |  |
| IGR.H | (-)XMID.F | 0.120 | 0.346 |  |
| ASYM.H | IGR.H | 0.040 | 0.714 |  |
| IGR.H | LA.H | 0.040 | 0.742 |  |
| IGR.H | LDI.H | -0.150 | 0.227 |  |
| IGR.H | LDMC.H | 0.010 | 0.932 |  |
| IGR.H | MGR.H | 0.030 | 0.789 |  |
| IGR.H | RES(+)T2.H | -0.040 | 0.748 |  |
| IGR.H | SLA.H | -0.090 | 0.440 |  |
| IGR.H | TOL_ASYM.H | -0.020 | 0.898 |  |
| IGR.H | TOL_MGR.H | -0.040 | 0.752 |  |
| IGR.H | TOL_(-)XMID.H | 0.080 | 0.523 |  |
| IGR.H | (-)XMID.H | 0.010 | 0.942 |  |
| IGR.C | IGR.H | -0.080 | 0.530 |  |
| IGR.H | LTH.C | 0.100 | 0.411 |  |
| IGR.H | NLEA.C | -0.190 | 0.127 |  |
| IGR.H | RES(-)T1.C | 0.160 | 0.182 |  |
| IGR.H | RES(-)T2.C | 0.290 | 0.015 | * |
| IGR.H | RES(+)T1.C | 0.120 | 0.317 |  |
| IGR.H | RES(+)T2.C | 0.020 | 0.879 |  |
| IGR.F | IGR.H | 0.190 | 0.126 |  |
| IGR.H | LTH.F | 0.110 | 0.390 |  |
| IGR.H | NLEA.F | -0.140 | 0.239 |  |
| IGR.H | TOL_IGR.F | 0.150 | 0.212 |  |
| IGR.H | LTH.H | 0.040 | 0.761 |  |
| IGR.H | NLEA.H | -0.160 | 0.178 |  |
| IGR.H | TOL_IGR.H | 0.600 | 0.000 | * |
| ASYM.C | LTH.H | 0.040 | 0.720 |  |
| LA.C | LTH.H | 0.120 | 0.312 |  |
| LDI.C | LTH.H | 0.040 | 0.728 |  |
| LDMC.C | LTH.H | 0.020 | 0.889 |  |
| LTH.H | MGR.C | 0.050 | 0.667 |  |
| LTH.H | SLA.C | -0.180 | 0.133 |  |
| LTH.H | SSIZ.C | 0.120 | 0.336 |  |
| LTH.H | TGER.C | 0.060 | 0.642 |  |
| LTH.H | (-)XMID.C | 0.090 | 0.464 |  |
| ASYM.F | LTH.H | 0.070 | 0.591 |  |
| LA.F | LTH.H | 0.140 | 0.260 |  |
| LDI.F | LTH.H | 0.020 | 0.889 |  |
| LDMC.F | LTH.H | -0.090 | 0.457 |  |

| **Trait_1** | **Trait_2** | **corr.coef** | **p-value** |  |
| --- | --- | --- | --- | --- |
| LTH.H | MGR.F | -0.050 | 0.665 |  |
| LTH.H | RES(-)T2.F | 0.150 | 0.234 |  |
| LTH.H | SLA.F | -0.170 | 0.169 |  |
| LTH.H | TOL_ASYM.F | -0.070 | 0.553 |  |
| LTH.H | TOL_MGR.F | -0.030 | 0.796 |  |
| LTH.H | TOL_(-)XMID.F | 0.070 | 0.551 |  |
| LTH.H | (-)XMID.F | 0.020 | 0.900 |  |
| ASYM.H | LTH.H | 0.110 | 0.371 |  |
| LA.H | LTH.H | 0.100 | 0.411 |  |
| LDI.H | LTH.H | 0.010 | 0.928 |  |
| LDMC.H | LTH.H | 0.100 | 0.418 |  |
| LTH.H | MGR.H | 0.110 | 0.385 |  |
| LTH.H | RES(+)T2.H | -0.080 | 0.531 |  |
| LTH.H | SLA.H | -0.240 | 0.046 | * |
| LTH.H | TOL_ASYM.H | 0.090 | 0.483 |  |
| LTH.H | TOL_MGR.H | 0.210 | 0.087 |  |
| LTH.H | TOL_(-)XMID.H | 0.010 | 0.929 |  |
| LTH.H | (-)XMID.H | 0.020 | 0.859 |  |
| IGR.C | LTH.H | 0.120 | 0.333 |  |
| LTH.C | LTH.H | 0.110 | 0.390 |  |
| LTH.H | NLEA.C | -0.240 | 0.044 | * |
| LTH.H | RES(-)T1.C | 0.250 | 0.038 | * |
| LTH.H | RES(-)T2.C | -0.100 | 0.434 |  |
| LTH.H | RES(+)T1.C | 0.020 | 0.867 |  |
| LTH.H | RES(+)T2.C | 0.030 | 0.804 |  |
| IGR.F | LTH.H | 0.250 | 0.041 | * |
| LTH.F | LTH.H | 0.240 | 0.043 | * |
| LTH.H | NLEA.F | -0.180 | 0.144 |  |
| LTH.H | TOL_IGR.F | 0.020 | 0.852 |  |
| IGR.H | LTH.H | 0.040 | 0.761 |  |
| LTH.H | NLEA.H | -0.190 | 0.110 |  |
| LTH.H | TOL_IGR.H | 0.030 | 0.801 |  |
| ASYM.C | NLEA.H | -0.290 | 0.015 | * |
| LA.C | NLEA.H | -0.420 | 0.000 | * |
| LDI.C | NLEA.H | 0.020 | 0.898 |  |
| LDMC.C | NLEA.H | -0.060 | 0.602 |  |
| MGR.C | NLEA.H | 0.080 | 0.521 |  |
| NLEA.H | SLA.C | 0.400 | 0.001 | * |
| NLEA.H | SSIZ.C | -0.430 | 0.000 | * |
| NLEA.H | TGER.C | -0.250 | 0.042 | * |
| (-)XMID.C | NLEA.H | -0.100 | 0.414 |  |
| ASYM.F | NLEA.H | -0.260 | 0.033 | * |
| LA.F | NLEA.H | -0.440 | 0.000 | * |
| LDI.F | NLEA.H | 0.010 | 0.953 |  |
| LDMC.F | NLEA.H | 0.070 | 0.595 |  |
| MGR.F | NLEA.H | 0.050 | 0.708 |  |
| NLEA.H | RES(-)T2.F | -0.030 | 0.786 |  |
| NLEA.H | SLA.F | 0.440 | 0.000 | * |
| NLEA.H | TOL_ASYM.F | 0.130 | 0.288 |  |

| **Trait_1** | **Trait_2** | **corr.coef** | **p-value** |  |
| --- | --- | --- | --- | --- |
| NLEA.H | TOL_MGR.F | -0.060 | 0.637 |  |
| NLEA.H | TOL_(-)XMID.F | 0.020 | 0.854 |  |
| (-)XMID.F | NLEA.H | 0.040 | 0.759 |  |
| ASYM.H | NLEA.H | -0.300 | 0.013 | * |
| LA.H | NLEA.H | -0.430 | 0.000 | * |
| LDI.H | NLEA.H | -0.040 | 0.717 |  |
| LDMC.H | NLEA.H | -0.160 | 0.176 |  |
| MGR.H | NLEA.H | -0.200 | 0.102 |  |
| NLEA.H | RES(+)T2.H | 0.110 | 0.360 |  |
| NLEA.H | SLA.H | 0.490 | 0.000 | * |
| NLEA.H | TOL_ASYM.H | -0.070 | 0.557 |  |
| NLEA.H | TOL_MGR.H | -0.220 | 0.075 |  |
| NLEA.H | TOL_(-)XMID.H | 0.240 | 0.048 | * |
| (-)XMID.H | NLEA.H | 0.100 | 0.416 |  |
| IGR.C | NLEA.H | 0.030 | 0.816 |  |
| LTH.C | NLEA.H | -0.410 | 0.000 | * |
| NLEA.C | NLEA.H | 0.920 | 0.000 | * |
| NLEA.H | RES(-)T1.C | -0.330 | 0.006 | * |
| NLEA.H | RES(-)T2.C | 0.140 | 0.238 |  |
| NLEA.H | RES(+)T1.C | -0.250 | 0.039 | * |
| NLEA.H | RES(+)T2.C | 0.080 | 0.489 |  |
| IGR.F | NLEA.H | -0.150 | 0.207 |  |
| LTH.F | NLEA.H | -0.270 | 0.022 | * |
| NLEA.F | NLEA.H | 0.800 | 0.000 | * |
| NLEA.H | TOL_IGR.F | 0.010 | 0.912 |  |
| IGR.H | NLEA.H | -0.160 | 0.178 |  |
| LTH.H | NLEA.H | -0.190 | 0.110 |  |
| NLEA.H | TOL_IGR.H | -0.060 | 0.623 |  |
| ASYM.C | TOL_IGR.H | 0.050 | 0.694 |  |
| LA.C | TOL_IGR.H | 0.080 | 0.536 |  |
| LDI.C | TOL_IGR.H | 0.030 | 0.803 |  |
| LDMC.C | TOL_IGR.H | 0.110 | 0.369 |  |
| MGR.C | TOL_IGR.H | 0.180 | 0.149 |  |
| SLA.C | TOL_IGR.H | -0.060 | 0.633 |  |
| SSIZ.C | TOL_IGR.H | 0.020 | 0.856 |  |
| TGER.C | TOL_IGR.H | 0.020 | 0.871 |  |
| (-)XMID.C | TOL_IGR.H | 0.060 | 0.636 |  |
| ASYM.F | TOL_IGR.H | 0.080 | 0.534 |  |
| LA.F | TOL_IGR.H | 0.080 | 0.538 |  |
| LDI.F | TOL_IGR.H | 0.040 | 0.763 |  |
| LDMC.F | TOL_IGR.H | 0.120 | 0.321 |  |
| MGR.F | TOL_IGR.H | 0.060 | 0.609 |  |
| RES(-)T2.F | TOL_IGR.H | 0.090 | 0.452 |  |
| SLA.F | TOL_IGR.H | -0.060 | 0.649 |  |
| TOL_ASYM.F | TOL_IGR.H | -0.070 | 0.588 |  |
| TOL_IGR.H | TOL_MGR.F | -0.170 | 0.157 |  |
| TOL_IGR.H | TOL_(-)XMID.F | -0.070 | 0.590 |  |
| (-)XMID.F | TOL_IGR.H | 0.150 | 0.211 |  |
| ASYM.H | TOL_IGR.H | 0.110 | 0.369 |  |

| **Trait_1** | **Trait_2** | **corr.coef** | **p-value** |  |
| --- | --- | --- | --- | --- |
| LA.H | TOL_IGR.H | 0.030 | 0.822 |  |
| LDI.H | TOL_IGR.H | -0.050 | 0.703 |  |
| LDMC.H | TOL_IGR.H | 0.030 | 0.836 |  |
| MGR.H | TOL_IGR.H | 0.150 | 0.222 |  |
| RES(+)T2.H | TOL_IGR.H | -0.100 | 0.415 |  |
| SLA.H | TOL_IGR.H | -0.020 | 0.892 |  |
| TOL_ASYM.H | TOL_IGR.H | 0.060 | 0.603 |  |
| TOL_IGR.H | TOL_MGR.H | 0.070 | 0.593 |  |
| TOL_IGR.H | TOL_(-)XMID.H | 0.040 | 0.724 |  |
| (-)XMID.H | TOL_IGR.H | 0.130 | 0.291 |  |
| IGR.C | TOL_IGR.H | -0.130 | 0.284 |  |
| LTH.C | TOL_IGR.H | -0.090 | 0.475 |  |
| NLEA.C | TOL_IGR.H | -0.130 | 0.276 |  |
| RES(-)T1.C | TOL_IGR.H | -0.010 | 0.918 |  |
| RES(-)T2.C | TOL_IGR.H | 0.180 | 0.143 |  |
| RES(+)T1.C | TOL_IGR.H | -0.050 | 0.700 |  |
| RES(+)T2.C | TOL_IGR.H | 0.060 | 0.634 |  |
| IGR.F | TOL_IGR.H | 0.090 | 0.475 |  |
| LTH.F | TOL_IGR.H | -0.010 | 0.912 |  |
| NLEA.F | TOL_IGR.H | 0.000 | 0.984 |  |
| TOL_IGR.F | TOL_IGR.H | 0.150 | 0.211 |  |
| IGR.H | TOL_IGR.H | 0.600 | 0.000 | * |
| LTH.H | TOL_IGR.H | 0.030 | 0.801 |  |
| NLEA.H | TOL_IGR.H | -0.060 | 0.623 |  |

**
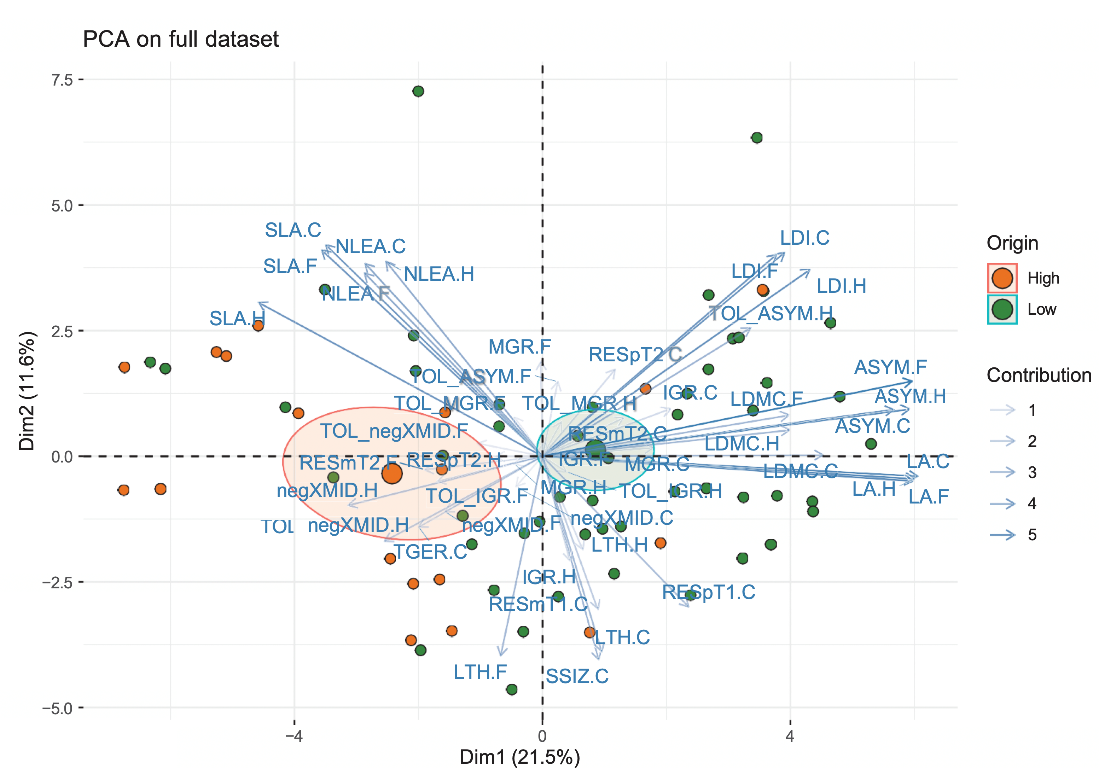
**

**
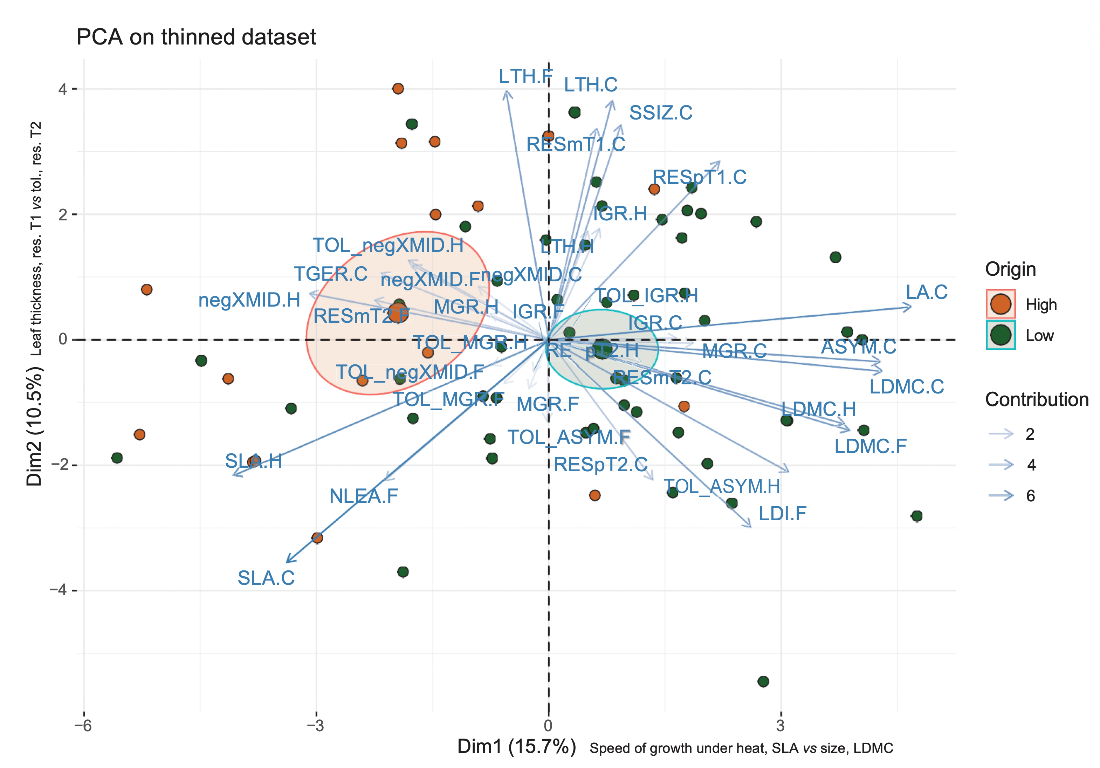
**

**Figure S6.** Biplots representing the correlation structure among traits measured under the three growth treatments (F for regular frost, C for control/mild, H for regular heat) that contributed to the first two principle components (PCs) of principal component analyses. Results for the full dataset (top) and the thinned dataset using a VIF threshold of 10 (bottom) are shown. Arrows in darker color indicate higher trait contribution. Each point represents the position of a species with either a low- (green) or high-elevation distribution (brown). The larger dots indicate the averages of low- and high-elevation species and the ellipses the concentrations (default settings, level = 0.95).


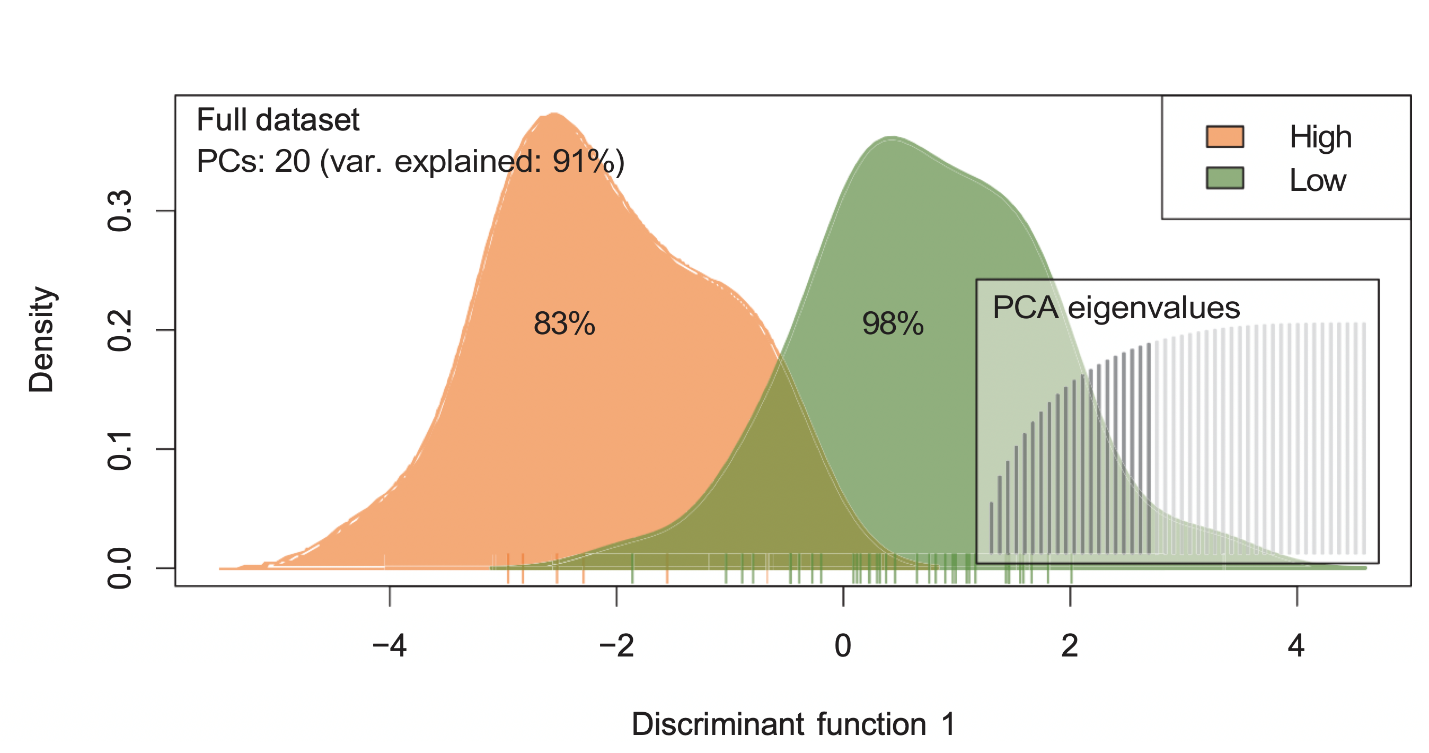


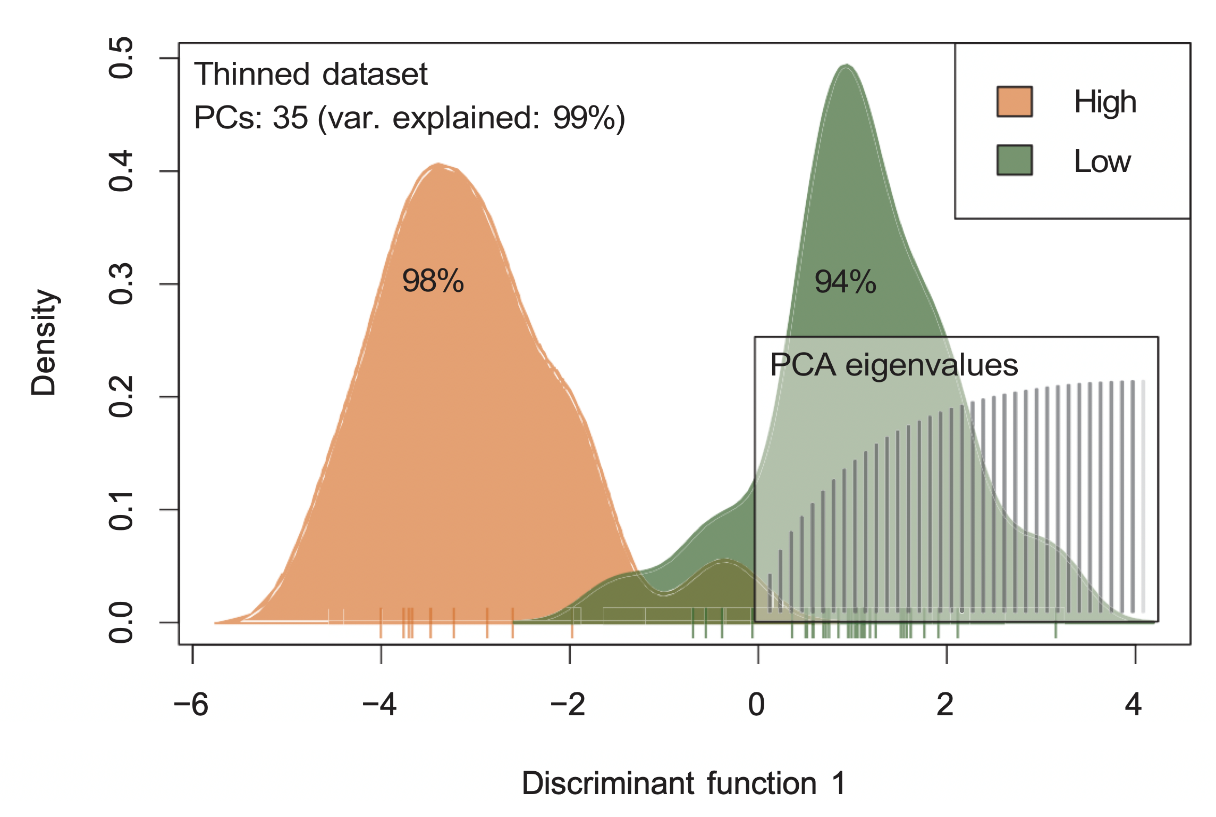


**Figure S7.** Density distributions of high- (in brown) and low-elevation species (in green) on discriminant function 1 revealed by discriminant analysis of principal components (DAPC). The panel on the top shows results for the full data set when the first 20 PCs were kept. The panel on the bottom shows results for the thinned data set (VIF threshold of 10) when the first 35 PCs were kept. The graphs on the lower right of each panel show the cumulative variance explained by the PCs, in order of contribution.


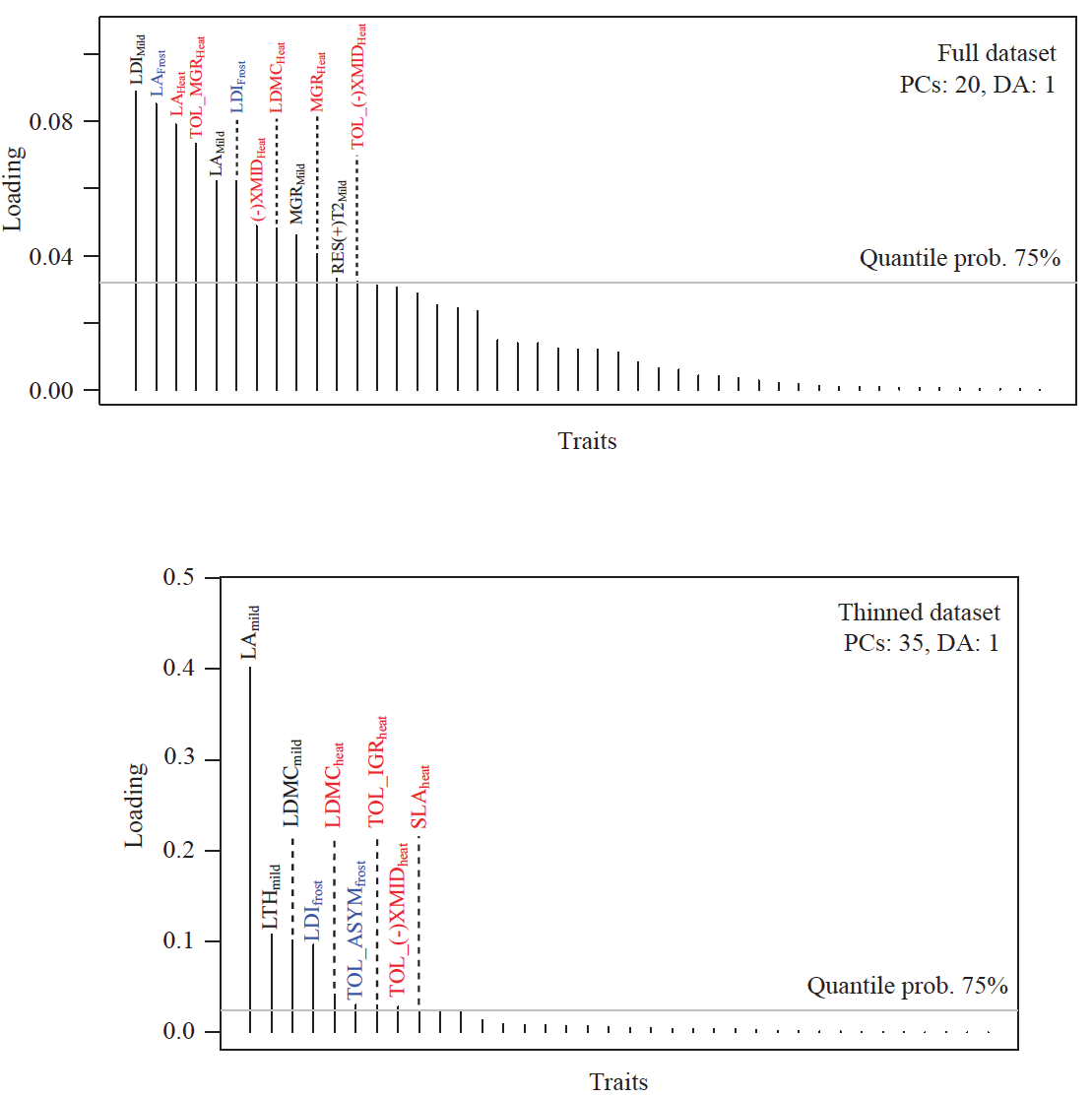


**Figure S8.** Loading by traits to discriminant function 1. The panel on the top shows results for the full data set when the first 20 PCs were kept for discriminant analysis of principle components (DAPC). The panel on the bottom shows results for the thinned data set (VIF threshold of 10) when the first 35 PCs were kept for DAPC. The most discriminating traits are labelled. The colors represent the three growth environments in which traits were assessed (blue for Frost, black for Mild/control, red for Heat). The black horizontal line distinguishes between traits having a loading > than the 75% quantile.


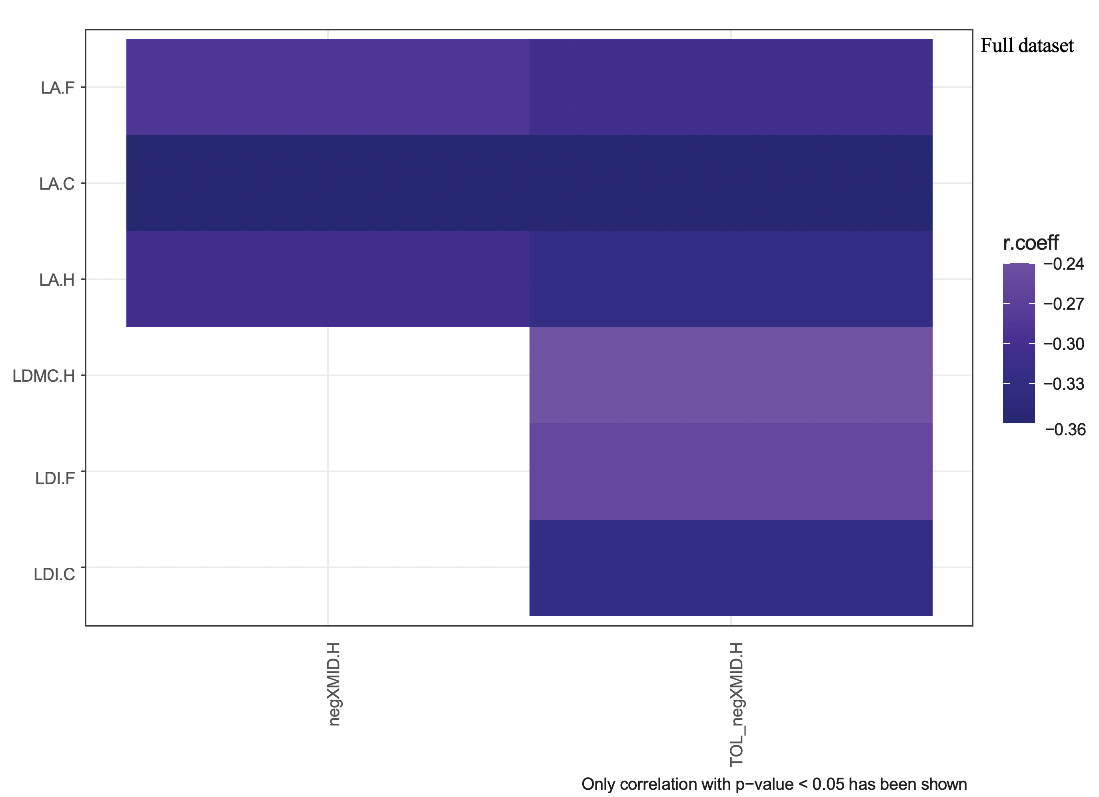


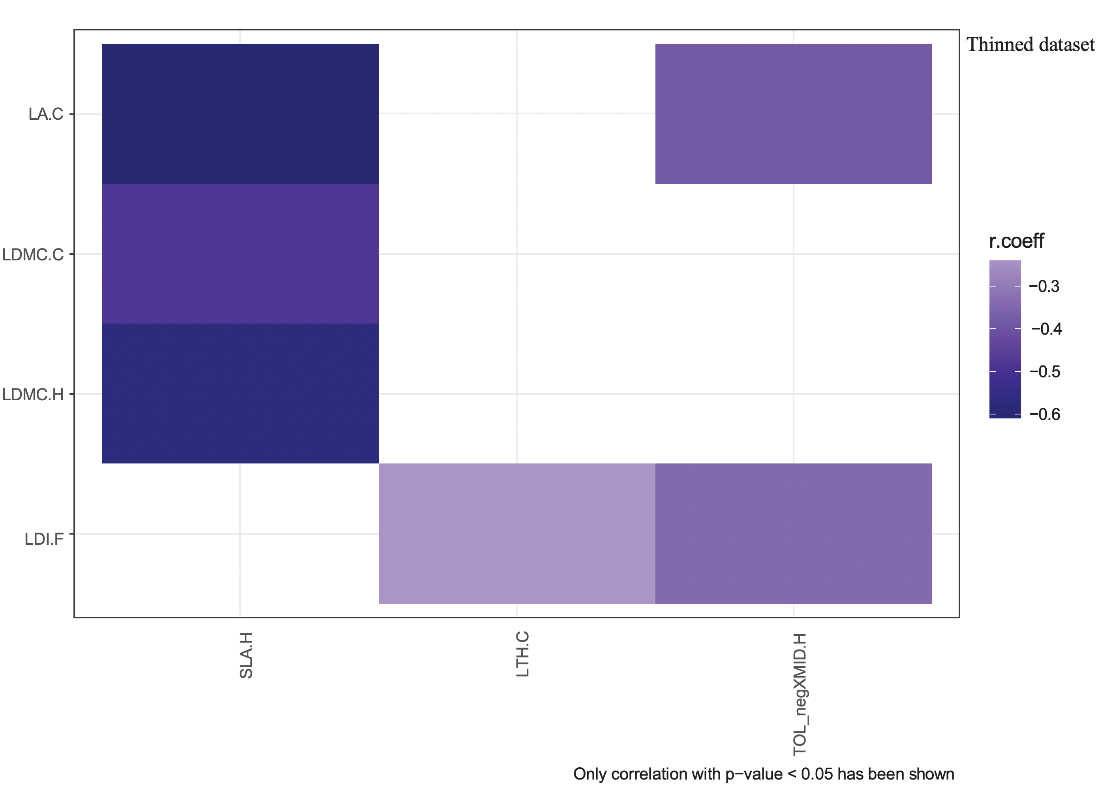


**Figure S9.** Significant negative correlations among the most discriminating traits distinguishing high- from low-elevation species. The panel on the top shows results for the full data set when the first 20 PCs were kept for DAPC. The panel on the bottom shows results for the thinned data set (VIF threshold of 10) when the first 35 PCs were kept for DAPC. Cutoff for significance was p < 0.05.


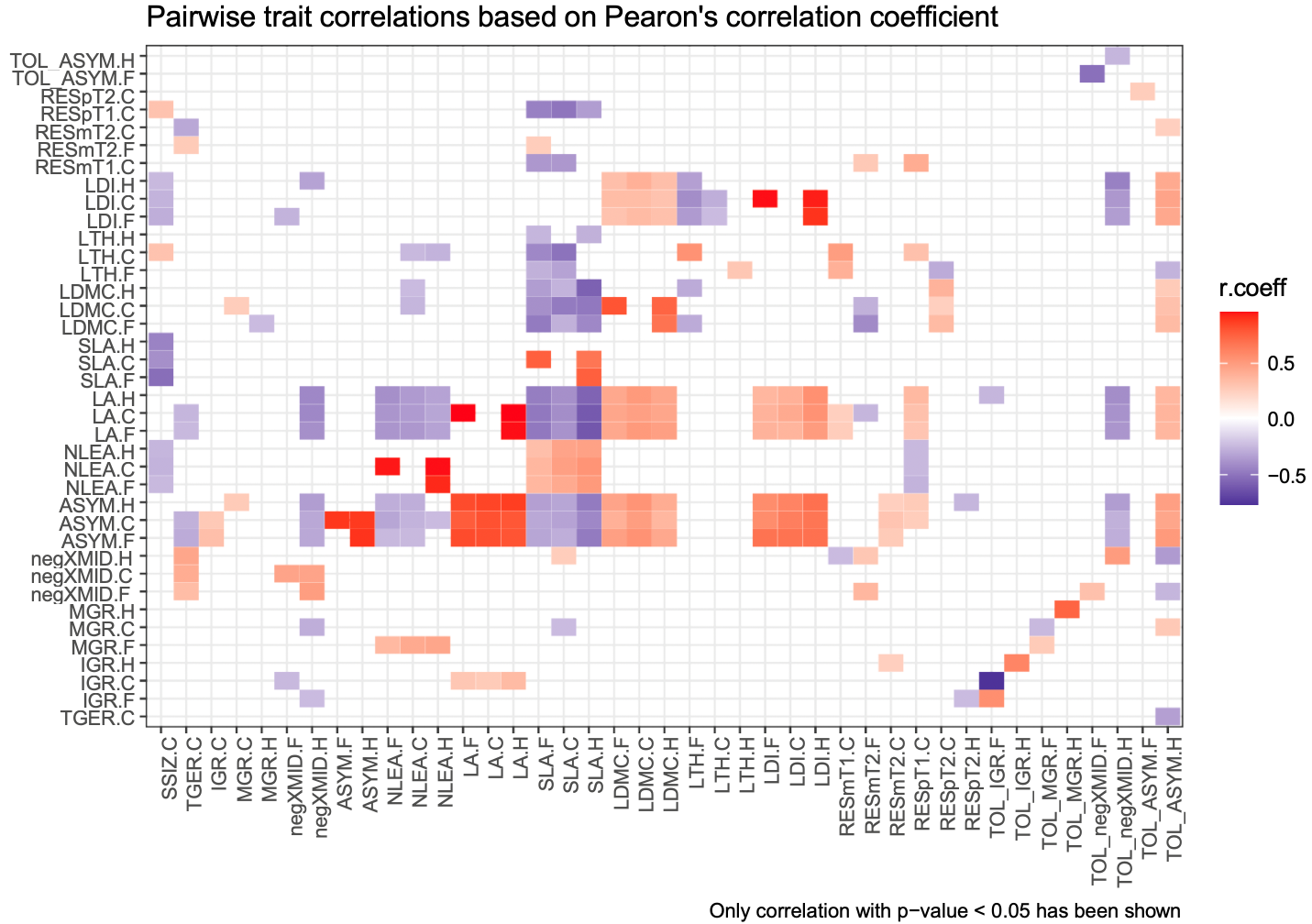


**Figure S10.** Correlation structure among traits expressed in the three growth treatments (based on species means between S1 and S2). Only significant Pearson’s correlation coefficients are indicated (p < 0.05). Positive and negative relationships are highlighted in red or velvet color, respectively.
